# Peptidic boronic acids are potent cell-permeable inhibitors of the malaria parasite egress serine protease SUB1

**DOI:** 10.1073/pnas.2022696118

**Published:** 2021-05-11

**Authors:** Elina Lidumniece, Chrislaine Withers-Martinez, Fiona Hackett, Christine R. Collins, Abigail J. Perrin, Konstantinos Koussis, Claudine Bisson, Michael J. Blackman, Aigars Jirgensons

**Affiliations:** ^a^Latvian Institute of Organic Synthesis, Riga LV-1006, Latvia;; ^b^Malaria Biochemistry Laboratory, The Francis Crick Institute, London NW1 1AT, United Kingdom;; ^c^Department of Biological Sciences, Institute of Structural and Molecular Biology, Birkbeck College, University of London, London WC1E 7HX, United Kingdom;; ^d^Centre for Ultrastructural Imaging, Kings College London, London SE1 1UL, United Kingdom;; ^e^Faculty of Infectious and Tropical Diseases, London School of Hygiene & Tropical Medicine, London WC1E 7HT, United Kingdom

**Keywords:** serine protease, boronic acid, egress, *Plasmodium falciparum*, malaria

## Abstract

Malaria is a devastating infectious disease, which causes over 400,000 deaths per annum and impacts the lives of nearly half the world’s population. The causative agent, a protozoan parasite, replicates within red blood cells (RBCs), eventually destroying the cells in a lytic process called egress to release a new generation of parasites. These invade fresh RBCs to repeat the cycle. Egress is regulated by an essential parasite subtilisin-like serine protease called SUB1. Here, we describe the development and optimization of substrate-based peptidic boronic acids that inhibit *Plasmodium falciparum* SUB1 with low nanomolar potency. Structural optimization generated membrane-permeable, slow off-rate inhibitors that prevent *P*. *falciparum* egress through direct inhibition of SUB1 activity and block parasite replication in vitro at submicromolar concentrations. Our results validate SUB1 as a potential target for a new class of antimalarial drugs designed to prevent parasite replication and disease progression.

## Significance

Malaria remains a major global health threat. In the face of increasing resistance to available chemotherapeutics, new antimalarial drugs with new modes of action are urgently needed. The causative agent of malaria is a single-celled parasite that invades and replicates within red blood cells. Escape from the red cell, a process called egress, involves a proteolytic pathway triggered by an essential parasite subtilisin-like serine protease called SUB1. Here, we describe the development and rational optimization of a potent, membrane-permeable substrate-based boronic acid compounds that block egress and parasite proliferation by direct inhibition of SUB1 activity. The compounds could form the basis of a new type of antimalarial medicine that would both protect against infection and treat disease.

Malaria, a disease caused by obligate intracellular parasites of the genus *Plasmodium*, is a global health problem threatening more than half the earth’s population (1). Recent decades have seen a considerable reduction in the incidence of clinical malaria and malaria-related mortality, largely due to the availability of efficacious chemotherapies and control of the mosquito vector (2). However, efforts toward malaria eradication are impeded by the alarming spread of drug-resistant parasites, rendering existing drugs ineffective in many regions (3, 4). Of particular concern, resistance has now been reported to nearly all clinically used antimalarial drugs including artemisinins, the current front line drug class (5). There is therefore an urgent need to bolster the antimalarial drug arsenal with new chemotherapeutics, particularly those with as yet unexploited mechanisms of action.

Clinical malaria results from repeated rounds of replication of the parasite in circulating red blood cells (RBCs). Merozoites invade the cells and divide asexually within a membrane-bound parasitophorous vacuole (PV) to produce a mature multinucleated form called a schizont. This then undergoes segmentation to generate 16 or more daughter merozoites, which are eventually released through a lytic process called egress, in the process destroying the infected RBC. Shortly before egress, activation of a parasite cyclic GMP-dependent protein kinase called PKG induces the discharge of a subtilisin-like serine protease called SUB1 from specialized merozoite secretory organelles called exonemes (6, 7). Upon its release into the PV lumen, SUB1 rapidly cleaves and activates a number of PV-resident and merozoite surface proteins, leading within minutes to explosive rupture of the PV membrane (PVM) and RBC membrane to allow merozoite release (8–12). The free parasites immediately invade fresh RBCs to repeat the cycle.

**Fig. 1.**
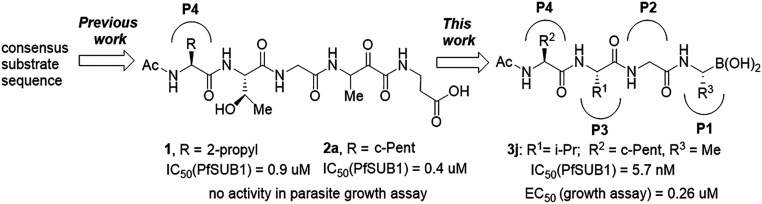
Development of rationally designed peptidic PfSUB1 inhibitors.

All *Plasmodium* species, including the most important human malaria pathogens *Plasmodium falciparum*, *Plasmodium vivax*, and *Plasmodium knowlesi*, possess a single ortholog of SUB1 with similar (though not identical) substrate specificity (13). Genetic experiments have shown that SUB1 is indispensable for parasite survival, with *SUB1* gene disruption leading in asexual blood stages and the preceding liver stages of infection to a complete block in merozoite egress (12, 14, 15). This, together with the lack of structural resemblance of SUB1 to human serine proteases (16, 17), has focused interest on SUB1 as an attractive pharmacological target for antimalarial drug discovery. However, the identification of potent drug-like SUB1 inhibitors has proven to be a difficult task. Attempts to identify ligands of SUB1 by screening of synthetic or natural product libraries, and through in silico screening, met with limited success (6, 18, 19), probably due to the relatively shallow and elongated cavity of the enzyme active site (16, 17). We have previously reported the rational design of peptidic ketoamide inhibitors of *P*. *falciparum* SUB1 (PfSUB1) based on the substrate specificity of the enzyme (Fig. 1) (13, 20). Preliminary structure-activity relationships analysis of these inhibitors revealed a tetrapeptide mimic on the nonprime side and an oxycarbonylethyl group on the prime side as structural features required to attain submicromolar inhibitory potency. Given the capacity of boronic acids to form strong covalent but reversible bonds with the catalytic Ser residue of serine proteases, here we have investigated peptidic boronic acids as PfSUB1 inhibitors. These efforts have generated nanomolar PfSUB1 inhibitors that can access PfSUB1 in the intraerythrocytic parasite and prevent parasite replication through direct inhibition of egress.

## Results

### Discovery of Potent Substrate-Based Peptidyl Boronic Acid Inhibitors of PfSUB1.

We previously described the development of a fluorescence-based in vitro assay suitable for the evaluation of substrate-based PfSUB1 inhibitors, using recombinant PfSUB1 (rPfSUB1) and fluorogenic peptide substrates based on cleavage sites within endogenous protein substrates of PfSUB1 (13, 21). In our earlier work (13, 20), we used the assay to identify a substrate-based pentapeptidic α-ketoamide with a P4 Ile residue and P2 Gly residue as our most potent inhibitor **1** (IC_50_ ∼900 nM; Fig. 1). Unfortunately, this and related α-ketoamides showed no antiparasite activity in vitro. This was perhaps unsurprising due to the high molecular mass and polar nature of these compounds, including the presence of a carboxylic acid moiety that was designed to mimic endogenous PfSUB1 protein substrates by interacting with the basic S’ surface of the PfSUB1 active-site cleft (16). Collectively, these features likely rendered the compounds poorly membrane penetrant.

To build on that work, we first explored a range of P4 substituents of the *N*-acetyl peptidyl α-ketoamide scaffold, maintaining the P1 Ala, P2 Gly, and P3 Thr sidechains unaltered. Replacement of the P4 Ile side chain with a cyclopentane improved potency, resulting in an peptidic α-ketoamide **2a** with an IC_50_ ∼370 nM (*SI Appendix*, Table S1). Reasoning that the exploration of alternative warheads with known activity against serine proteases might prove fruitful, we replaced the α-ketoamide functionality of this compound with a boronic acid warhead, in the process removing the prime side carboxylate. This resulted in compound **3a**, which gratifyingly demonstrated an ∼sevenfold increase in potency over the best α-ketoamide (Table 1). Combining the features of the α-ketoamide and compound **3a** by adding back the P4 cyclopentane improved potency by a further ∼13-fold, leading to the low nanomolar IC_50_ compound **3b** (Table 1).

**Table 1.** PfSUB1 enzyme inhibitory and parasite growth inhibitory potency of peptidic boronic acids

Entry	Compound	Structure	IC_50_ (nM) (rPfSUB1)*	EC_50_ (μM) (parasite growth)^†^
1	**3a**	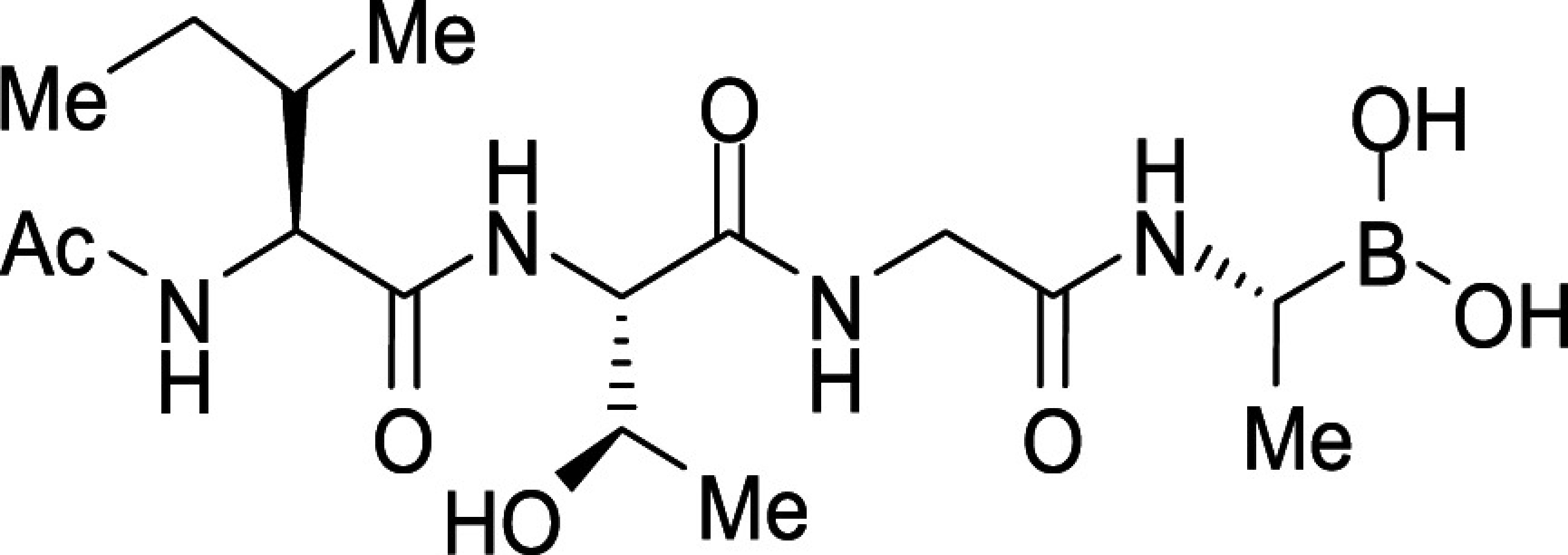	69.4 ± 1.2	1.8 ± 0.6
2	**3b**	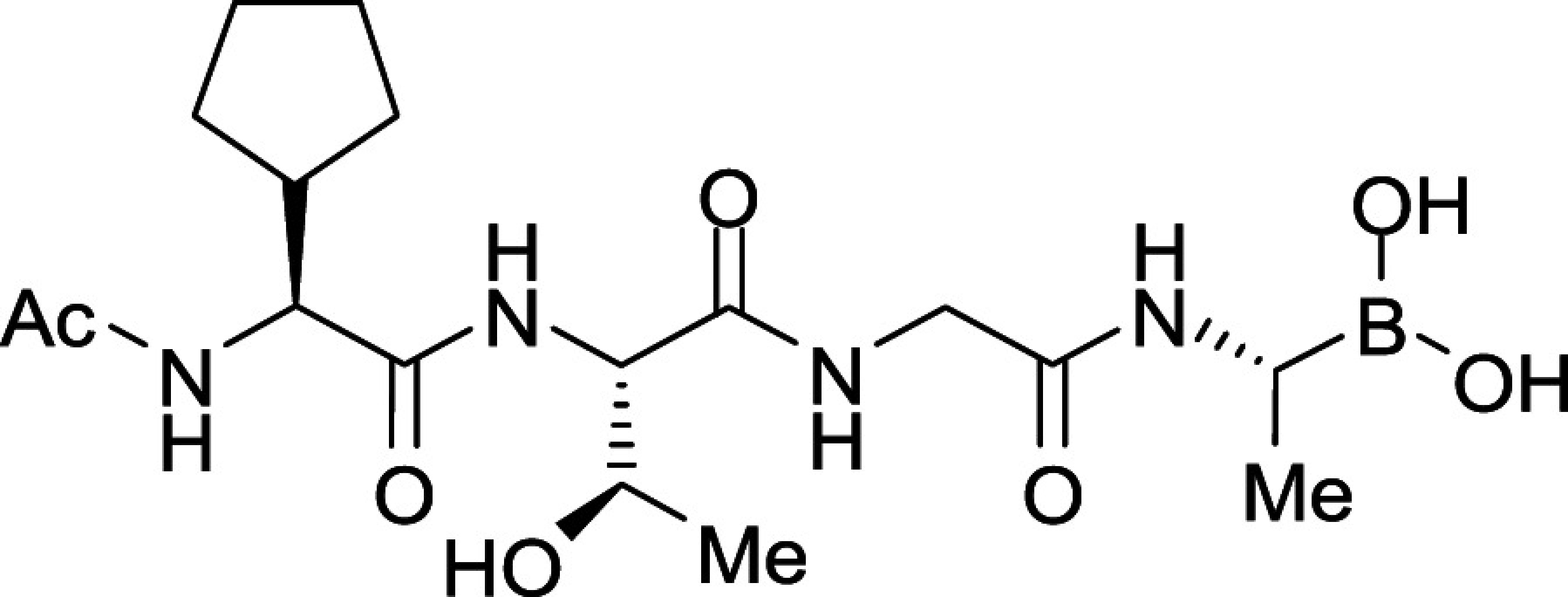	9.3 ± 0.5	2.3 ± 1.4
3	**3c**	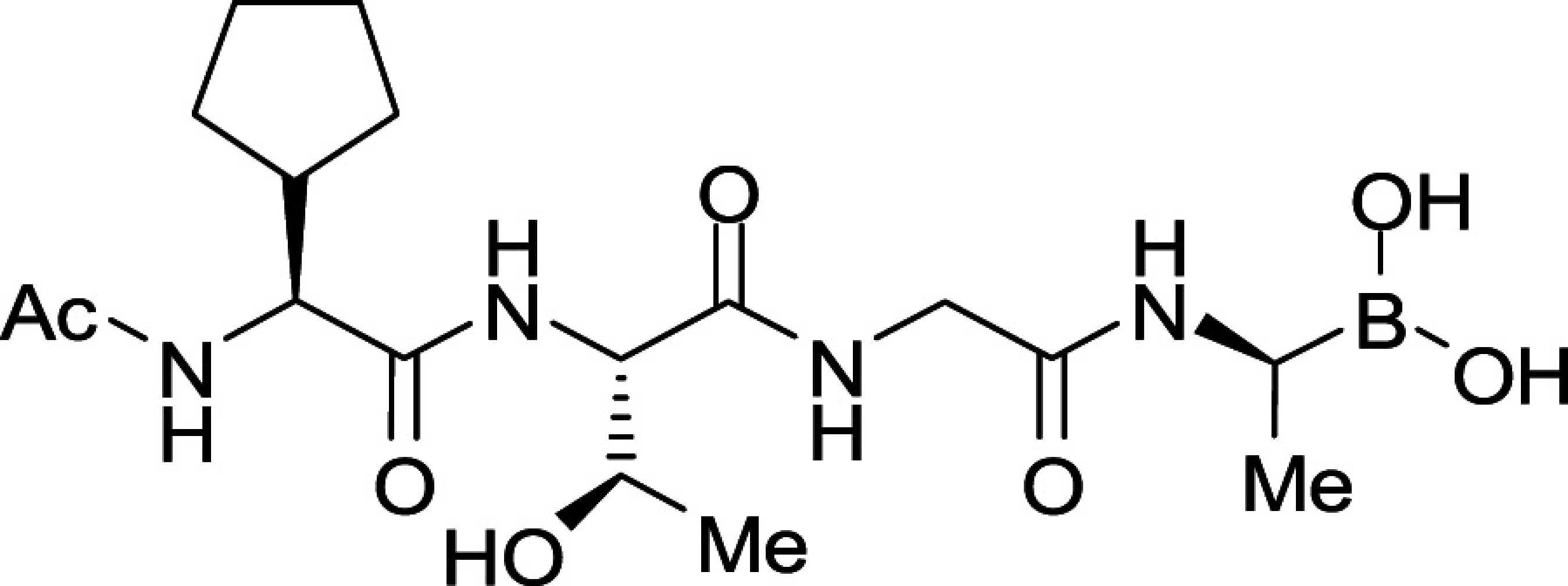	60.1 ± 2.1	18.4 ± 1.8
4	**3d**	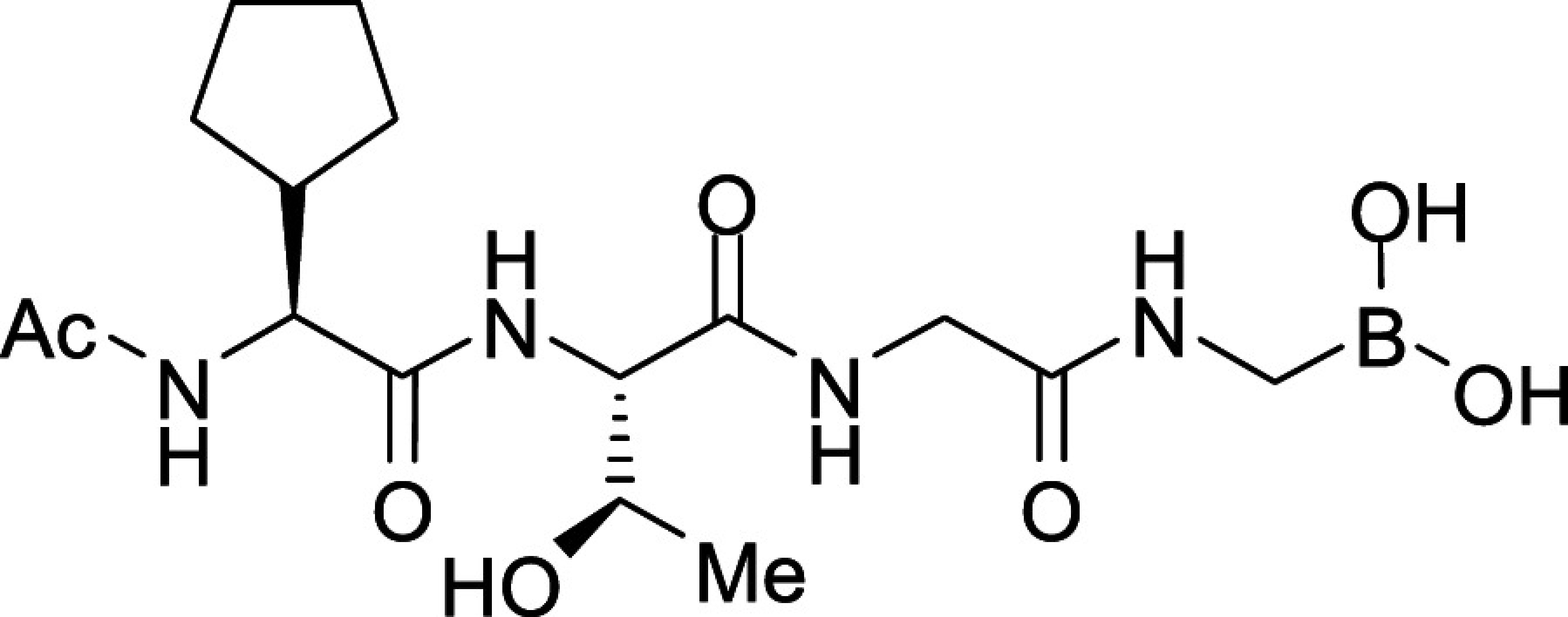	54.3 ± 1.1	1.9 ± 0.4
5	**3e**	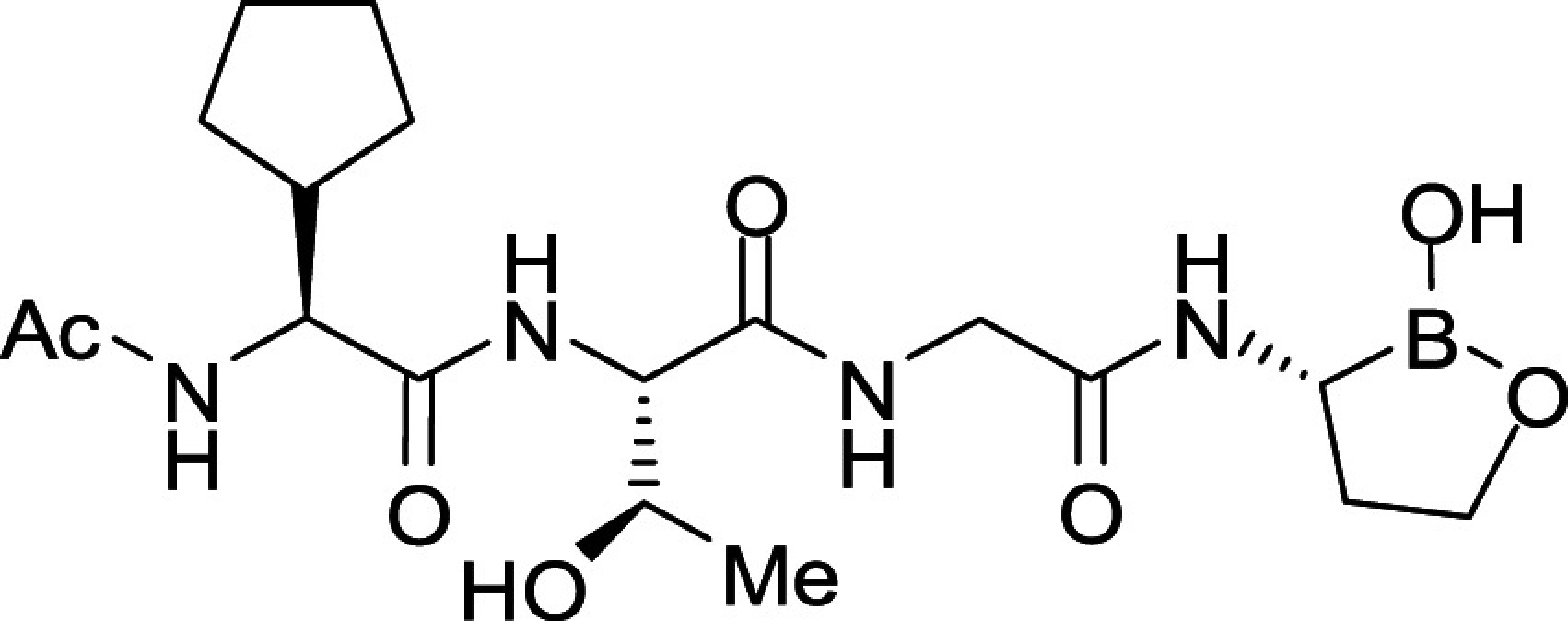	4.6 ± 0.1	15.0 ± 3.6
6	**3f**	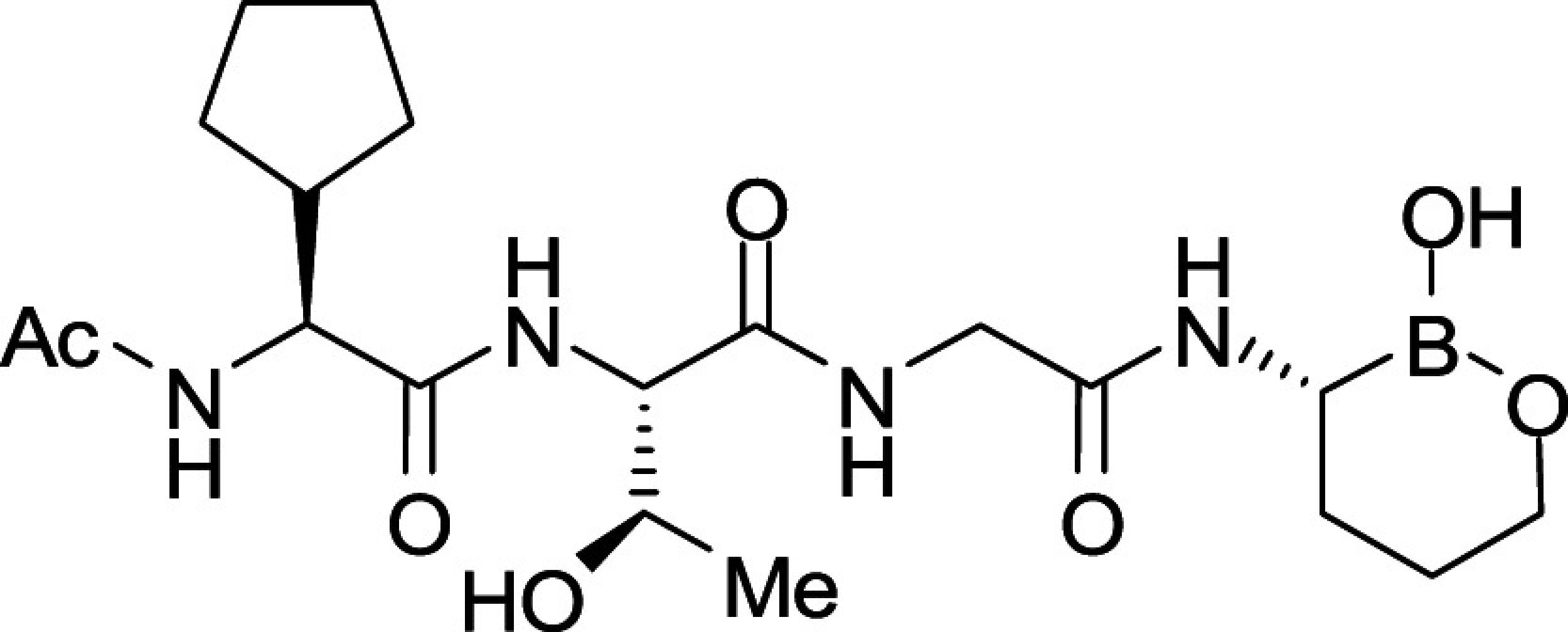	204.2 ± 7.5	N.D.
7	**3g**	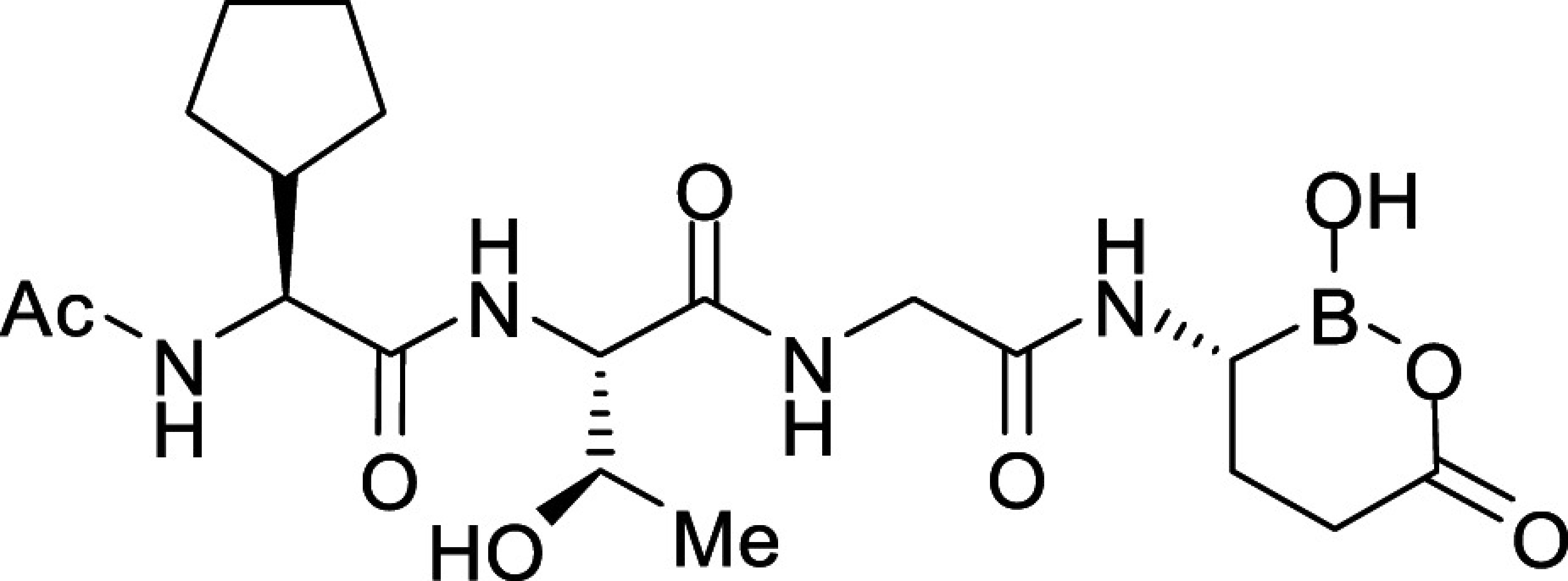	18.7 ± 1.3	N.D.
8	**3h**	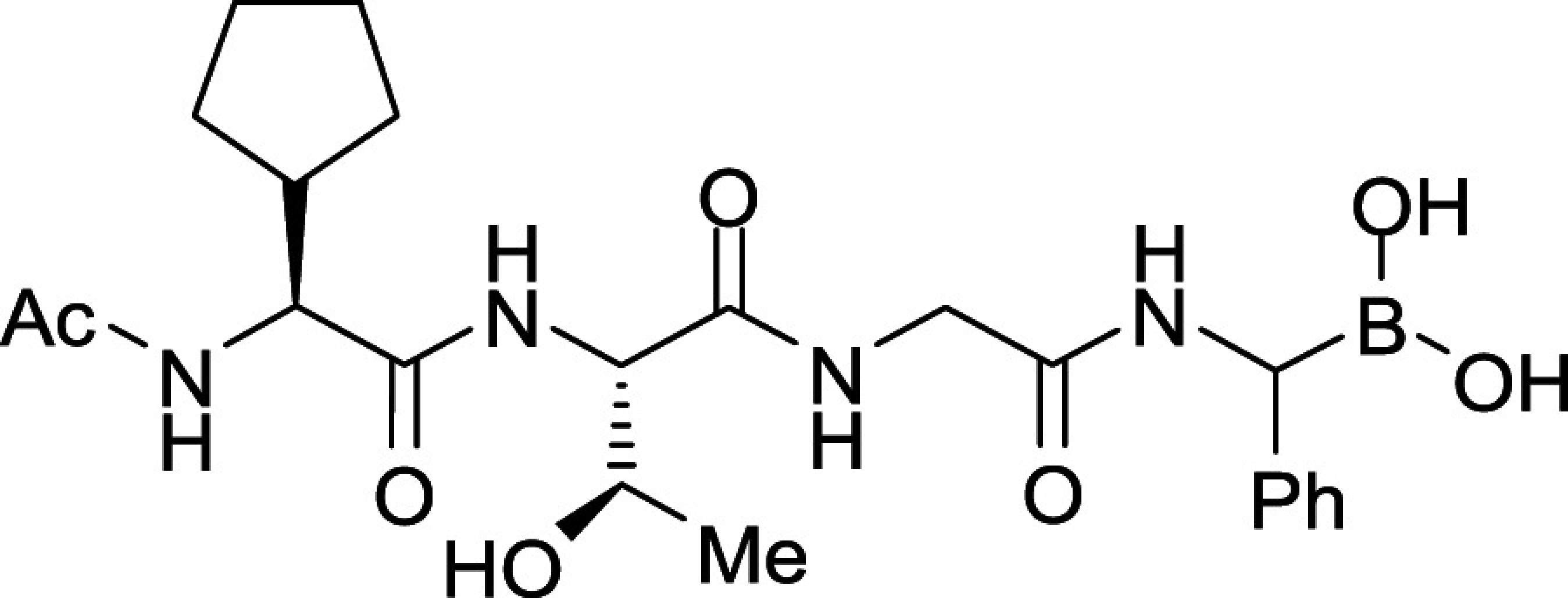	112.0 ± 2.3	N.D.
9	**3i**	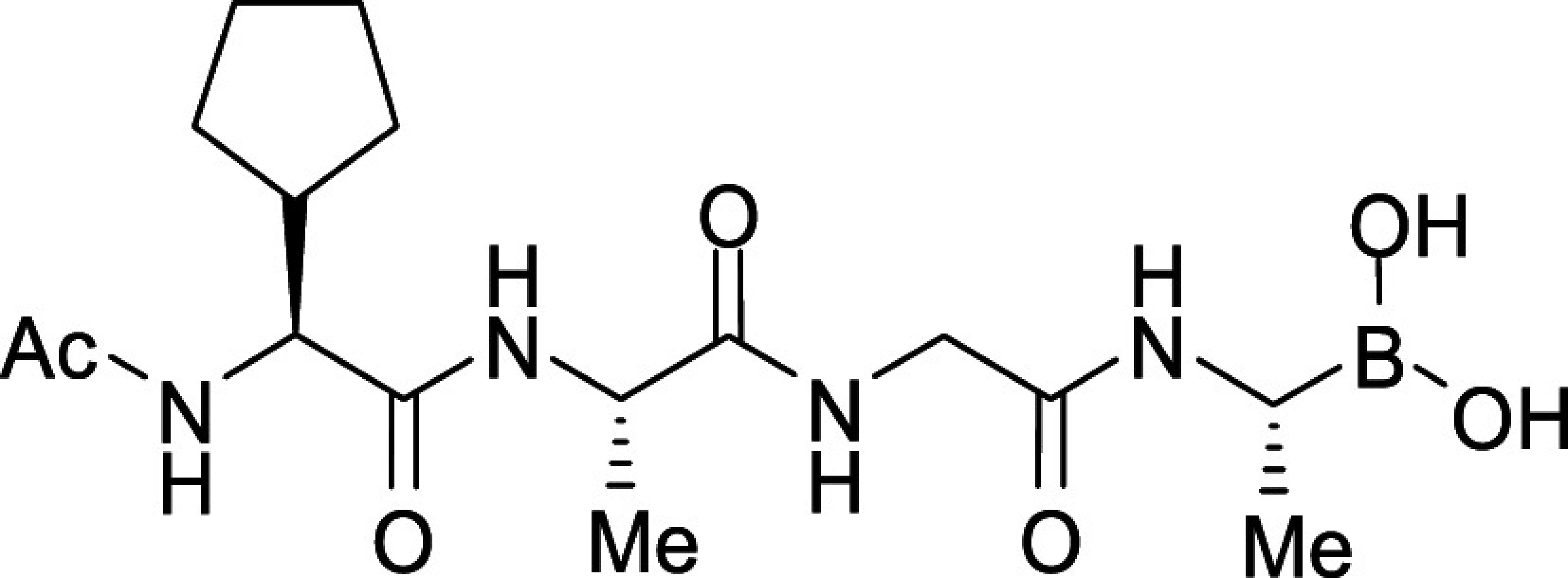	7.8 ± 0.3	0.34 ± 0.08
10	**3j**	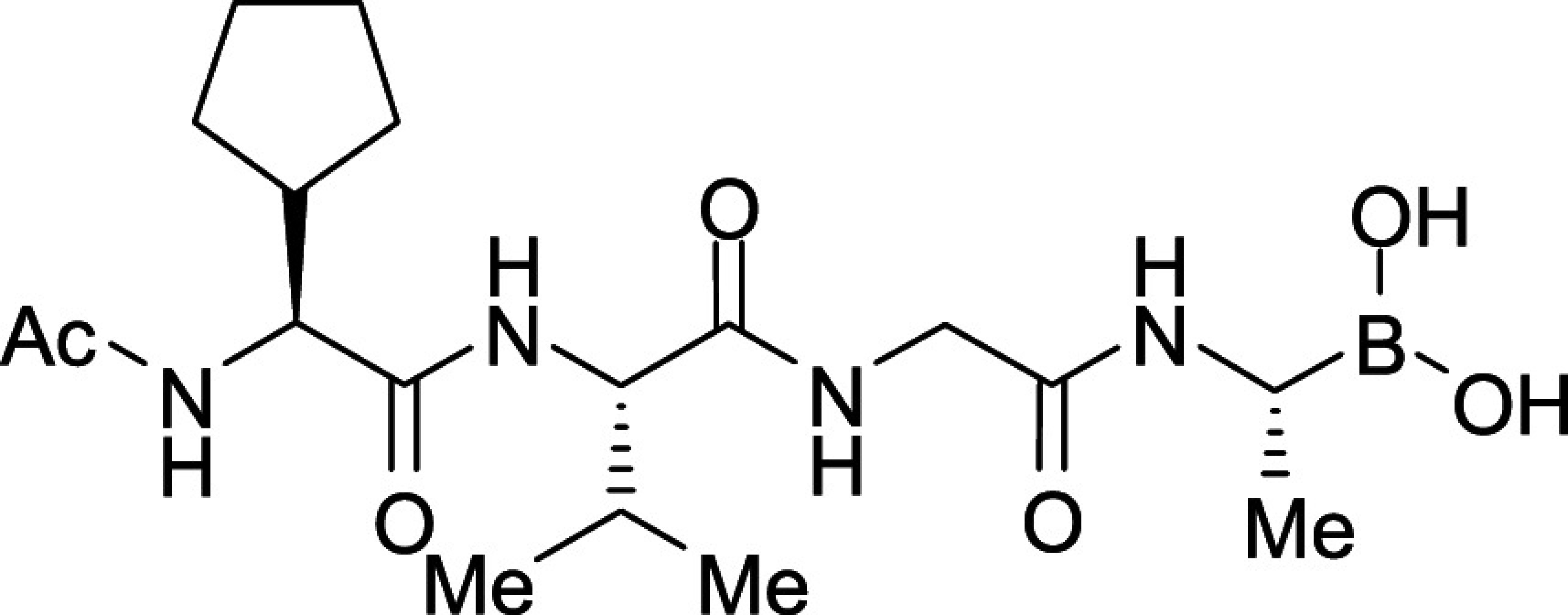	5.7 ± 0.1	0.26 ± 0.06

* IC_50_ values were determined by quantifying inhibition of rPfSUB1-mediated proteolytic cleavage of a fluorogenic peptide substrate. Values are mean averages from at least three independent measurements ± SD.

^†^ EC_50_ values were obtained by quantifying inhibition of *P. falciparum* growth in vitro over a period of 96 h (two erythrocytic growth cycles) using the DNA-binding fluorescent dye SYBR Green I to measure parasite replication (22). Values are mean averages from at least three independent measurements ± SD.

N.D., not determined.

To examine the importance of the stereochemistry of the aminoboronic acid substructure at the P1 position, the PfSUB1 inhibitory potency of boronic acid epimer **3c** was examined (Table 1). We found that **3c** was significantly less potent than **3b** (Table 1), indicating the requirement for a chiral center configuration matching that of the L-amino acid in native substrates of SUB1. We therefore maintained this stereochemistry in all subsequent boronic acid analogs.

Further work focused on enhancing the potency of the compound **3b** structural template. Removal of the methyl side chain at the P1 subsite (compound **3d**) reduced potency by eightfold. On the other hand, attempts to improve potency by exploring extended alkyl or phenyl substituents at the P1 subsite (compounds **3e**, **3f**, **3g**, and **3h**) met with only limited success, although compound **3e** bearing a hydroxyethyl substituent displayed ∼twofold increased potency over compound **3b**. This appears to contradict earlier substrate specificity studies, which indicated a preference for the S1 subpocket of PfSUB1 to accommodate polar sidechains (13). The observation may be explained by a preference of nucleophilic P1 side-chain residues to form cyclic boronic acids, preventing the polar hydroxyl group from engaging in interactions with the enzyme.

Conditional gene disruption experiments have shown that PfSUB1 is essential for asexual blood-stage parasite survival in vitro (12). To assess the capacity of the compounds to interfere with parasite replication, we used an in vitro growth assay, which exploits the DNA-binding fluorescent dye SYBR Green I to measure parasite proliferation in human RBCs (which do not possess a nucleus) (22). This showed that while all the compounds inhibited parasite replication, with EC_50_ values as low as 1.8 μM, there was a poor correlation between growth inhibition and the PfSUB1 enzyme-inhibitory potency of the compounds (Table 1). In particular, the most potent inhibitor of PfSUB1 enzymatic activity, compound **3e**, was more than sixfold less growth inhibitory than compound **3b**. We reasoned that the polar nature of **3e** likely limits its membrane permeability. It was concluded that this set of compounds suffered from poor access to PfSUB1 within the intracellular parasite, probably due to low cellular permeability.

### P3 Modification Results in Peptidic Boronic Acids with Submicromolar Parasite Growth Inhibitory Activity.

**Fig. 2.**
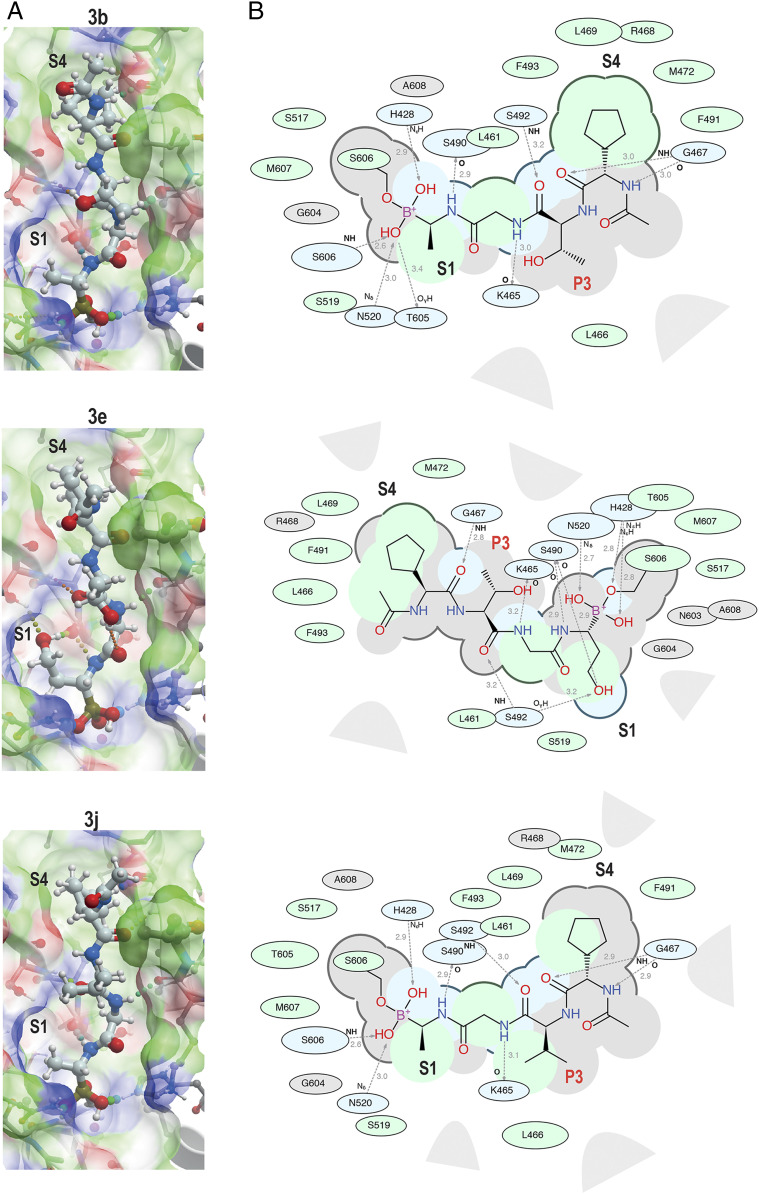
Substrate-based development of peptidic boronic acid inhibitors of PfSUB1. (*A*) ICM-Pro best docking poses for the PfSUB1-inhibitory compounds **3b** (ICM-Pro score −27), **3e** (ICM-Pro score −32), and **3j** (ICM-Pro score −34) in the active site of PfSUB1 (4LVN). The inhibitors are represented as colored balls and sticks. Hydrogen atoms are shown, while hydrogen bond interactions are indicated (dotted lines). The enzyme molecular surface is shown in transparent mode (green, hydrophobic; blue, hydrogen bond donor; and red, hydrogen bond acceptor). ICM docking score values below −32 (unitless) are considered good docking scores. (*B*) Corresponding two-dimensional interaction diagrams, with green shading for hydrophobic regions, blue shading for hydrogen bond acceptors, gray dashed arrows for hydrogen bonds (including length in Å), broken thick lines around ligand shape for accessible surfaces, and gray parabolas for large accessible surface areas. Interacting PfSUB1 residues are labeled and enclosed in oval shapes, the size of which varies depending on the degree of residue contribution. In all cases, the ligand boron atom is shown covalently bound to the active-site Ser606 Oγ through a boron ester bond, and positions of the S1 and S4 PfSUB1 active-site pockets are indicated. The P3 position is annotated in red in *B*.

To seek insights into how the most potent PfSUB1-inhibitory compounds of this first boronic acid series might be accommodated into the PfSUB1 active-site groove, we took advantage of the X-ray crystal structure of PfSUB1 (16) to perform in silico molecular docking of compounds (**3b** and **3e**; Fig. 2). We examined the bound molecules in a docked pose in which the boron atom was involved in a tetrahedrally coordinated intermediate involving the catalytic His428 (Nε_2_H) and the oxyanion hole partner N520 (N_δ_) and engaged in a covalent bond with the Oγ of the catalytic Ser606 of PfSUB1. This showed conservation of the substrate-enzyme canonical H-bond pattern, with the inhibitor peptidic backbone interacting with PfSUB1 residues Gly467 (NH), Ser490 (NH), and Ser492 (NH). For both inhibitors, the P4 cyclopentane was nicely accommodated into the S4 pocket (shaded green for hydrophobicity and delimited by a thick solid line to indicate optimal steric filling in Fig. 2*B*). The inhibitor **3b** P1 Ala side chain did not fill the S1 pocket entirely (indicated by the absence of a solid line at the bottom of the S1 pocket) but occupied the hydrophobic part of the pocket. In the case of inhibitor **3e**, docked in the form of an acyclic boronic acid, the P1 hydroxyethyl extension filled the S1 pocket fully and was stabilized by hydrogen bonding with Ser490 Oγ and Ser492 Oγ at the bottom of the pocket. Despite this, little improvement in potency of inhibitor **3e** over inhibitor **3a** was observed, which as mentioned above we suspect is likely explained by compound **3e** adopting the preferential cyclic form of the boronic acid.

Consistent with the X-ray crystal structure of PfSUB1, which includes its propeptide bound into the active-site groove of the catalytic domain in a substrate-like manner, the P3 Thr side chain of the docked compounds **3b** and **3e** was observed to extend into solvent, with no significant contacts with the molecular surface of the PfSUB1 catalytic domain. Interestingly, however, in both docking poses we noticed potential for modifying and/or extending the P3 side chain (openness depicted in gray in the two-dimensional diagram) in order to promote hydrophobic interactions with the side-chain carbon atoms of Lys465 and Leu466 that line the side of the S3 pocket. In silico replacement of the P3 Thr with Val supported this, revealing potential hydrophobic interactions between the Val P3 side chain and the side chains of Leu466 and Lys465 (Fig. 2*B*).

In accord with this, we prepared compounds **3i** and **3j** in which the P3 Thr of compound **3b** was replaced, respectively, with an Ala and Val side chain (Table 1). The new compounds showed slightly improved (**3i;** IC_50_ ∼7.8 nM) or nearly twofold improved (**3j**; IC_50_ ∼5.7 nM) potency relative to compound **3b** in the in vitro PfSUB1 enzyme assay. Significantly, compounds **3i** and **3j** displayed ∼10-fold improved growth inhibitory potency in the SYBR Green I parasite growth assay, likely due to their increased lipophilicity, which was expected to confer better membrane permeability.

### Inhibition of *P*. *falciparum* Egress by Selective Peptidic Boronic Acids that Access PfSUB1 in Intracellular Parasites.

**Fig. 3.**
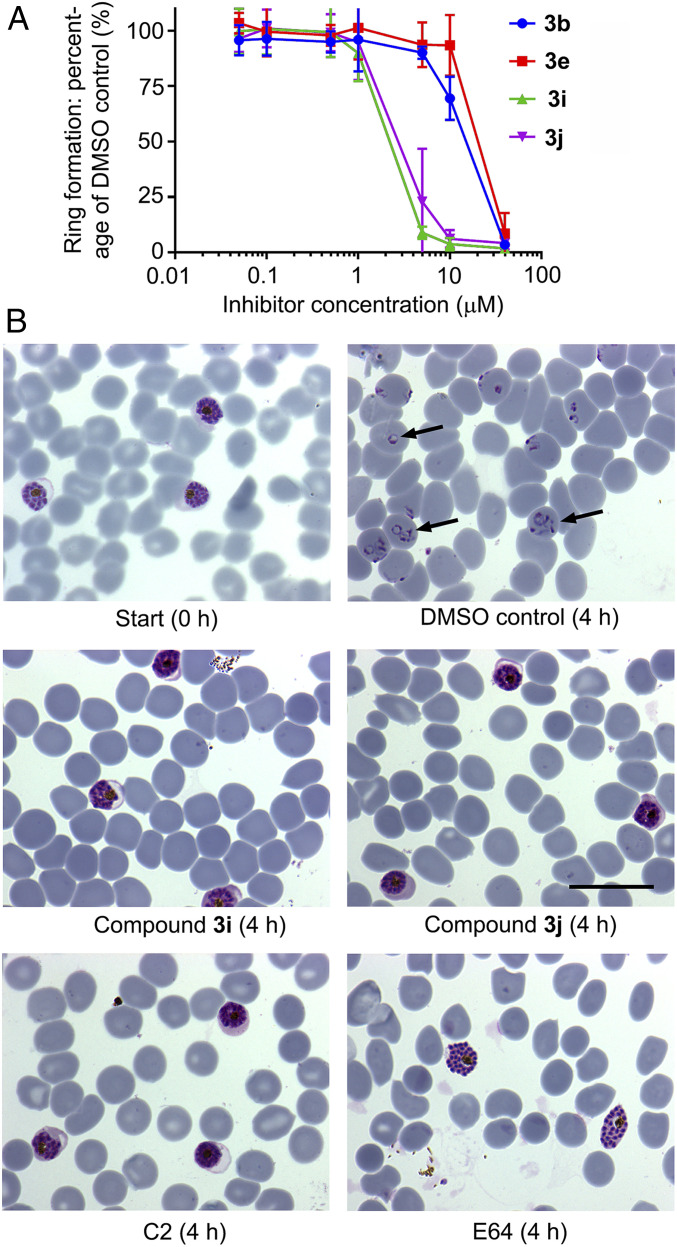
Peptidic boronic acid PfSUB1 inhibitors prevent *P*. *falciparum* egress. (*A*) Dose–response curves showing ring formation following incubation of highly mature 3D7 schizonts with RBCs for 4 h in the presence of the indicated compounds. Values are means of three independent experiments. Calculated EC_50_ values were as follows: compound **3b**, 12.7 ± 0.8 μM; **3e**, 15.7 ± 2.5 μM; **3i**, 2.0 ± 0.1 μM; and **3j** 2.5 ± 0.3 μM. Error bars, SD. (*B*) Light micrographs of Giemsa-stained thin films prepared from selected cultures similar to those described in *A*, sampled prior to start or following the 4 h incubation step. Extensive ring formation is evident in the control culture (examples indicated by arrows). In contrast, cultures containing compounds **3i** or **3j** (10 μM) show arrest of unruptured schizonts with no ring formation. Note that the phenotype of the **3i**- or **3j**-arrested schizonts is similar to that of C2-treated parasites but distinct from those arrested by the cysteine protease inhibitor E64, where PVM rupture occurs allowing release of the enclosed merozoites into the RBC cytosol. (Scale bar, 20 μm.)

To determine their mode of action, the four most potent growth inhibitory compounds were next evaluated using very short-term cell-based assays focused on the narrow window within the asexual blood-stage lifecycle during which the parasite undergoes egress from host RBCs and invasion into fresh cells. For this, *P*. *falciparum* cultures containing synchronous, highly mature schizonts were supplemented with compounds **3b**, **3e**, **3i**, and **3j** at a range of dilutions, then allowed to undergo egress and invasion for just 4 h in the continued presence of the compounds, before assessing formation of newly invaded “ring” stage parasites by flow cytometry. This confirmed a dose-dependent inhibitory effect on the transition from schizont to ring stage, with the relatively lipophilic compounds **3i** and **3j** displaying similar EC_50_ values that were significantly lower than those of **3b** and **3e** (Fig. 3*A*). Microscopic examination of the cultures revealed schizonts arrested by compounds **3i** and **3j**, confirming inhibition of schizont rupture. Importantly, the arrested schizonts were morphologically indistinguishable from those arrested by the reversible PKG inhibitor (4-[7-[(dimethylamino)methyl]-2-(4-fluorphenyl)imidazo[1,2-α]pyridine-3-yl]pyrimidin-2-amine (C2)), appearing as segmented forms trapped within an apparently intact PVM and RBC membrane (Fig. 3*B*). This egress-arrest phenotype is similar to that obtained by genetic disruption of PfSUB1 and was clearly different from that following arrest by the cysteine protease inhibitor E64 (Fig. 3*B*), which does not inhibit PfSUB1 directly but which blocks a cysteine protease-dependent step in egress following SUB1 discharge and PVM rupture (12).

**Fig. 4.**
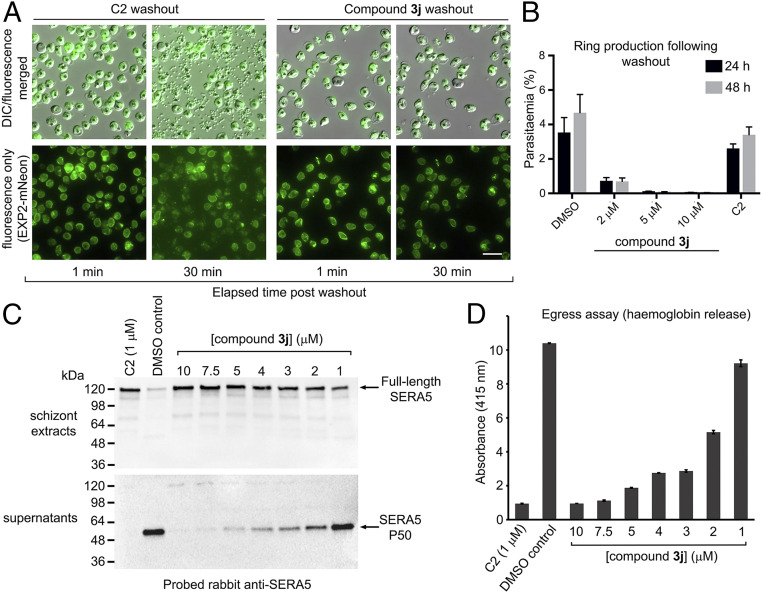
Washout experiments show that peptidic boronic acid 3j is a membrane-permeable inhibitor of PfSUB1 and *P*. *falciparum* egress. (*A*) Stills from time-lapse video microscopic monitoring of purified schizonts following washout of the indicated treatments. The parasites express an mNeonGreen fusion of the PVM protein EXP2. Washout of drug from schizonts arrested with the reversible PKG inhibitor C2 (1 μM) resulted in normal egress, initiating at ∼6.5 min following washout. In contrast, no egress occurred over the course of 30 min following washout of parasites treated with saturating amounts (10 μM) of compound **3j**, and there was no discernible change in shape or integrity of the PVM (although slight time-dependent photobleaching of the fluorescence signal is evident). Identical results were obtained in four independent experiments. (Scale bar, 20 μm.) Also reference Movies S1 and S2. (*B*) Ring formation following incubation with fresh RBCs of schizonts pretreated with vehicle only (DMSO), C2 (1 μM), or compound **3j**. Drugs were washed away before addition of RBCs. Ring production was assessed at 24 h. Parasitaemia was also assessed at 48 h to ensure that the rings detectable at 24 h were viable. No rings were produced by the 10 μM **3j** pretreated schizonts, whereas schizonts pretreated with the reversible PKG inhibitor C2 produced rings efficiently. Results shown are from three independent experiments in different batches of blood. Error bars, ± SD. (*C*) Western blot analysis of mature schizonts and culture supernatants thereof following pretreatment for 4 h with compound **3j** at the indicated concentrations, then washout before analysis of egress. The parasite PV protein SERA5, which is proteolytically converted to the P50 fragment through the action of PfSUB1, appeared in the supernatants of control schizonts (which underwent egress) but remained intracellular in its intact, full-length form at higher concentrations of **3j**. As expected, SERA5 processing was also blocked by C2 (positive control). (*D*) Quantitation of hemoglobin release into culture supernatants (an indicator of the extent of egress) in the assay analyzed in *C*. Error bars, SD. Data shown are typical of four independent experiments.

Examination of the inhibitory activity of compound **3j** against the mammalian trypsin-family serine proteases trypsin, chymotrypsin, and elastase revealed a high degree of selectivity for PfSUB1 (>>100-fold; *SI Appendix*, Figs. S1–S3), encouraging us to focus subsequent work on this compound. To directly visualize the inhibitory effects of compound **3j** on parasite egress and to examine the reversibility of inhibition, we used live time-lapse video microscopy to observe the behavior of schizonts exposed to the compound for just 1 h immediately prior to egress. For this, we used a transgenic parasite line expressing a PVM protein (EXP2) fused with the green fluorescent protein mNeon Green, facilitating real-time visualization of PVM integrity as previously reported by Glushakova and colleagues (23). As shown in Fig. 4*A* and Movie S1, this clearly demonstrated significant inhibition of PVM rupture and egress in parasites treated with **3j**, with no signs of egress even 30 min following washout of the compound. Importantly, **3j**-treated parasites remained viable, as shown by their continued capacity to incorporate the vital mitochondrial dye MitoTracker Red CMXRos (24) (*SI Appendix*, Fig. S4), but showed no signs of the PVM rounding and other morphological changes that typically precede egress (23, 25), indicating a complete and selective block in the egress pathway. These egress-associated transitions were also absent from PfSUB1-null parasites (12), indicating that the effects of **3j** closely mimic genetic disruption of PfSUB1. Further extended incubation of the treated, washed schizonts with fresh RBCs resulted in only very limited appearance of new ring stage parasites (Fig. 4*B*). This confirmed that even short-term treatment with compound **3j** could dramatically impede parasite escape from the host RBC and that the egress inhibition over these timescales was effectively irreversible.

These results suggested that compound **3j** can access and inhibit PfSUB1 in an intracellular location (i.e., within the intraerythrocytic parasite, or in the PV, or both). To seek unambiguous confirmation that PfSUB1 is the intracellular target of compound **3j**, we examined the effects of the compound on the PfSUB1-mediated proteolytic processing of the established endogenous PfSUB1 substrate SERA5, an abundant parasite PV protein that only becomes accessible to cleavage upon discharge of PfSUB1 into the PV in the minutes leading up to egress and is then released in a processed form into culture supernatants (6, 11, 12). As shown in Fig. 4*C*, treatment with compound **3j** reproducibly prevented proteolytic processing and release of SERA5 into culture supernatants in a dose-dependent manner, even following compound washout. Crucially, at higher concentrations of the drug where egress and release of processed SERA5 was completely blocked, no intracellular processing of SERA5 was evident in the intact egress-arrested schizonts. Quantitation of egress in these same washout assays by measuring release of residual hemoglobin from the rupturing schizonts showed an EC_50_ of ∼2 μM (Fig. 4*D*), similar to the EC_50_ value for new ring generation previously determined by flow cytometry (Fig. 3*A*). It was concluded that compound **3j** prevents egress and parasite proliferation through direct inhibition of intracellular PfSUB1.

### An Optimized Membrane-Penetrant Peptidic Boronic Acid Displays Time-Dependent, Slowly Reversible Binding Kinetics to PfSUB1.

Boronic acids form reversible covalent bonds with serine and threonine proteases (26). Inhibition is generally time dependent, and the covalent nature of the binding can result in relatively long target occupancy times despite the reversibility of the bond. That this might be the case with compound **3j** binding to PfSUB1 was initially suggested by our washout experiments (Fig. 4), which showed that egress inhibition by compound **3j** following washout was much longer lasting than the rapidly reversible egress inhibition mediated by C2. To analyze the kinetic characteristics of the interaction between **3j** and rPfSUB1, we used progress curve analysis to continuously monitor rPfSUB1-mediated cleavage of a fluorogenic substrate in the presence of a range of concentrations of **3j**. As shown in *SI Appendix*, Fig. S5, under conditions where substrate cleavage in the absence of inhibitor (control reaction) displayed a linear relationship with time, indicating negligible substrate depletion, progress curves in the presence of compound **3j** became progressively nonlinear, characteristic of slow-binding (time-dependent) inhibition. Under such conditions, fit of the progress curves by nonlinear regression to Eq. 1 (see *Materials and Methods*) allows determination of *k*_*obs*_, the pseudo first-order rate constant for onset of inhibition. The *k*_*obs*_ is effectively a composite of the on and off rates, so least linear squares regression of the calculated *k*_*obs*_ values against inhibitor concentration allows determination of values of the pseudo first-order dissociation rate constant *k*_*off*_ and the second-order association rate constant *k*_*on*_ for the inhibitor-rPfSUB1 interaction, based respectively on values from the *y*-intercept and slope. The *y*-intercept value corresponds to a *k*_*off*_ of 3.7 × 10^−4^ s^−1^, which equates to a *t*_1/2_^*off*^ (bound half-life) of ∼31 min. The calculated *k*_*on*_ value was 3.6 × 10^5^ M^−1^.s^−1^, allowing calculation of an apparent equilibrium inhibition constant *K*_I_^kin^ (*k*_*off*_/*k*_*on*_) of 1.0 nM. It was concluded that compound **3j** is a potent, slowly reversible inhibitor of PfSUB1, completely consistent with the washout data.

## Discussion

**Fig. 5.**
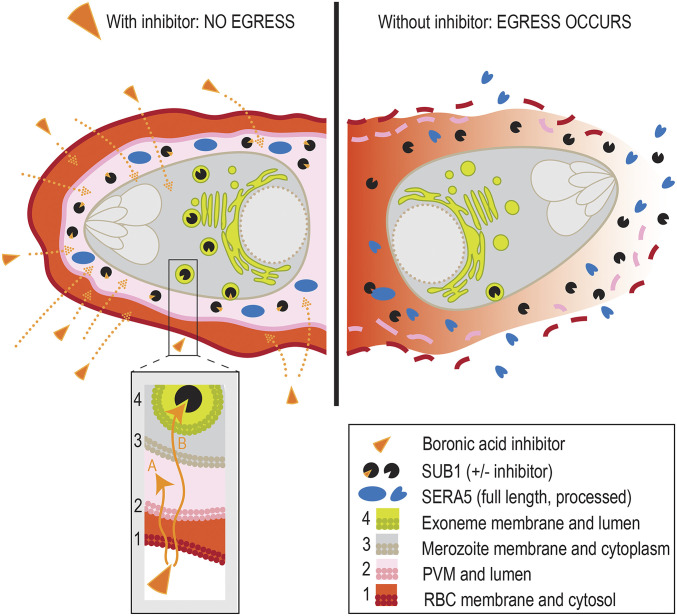
Schematic indicating the requirement for inhibitors of SUB1 to cross at least two and up to four membranes to access and inactivate the enzyme in intraerythrocytic parasites. Inhibition likely occurs either in the PV (route A), or the exonemes (route B), or both.

Prior to parasite egress from the confines of its host RBC, SUB1 is stored in membrane-bound merozoite secretory organelles called exonemes before its discharge into the PV lumen minutes before egress to encounter its endogenous substrates. As a result, in order to gain access to the intracellular enzyme prior to substrate cleavage, exogenously applied inhibitory compounds likely need to cross at least two and as many as four distinct biological membranes: the RBC membrane, the PVM, the parasite plasma membrane, and the exoneme membrane (Fig. 5). This poses particular challenges for the design of substrate-based inhibitors. In the case of covalent modifying compounds, such as those described here, access to the exoneme-resident enzyme could potentially allow inactivation of the stored SUB1 long before its PKG-regulated discharge into the PV. In this work, we did not determine the intracellular site of PfSUB1 inhibition, so we cannot state whether inhibition took place within the PV, or the exonemes, or both. Regardless, by gradual optimization of the structure and lipophilicity of our compounds we have now successfully developed potent PfSUB1-inhibitory compounds that can functionally inactivate PfSUB1 within intact, parasite-infected RBCs and block egress.

Our conclusion that the intracellular inhibition of PfSUB1 mediated by compound **3j** is directly and causally responsible for the observed block in egress is most clearly supported by the phenotype of the arrested schizonts, which was indistinguishable from that resulting from conditional genetic disruption of the *PfSUB1* (12) or *PKG* gene (27), or following treatment with the PKG inhibitor C2, with no signs of the morphological changes that typically precede egress such as PVM rounding or PV rupture. We cannot rule out the possibility of effects on other parasite enzymes at the concentrations used to obtain complete egress inhibition, even in the short-term assays designed to focus on the short window of the parasite life cycle over which egress occurs. However, we consider off-target effects unlikely given that no other parasite serine protease has been implicated in egress; the only two other subtilisin-like enzymes expressed in the parasite, SUB2 and SUB3, are respectively dispensable for egress (28) or nonessential in blood stages (29). The ∼10-fold higher potency of **3j** in the long-term SYBR Green–based parasite growth assay (EC_50_ 0.26 μM; Table 1) may be a result of the fact that this assay captures the combined effects of egress inhibition over the course of two erythrocytic cycles in the continuous presence of the drug, although again off-target effects cannot be formally ruled out. We anticipate that further optimization of the PfSUB1-inhibitory potency and membrane permeability of **3j** is highly feasible. Work is already underway to determine the atomic structure of the **3j**-PfSUB1 complex to facilitate structure-based inhibitor improvement.

Peptidic boronic acids have long-established therapeutic potential, as best exemplified by the widespread clinical use for multiple myeloma of the proteasome inhibitors bortezomib (Velcade) and ixazomib, the latter of which is orally bioavailable in its citric acid form, Ninlaro. The clinical success of these compounds is in part due to the long drug target residence times that can be obtained with slowly reversible covalent inhibitors. Target binding by boronic acid protease inhibitors is generally time dependent, perhaps further explaining the differences in potency we observed between the long-term and short-term cellular assays with compounds **3i** and **3j**. However, our washout experiments with compound **3j** suggest that, once bound to PfSUB1 in the exonemes and/or PV, it takes at least 30 min for the level of target engagement to fall below a threshold that allows successful egress. Examination of the capacity of schizonts treated with saturating levels of **3j** to productively egress and form new rings following compound washout showed that the egress block under these conditions was effectively irreversible. An alternative explanation for this apparently irreversible inhibition of egress by **3j** is that the inhibitor is not easily washed out due to its accumulation in the parasite (or infected RBC) at high concentrations. We cannot formally rule out this possibility. However, our ex vivo kinetic analysis of the inhibition of rPfSUB1 by compound **3j** fully supports a slow off rate, with an estimated bound half-life (*t*_1/2_^off^) of ∼30 min, very similar to that of the interaction between bortezomib and the β5 chymotrypsin-like subunit of the human proteasome (30–32). Even if cellular accumulation does contribute to the prolonged egress inhibition exerted by **3j**, this is only likely to favor efficacy; indeed, intracellular accumulation is an important component of the mode of action of the important antimalarial 4-aminoquinoline chloroquine (33). While peptide-based drug development can present challenges for in vivo applications due to metabolic instability, covalent compounds can be effective even with relatively short plasma half-lives, since target residence time can be longer than plasma half-life. Peptidyl boronic acids can anyway have excellent pharmacodynamic properties; for example, the terminal half-life of ixazomib is ∼9.5 d (a fact that allows weekly dosing of patients for treatment of multiple myeloma; ref. 34), which is nearly five times the duration of the *P*. *falciparum* asexual blood-stage lifecycle.

SUB1 has an unusual substrate specificity, which differs subtly between different *Plasmodium* species, suggesting that the enzyme and its multiple cognate parasite substrates have coevolved to ensure optimal cleavage efficiency (13). As a result, inhibitors of PfSUB1 are unlikely to show similar potency against SUB1 orthologs from rodent malaria parasite species such as *Plasmodium berghei*, making these parasite species unsuitable as model systems for assessing the in vivo efficacy of our compounds. Importantly, SUB1 also lacks structural resemblance to any known human serine protease (16), reducing the likelihood of substrate-based SUB1 inhibitors displaying toxicity due to off-target activity against host enzymes. In support of this, we found here that **3j** is only poorly potent against the mammalian serine proteases examined. Toxicity can be especially problematic where long-term or life-long treatment regimens are required due to chronic infection (e.g., with HIV). However, malaria is an acute disease, and long-term therapeutic regimens are rare; indeed, current standard treatments for uncomplicated falciparum malaria are just 3 d long. Since SUB1 plays an essential role in the development and release of exoerythrocytic (liver-stage) merozoites that initiate blood-stage infection (14, 15), medicines based on SUB1 inhibitors have prophylactic as well as therapeutic potential. Optimized SUB1 inhibitors could also potentially be combined with inhibitors of other essential enzymes in the egress pathway, including PKG (35–37) and the SUB1 aspartic protease maturase plasmepsin X (38–41). Such combinations could yield additive or synergistic enhancement of potency and decrease opportunities to select for drug resistance.

In conclusion, we have produced substrate-based peptidic boronic acids that block asexual blood-stage *P*. *falciparum* proliferation through direct, effectively irreversible inhibition of intracellular PfSUB1. Further investigation of the pharmacokinetic properties and structure-based improvement of these compounds has the potential to generate compounds suitable for preclinical trials in animal models of malaria.

## Materials and Methods

### *P*. *falciparum* Maintenance and Manipulation.

Asexual blood stages of *P*. *falciparum* (clones 3D7 and B11) (42) were routinely maintained at 37 °C in human erythrocytes at 1 to 4% hematocrit in RPMI 1640 containing Albumax II (Thermo Scientific) supplemented with 2 mM L-glutamine in a low oxygen atmosphere using standard procedures (43). Human blood was obtained from anonymized donors through the UK National Blood and Transplant service and was used within 2 wk of receipt. No ethical approval is required for its use. For synchronization, mature schizont-stage parasites were isolated on cushions of 70% (volume[vol]/vol) Percoll (GE Healthcare) adjusted to isotonicity as described (43). Routine microscopic examination of parasite growth was performed by fixing air-dried thin blood films with 100% methanol before staining with 10% Giemsa stain (VWR international) in 6.7 mM phosphate buffer, pH 7.1.

Generation of the B11-EXP2-mNeonGreen line was achieved by fusing mNeonGreen to the endogenous C terminus of EXP2 using Cas9-mediated gene editing, following the methods of ref. 23. A pair of guide RNAs were designed targeting a region toward the 3′ end of the EXP2 locus (oligo 1: ATT​GAT​ATT​ATG​TAC​AGT​ATC​TGA, oligo 2: AAA​CTC​AGA​TAC​TGT​ACA​TAA​TAT) (Sigma). This oligonucleotide pair was annealed with T4 PNK ligase (New England Biolabs) and ligated with T4 ligase (New England Biolabs) into the U6 cassette of the Cas9 vector (pDC2-Cas9_U6-hDHFR) previously digested with Bsb1-HF (New England Biolabs). The plasmid was propagated under ampicillin selection in *Escherichia coli* and sequenced to check for correct incorporation of the guide (Genewiz). The resulting plasmid was cotransfected into B11 schizonts along with the repair plasmid pyPM2GT-EXP2-mNG (a kind gift of Josh Beck, Iowa State University, Ames, IA), linearized with AflII (New England Biolabs). Drug selection for integration was carried out with 2.5 nM WR99210 (Sigma-Aldrich) from 24 h posttransfection, and clonal lines of the resulting B11-EXP2-mNeonGreen line were obtained by limiting dilution cloning and treatment with 1 μM Ancotil (5-fluorocytosine) before use.

### Parasite Growth, Egress, and Invasion Assays.

The impact of the peptidic boronic acids on replication of asexual blood-stage *P*. *falciparum* (clone 3D7) was assessed using a SYBR Green I assay, essentially as described by Smilkstein et al. (22). Briefly, test compounds (dissolved in dimethyl sulfoxide [DMSO] at concentrations ranging from 4 mM to 5 μM) were added in triplicate to wells of flat bottomed, 96-well microtitre plates (1 μL per well). Wells were then supplemented with 100 μL per well of a *P*. *falciparum* parasite culture at 0.1% parasitaemia, 1% hematocrit. Each assay plate also included DMSO-only control wells (1% vol/vol), as well as additional control wells containing uninfected RBCs only. Plates were incubated in sealed, humidified gassed chambers at 37 °C for 96 h to allow the parasites to undergo two entire cycles of erythrocytic growth. Wells were then supplemented with 100 μL 1:5,000 dilution of stock SYBR Green I (Life Technologies, catalog no. S7563) diluted in 20 mM Tris HCl pH 7.5, 5 mM EDTA, 0.008% (weight/vol) saponin, 0.08% (vol/vol) Triton ×100. Plates were agitated to mix, incubated for a further 1 h in the dark at room temperature, then 150 μL samples from each well transferred to a fresh white microwell plate and fluorescence quantified using a SpectraMax M5e plate reader and SoftMax Pro-6.3 software (Ex 485 nm/Em 530 nm). IC_50_ values were determined from dose–response curves obtained after subtracting background fluorescence values (obtained from the RBC-only wells) from all experimental readings.

Short-term egress, invasion, and washout assays were performed essentially as described previously (27, 28, 42). Briefly, highly synchronous mature Percoll-enriched schizonts with or without added fresh RBCs (∼5% parasitaemia final) were incubated with compounds under test or vehicle only (DMSO, 1% vol/vol). For washout assays, schizonts were treated with C2 or various concentrations of inhibitor **3j** for 1 to 4 h, then washed extensively (at least four times) prior to addition to fresh RBCs where required. After incubation at 37 °C for just 1 to 4 h (or overnight for invasion assays) to allow schizont rupture, cells were pelleted. Clarified culture supernatants were assessed for extent of hemoglobin release (a measure of schizont rupture) by absorption spectroscopy at 415 nm as described previously (28) or analyzed by Western blot using antibodies against SERA5 (7, 11). To quantify generation of new rings, samples of the cultures were fixed with 4% paraformaldehyde/0.02% glutaraldehyde and stained with SYBR Green I (Life Technologies) and then analyzed by flow cytometry on a BD FACSVerse using BD FACSuite software. Data were analyzed using FlowJo software. All cultures were also routinely analyzed by microscopic examination of Giemsa-stained thin films to visually assess parasite morphology.

### Time-Lapse and Live Fluorescence Microscopy.

Viewing chambers for live parasite microscopic examination were constructed as previously described (7). All images were recorded on a Nikon Eclipse Ni light microscope fitted with a Hamamatsu C11440 digital camera and Nikon N Plan Apo λ 63×/1.45NA oil immersion objective. For time-lapse video microscopy, differential interference contrast (DIC) images were taken at 10 s intervals over 30 min while fluorescence (mNeon Green) images were taken every 2 min to prevent bleaching. Time-lapse videos were analyzed and annotated using Fiji (44). For viability staining using the vital mitochondrial dye MitoTracker Red CMXRos (ThermoFisher Scientific; stored as a 10 μM stock in DMSO), the dye was added (20 nM final concentration) to a suspension of schizonts pretreated for 1 h with either DMSO (control, 1% vol/vol) or compound **3j** (10 μM). The schizonts were incubated with the dye for 15 min at 37 °C, then washed twice, transferred to a viewing chamber, and observed immediately by dual DIC/fluorescence microscopy.

### Protease Inhibition Assays: IC_50_ Calculations and Progress Curve Kinetics.

Proteolytic activity of rPfSUB1 was quantified at room temperature by monitoring cleavage of the peptidic fluorogenic substrate SERA4st1F-6R12 (Ac-CKITAQDDEESC-OH possessing tetramethylrhodamine labeling of both cysteine residues) (13). Fluorescence of this peptide is quenched by rhodamine dimerization in the intact substrate but increases upon cleavage at the internal Q-D bond. Chymotrypsin-treated rPfSUB1 (expressed and purified as described previously (16) was stored at −80 °C as a 228 U/mL stock in 20 mM Tris–HCl pH 8.2, 150 mM NaCl, 10% glycerol, and diluted for use (1:500 or 1:600) in reaction buffer (20 mM Tris–HCl pH 8.2, 150 mM NaCl, 12 mM CaCl_2_, 25 mM CHAPS). Peptidic boronic acid inhibitors were dissolved in 100% DMSO at 10 or 20 mM, then further diluted in DMSO to generate stock solutions ranging from 500 to 0.01 μM and then used diluted 1:100 in the enzyme reactions. All reactions were performed in wells of white 96-well microwell plates (Nunc); 50 μL diluted rPfSUB1 was preincubated for 5 min with 1 μL each of the serially diluted boronic acid inhibitors, followed by addition of 50 μL substrate solution (0.1 μM final). Subsequent fluorescence increase was continuously monitored using a SpectraMax M5e plate reader and SoftMax Pro-6.3 software, with readings taken every 5 min for 60 min using excitation and emission values of 552 and 580 nm, respectively. Initial rates were calculated over the first 25 min of the assay, during which period progress curves were linear, and IC_50_ values were calculated with GraphPad Prism 8.0 using the nonlinear regression, [inhibitor] versus response, variable slope (four parameters). All experiments were performed in duplicate.

Details of the methodology used to evaluate the effects of compound **3j** on the proteolytic activity of the mammalian serine proteases trypsin, chymotrypsin, and elastase are provided in *SI Appendix*.

### Progress Curve Kinetic Analysis of Compound 3j.

Progress curves of SERA4st1F-6R12 cleavage by rPfSUB1 were acquired at seven concentrations of inhibitor compound **3j** over a period of 35 min, during which fluorescence increases in the absence of inhibitor were linear. The obtained progress curves (four independent replicates) were fit using GraphPad Prism 8.0 software to the following time-dependent inhibition equation:

$$\left[P\right]\;=\;\mathrm{Vs}*t\;+\;\left(\left(\mathrm{Vi}-\mathrm{Vs}\right)/k_{obs}\right)*\left(1-\exp\left(-k_{obs}*t\right)\right).$$ [1]

In the equation, Vi is the initial velocity, Vs is the final steady-state velocity, and *k*_*obs*_ reflects the observed pseudo first-order rate of inactivation. The obtained *k*_*obs*_ values were plotted against compound concentration using a linear least squares fit. All statistical analysis was carried out using GraphPad Prism 8.

### Covalent Docking.

Flexible covalent docking of peptidyl boronic acid compounds into the active site of PfSUB1 (Protein Data Bank: 4LVN) was performed using the Internal Coordinate Mechanics software (ICM-Pro) package version 3.9-1c/MacOSX (Molsoft LLC). The inhibitors were drawn using the ICM chemistry molecular editor and compiled into an sdf docking table. After adding hydrogen atoms to the structure, the C-terminal region of the SUB1 propeptide (P4 to P1 positions that occupy the SUB1 active site) was used to define boundaries within the enzyme active site for the docking procedure and then removed from the active site along with all water molecules prior to docking. The catalytic histidine (His428, Nε_2_) was protonated as part of the catalytic process, resulting from covalent binding of the boron atom ligand to the active Ser (Ser606 Oγ). The boronic acid covalent mechanism was selected from the ICM program reactions list. Potential energy maps of the SUB1 receptor pocket and docking preferences were set up using the program default parameters. Energy terms were based on the all-atom vacuum force field ECEPP/3, and conformational sampling was based on the biased probability Monte Carlo procedure (45). Four independent docking runs were performed per compound, with a length of simulation (thoroughness) varied from three to four and the selection of two docking poses. Ligands were ranked according to their ICM energetics (ICM score, unitless), which weighs the internal force-field energy of the ligand combined with other ligand-receptor energy parameters.

### Statistical Analysis.

All statistical analysis was carried out using GraphPad Prism 8.0.

## Supplementary Material

Supplementary File

Supplementary File

Supplementary File

## Data Availability

All study data are included in the article and/or supporting information.
