# Supplementary material for: Peptidic boronic acids are potent cell-permeable inhibitors of the malaria parasite egress serine protease SUB1
Source: Proc Natl Acad Sci U S A. 2021 May 11;118(20):e2022696118. doi: 10.1073/pnas.2022696118 (PMC8157947; doi:10.1073/pnas.2022696118)
Supplement: Supplementary File [file pnas.2022696118.sapp.pdf]

PNAS
www.pnas.org

Supplementary Information for

Peptidic boronic acids are potent cell-permeable inhibitors of the malaria parasite egress serine protease SUB1

Elina Lidumniece[a], Chrislaine Withers-Martinez[b], Fiona Hackett[b], Christine R. Collins[b], Abigail J. Perrin[b], Konstantinos Koussis[b], Claudine Bisson[c,d], Michael J. Blackman[b,e]*, Aigars Jirgensons[a]*.

[a]Latvian Institute of Organic Synthesis, Aizkraukles 21, Riga LV-1006, Latvia
[b]Malaria Biochemistry Laboratory, The Francis Crick Institute, 1 Midland Road, London NW1 1AT, UK
[c]Department of Biological Sciences, Institute of Structural and Molecular Biology, Birkbeck College, University of London, London WC1E 7HX, UK
[d]Centre for Ultrastructural Imaging, Kings College London, New Hunts House, Guys Campus, London SE1 1UL, UK
[e]Faculty of Infectious and Tropical Diseases, London School of Hygiene & Tropical Medicine, London WC1E 7HT, UK

*For correspondence. Email: mike.blackman@crick.ac.uk; aigars@osi.lv

This PDF file includes:

Supplementary text
Figures S1 to S5
Table S1
Legends for Movies S1 to S2
SI References

Other supplementary materials for this manuscript include the following:

Movies S1 to S2

Supplementary Information Text

1. Synthesis of peptidic boronic acids 3a-j (schemes)

Glycine esters **4a-c** were coupled with Fmoc-protected amino acids and the resulting dipeptides **5a-d** were *N*-deprotected, followed by the coupling with Fmoc-protected isoleucine or cyclopentylglycine. The dipeptides **6a-d** were *N*-deprotected and *N*-acetylated providing derivatives **7a-d** in which the ester group was hydrolysed to generate tripeptides **8a-d** (Scheme S1).

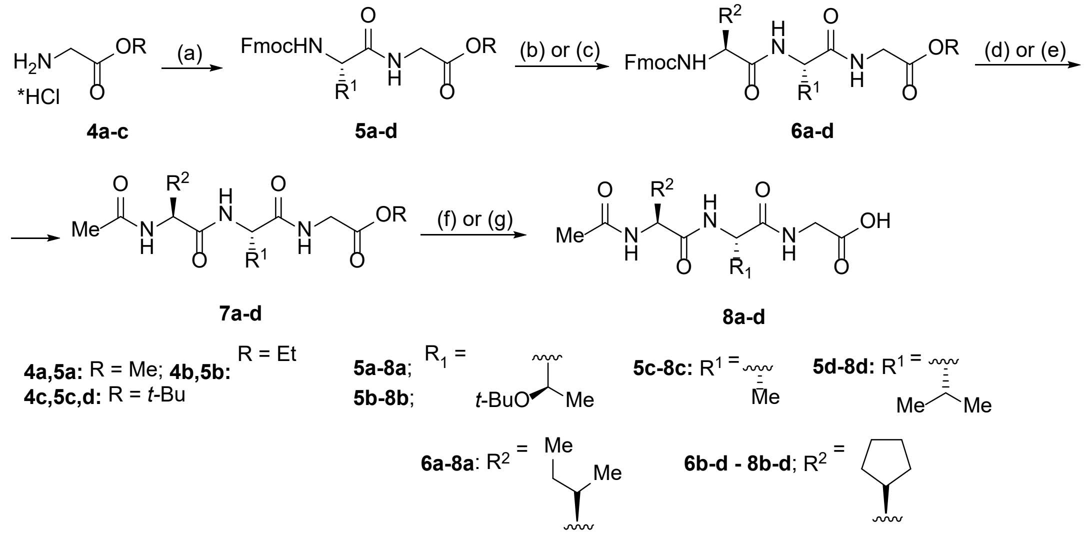

Scheme S1. Reagents and conditions: (a) N-Fmoc-amino acid, HATU, DIPEA, DCM, r. t. or EDC·HCl, HOBt, DIPEA, DCM, room temperature (r. t.); (b) for synthesis of **6a**: DMF, 120 °C, then Fmoc-Ile-OH, HATU, DIPEA, DMF, r. t; (c) for synthesis of **6b-d**: DMA, THF, r. t., then Fmoc-cyclopentyl-Gly-OH, EDC·HCl, HOBt, DIPEA, DCM, r. t.; (d) for synthesis of **7a**: DMF, 120 °C, then Ac_2O, DIPEA, DMF, r. t.; (e) for synthesis of **7b-d**: DMA, THF, r. t., then Ac_2O, DIPEA, DCM, r. t. (f) for synthesis of **8a-b**: LiOH, THF:H_2O (20:1), r. t. (g) for synthesis of **8c-d**: TFA, DCM, r.t.

Tripeptides **8a-d** were coupled with amino boronic esters **9a-g** and the resulting intermediates **10a-j** were deprotected to give peptidic boronic acids **3a-j** (Scheme S2).

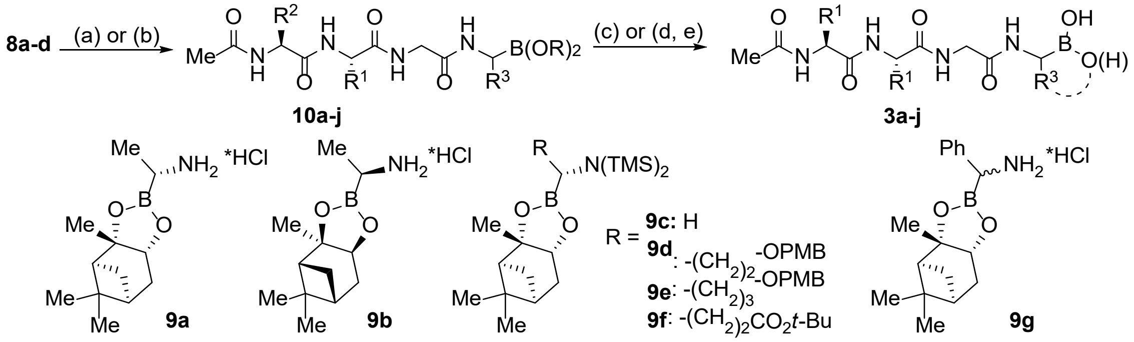

Scheme S2. Reagents and conditions: (a) for synthesis of **10a**: acid **8a**, α-amino boronic acid ester hydrochloride **9a**, HATU, DIPEA, DCM, r. t.; (b) for synthesis of **10b-j**: acids **8b-d**, α-amino boronic acid ester hydrochloride or TMS-protected α-amino boronic acid ester **9a-g**, T3P, NMM, EtOAc, r. t.; (c) for synthesis of **3a**: BBr_3, DCM, −78°C to r. t.; (d) for synthesis of **3b-j**: *i*BuB$(OH)_2$, MeCN/Hexane/1 M HCl, r. t.; (e) for synthesis of **3a-h**: TFA, DCM, r. t.

Boronic acid building blocks **9a-g** were prepared starting from commercially available boronic acid esters **11a,b** according to known procedures which involved Matteson homologation (Scheme S3-S8).

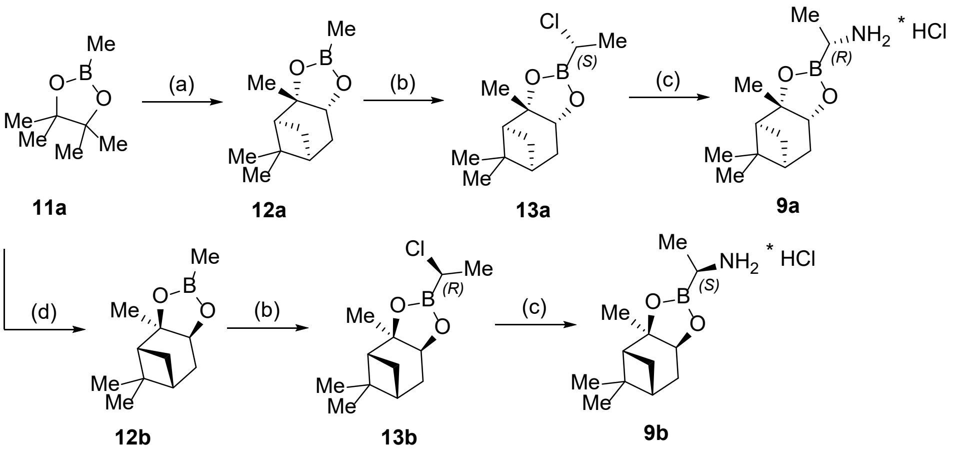

Scheme S3. Reagents and conditions: (a) (+)-pinanediol, THF, r. t.; (b) *n*-BuLi, DCM, −100°C, THF, then 1 M $ZnCl_2$, THF, −100°C to r.t. (c) LiHMDS, THF, −78°C to r.t., then HCl in diox., pentane, −78 °C to r. t. (d) (-)-pinanediol, THF, r. t.;

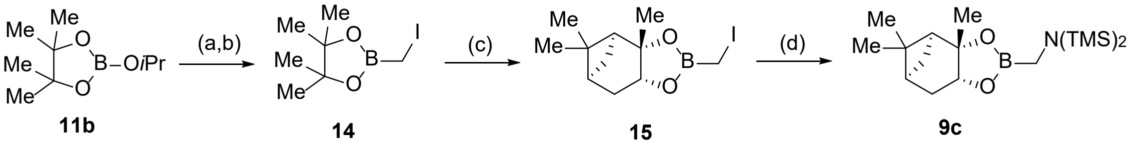

Scheme S4. Reagents and conditions: (a) ICH_2Cl, *n*-BuLi, then TMS-Cl THF, −78 °C to r. t.; (b) NaI, acetone, reflux; LiHMDS, THF, −78°C to r. t., (c) (+)-pinanediol, THF, r. t.; (d) LiHMDS, THF, −78°C to r.t.

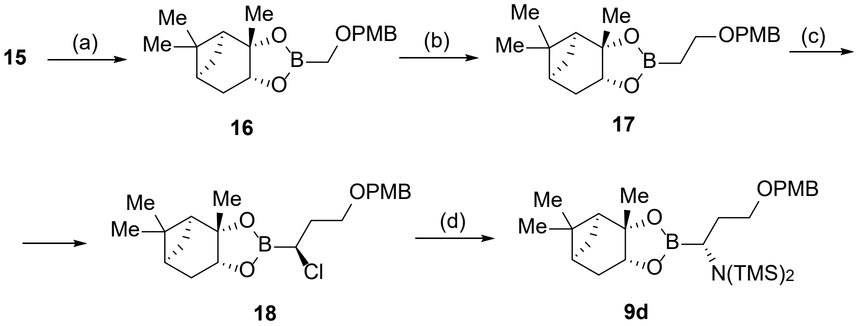

Scheme S5. Reagents and conditions: (a) PMB-OH, *n*-BuLi, DMSO, THF, −78 °C to r. t.; (b) ICH_2Cl, *n*-BuLi, Et_2O, −100°C to r. t.; (c) *n*-BuLi, DCM, −100 °C, THF, then 1M $ZnCl_2$, THF, −100°C; (d) LiHMDS, THF, −78°C to r.t.

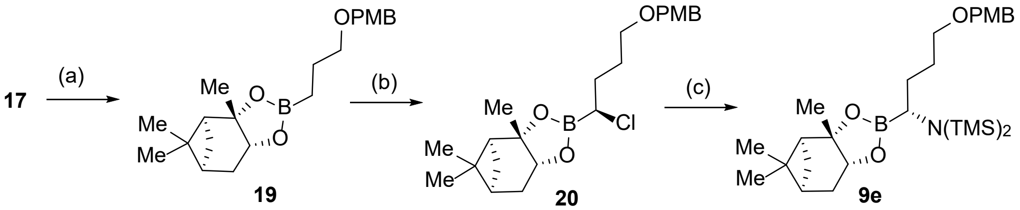

Scheme S6. Reagents and conditions: (a) ICH_2Cl, *n*-BuLi, Et_2O, −100°C (b) *n*-BuLi, DCM, −100°C, THF, then 1M $ZnCl_2$, THF, −100°C; (c) LiHMDS, THF, −78°C to r.t.

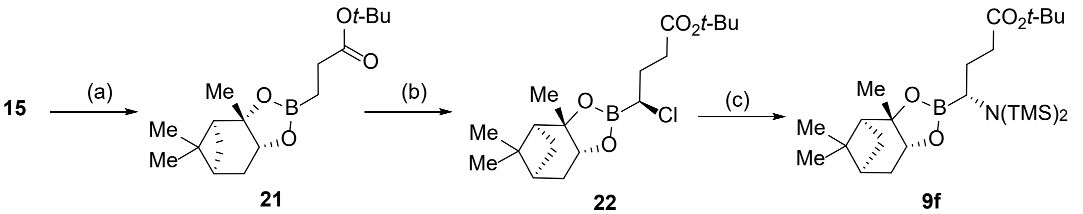

Scheme S7. Reagents and conditions: (a) *t*-Butyl acetate, LDA, THF, −78°C to r.t.; (b) *n*-BuLi, DCM, −100°C, THF, then 1 M $ZnCl_2$, THF, −100°C; (c) LiHMDS, THF, −78°C

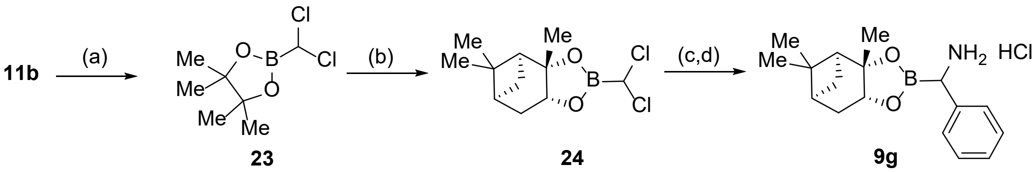

Scheme S8. Reagents and conditions: (a) *n*-BuLi, DCM, −100°C, THF, then 5 M HCl (b) (+)-pinanediol, THF, r. t.; (c); PhMgBr, $ZnCl_2$, THF, −78°C; (d) LiHMDS, THF, −78°C, then HCl in diox., hexane

2. Synthesis of peptidic boronic acids 3a-j: experimental procedures

General information.

Reagents and starting materials were obtained from commercial sources and used as received. The solvents were purified and dried by standard procedures prior to use. Flash chromatography was carried out using silica gel (230−400 mesh). Thin layer chromatography (TLC) was performed on silica gel and was visualized by UV light or staining with $KMnO_4$. NMR spectra were recorded on 300 and 400 MHz spectrometers with chemical shift values (δ) in parts per million using the residual chloroform or methanol signal as the internal standard. Conversion of starting material was detected with a UPLC Waters Acquity, column: Acquity UPLC BEH-C18, 1.7 µm, 2.1 mm x 50 mm, column temperature (30.0±5.0)°C, gradient: 0.01% TFA in water/CH_3CN 90%/10% – 5%/95%; flow: 0.500 mL/min; time: 8 min; detector: PDA, 220 – 320 nm, SQ detector with an electrospray ion source (ESI/APCI). Gas chromatographic (GC) analysis was performed on an Agilent Technologies gas chromatographer with a triple-axis detector, heating range 40 – 280°C, column 30 m x 0.25 mm, 0.25 µm, 7 inch cage. Exact molecular masses (HRMS) were determined on a hybrid quadrupole time-of-flight mass spectrometer equipped with an electrospray ion source. For reversed phase column chromatography a Biotage KP-C18-HS SNAP cartridge was used (gradient – water/CH_3CN).

Compound 5a. A mixture of glycine methyl ester hydrochloride (**4a**) (401 mg, 3.2 mmol, 1.0 equiv), Fmoc-Thr(*t*Bu)-OH (1.30 g, 3.2 mmol, 1.0 equiv), HATU (1.34 g, 3.5 mmol, 1.1 equiv) and DIPEA (1.66 mL, 9.6 mmol, 3.0 equiv) in DCM (20 mL) were stirred for 2 h at room temperature. The reaction mixture was washed with H_2O (2x20 mL) and then with brine (20 mL). The organic phase was dried over Na_2SO_4, filtered and evaporated in vacuo. The residue was purified by flash chromatography on silica gel eluting with hexane:EtOAc, 4:1 - 1:1 to provide **5a** (R=Me, 1.37 g, 92%) as a white solid.

^{1}H NMR (400 MHz, Chloroform-*d*) δ 7.76 (d, *J* = 7.4 Hz, 2H), 7.68 – 7.57 (m, 3H), 7.40 (t, *J* = 7.5 Hz, 2H), 7.31 (t, *J* = 7.3 Hz, 2H), 6.00 (d, *J* = 4.9 Hz, 1H), 4.45 – 4.34 (m, 2H), 4.28 – 4.16 (m, 3H), 4.16 – 4.00 (m, 2H), 3.78 (s, 3H), 1.31 (s, 9H), 1.09 (d, *J* = 6.4 Hz, 3H)

^{13}C NMR (101 MHz, Chloroform-*d*) δ 170.0, 169.8, 156.1, 144.0, 143.8, 141.45, 141.43, 127.8, 127.2, 125.3, 120.12, 120.10, 75.8, 67.1, 66.7, 58.7, 52.5, 47.3, 41.5, 28.3, 16.9

HR-MS (ESI/TOF) calculated for $C_{26}H_{33}N_2O_6$ $[M+H]^+$ 469.2339, found 469.2337

Compound 5b was synthesised analogous to the synthesis of **5a** by using glycine ethyl ester hydrochloride (**4b**) (2.00 g, 14.3 mmol), Fmoc-Thr(*t*Bu)-OH (5.81 g, 14.3 mmol, 1.0 equiv), EDC*HCl (4.12 g, 21.5 mmol, 1.5 equiv), HOBt (2.52 g, 18.6 mmol, 1.3 equiv) and DIPEA (7.5 mL, 43.0 mmol, 3.0 equiv) in DCM (50 mL). Yield: 6.25 g, 90 % (white solid).
^{1}H NMR (400 MHz, Chloroform-*d*) δ 7.81 – 7.70 (m, 2H), 7.67 – 7.53 (m, 3H), 7.46 – 7.35 (m, 2H), 7.36 – 7.27 (m, 2H), 6.01 (d, *J* = 5.2 Hz, 1H), 4.46 – 4.34 (m, 2H), 4.29 – 4.14 (m, 5H), 4.07 (qd, *J* = 18.3, 5.5 Hz, 2H), 1.35 – 1.24 (m, 12H), 1.10 (d, *J* = 6.4 Hz, 3H)
^{13}C NMR (100 MHz, Chloroform-*d*) δ 169.7, 169.5, 156.1, 144.0, 143.8, 141.44, 141.42, 127.8, 127.2, 125.3, 120.11, 120.09, 75.8, 67.1, 66.7, 61.6, 58.7, 47.3, 41.7, 28.3, 16.9, 14.3
HR-MS (ESI/TOF) calculated for $C_{27}H_{35}N_2O_6$ $[M+H]^+$ 483.2495, found 483.2494.

Compound 5c was synthesised analogous to the synthesis of **5a** by using glycine *t*-butyl ester hydrochloride (**4c**) (300 mg, 1.8 mmol), N-Fmoc-Ala-OH (560 mg, 1.8 mmol, 1 equiv), HOBt (266 mg, 2.0 mmol, 1.1 equiv), EDC*HCl (412 mg, 2.15 mmol, 1.2 equiv) and DIPEA (930 µL, 5.4 mmol, 3.0 equiv). Yield: 624 mg, 82% (white solid).
^{1}H NMR (400 MHz, Methanol-*d*$_4$) δ 7.81 – 7.75 (m, 2H), 7.66 (t, *J* = 7.2 Hz, 2H), 7.38 (t, *J* = 7.5 Hz, 2H), 7.30 (t, *J* = 7.5 Hz, 2H), 4.36 (d, *J* = 6.4 Hz, 2H), 4.25 – 4.13 (m, 2H), 3.91 – 3.73 (m, 2H), 1.45 (s, 9H), 1.36 (d, *J* = 7.3 Hz, 3H).
^{13}C NMR (101 MHz, Methanol-*d*$_4$) δ 174.6, 168.9, 156.8, 144.0, 143.8, 141.2, 127.4, 126.8, 126.8, 124.8, 124.8, 119.5, 81.4, 66.6, 50.5, 41.4, 26.9, 16.9.
HR-MS (ESI/TOF) calculated for $C_{24}H_{28}N_2O_5Na$ $[M+Na]^+$ 447.1896, found 447.1909

Compound 5d was synthesised analogous to the synthesis of **5a** by using glycine *t*-butyl ester hydrochloride (**4c**) (300 mg, 1.8 mmol), N-Fmoc-Val-OH (607 mg, 1.8 mmol, 1 equiv), HOBt (266 mg, 2.0 mmol, 1.1 equiv), EDC*HCl (412 mg, 2.15 mmol, 1.2 equiv) and DIPEA (930 µL, 5.4 mmol, 3.0 equiv). Yield: 725 mg, 90% (white solid).
^{1}H NMR (400 MHz, Methanol-*d*$_4$) δ 7.79 (d, *J* = 7.5 Hz, 2H), 7.66 (t, *J* = 6.4 Hz, 2H), 7.38 (t, *J* = 7.5 Hz, 2H), 7.30 (t, *J* = 7.5 Hz, 2H), 4.41 (dd, *J* = 10.6, 7.1 Hz, 1H), 4.34 (dd, *J* = 10.6, 6.6 Hz, 1H), 4.22 (t, *J* = 6.9 Hz, 1H), 3.96 (d, *J* = 7.0 Hz, 1H), 3.92 – 3.72 (m, 2H), 2.09 (h, *J* = 6.8 Hz, 1H), 1.44 (s, 9H), 0.97 (dd, *J* = 11.0, 6.8 Hz, 6H).
^{13}C NMR (101 MHz, Methanol-*d*$_4$) δ 174.6, 170.1, 158.6, 145.3, 145.2, 142.6, 128.8, 128.2, 128.1, 126.2, 120.9, 82.8, 67.9, 62.0, 42.8, 31.9, 28.3, 19.8, 18.5.
HR-MS (ESI/TOF) calculated for $C_{26}H_{32}N_2O_5Na$ $[M+Na]^+$ 475.2209, found 475.2213

Compound 6a. Thermal Fmoc- group cleavage was performed[1] as the first step. A mixture of **5a** (279 mg, 0.60 mmol) in DMF (4 mL) was refluxed at 120°C for 2 h (until full conversion) then the reaction mixture was cooled to room temperature. Under an argon atmosphere *N*-Fmoc-L-isoleucine (211 mg, 0.60 mmol, 1.0 equiv), HATU (272 mg, 0.72 mmol, 1.2 equiv) and DIPEA (310 µL, 1.80 mmol, 3.0 equiv) were added to the solution of amine (147 mg, 0.60 mmol based on a theoretical yield of 100%). The reaction mixture was stirred for 4 h at room temperature, then diluted with EtOAc (20 mL), washed with H_2O (3x10 mL) and brine (10 mL). The organic phase was dried over Na_2SO_4, filtered and evaporated in vacuo. The residue was purified by flash chromatography on silica gel eluting with hexane:EtOAc (2:1) – pure EtOAc to provide **6a** (253 mg, 73%) as a white solid.
^{1}H NMR (400 MHz, Chloroform-*d*) δ 7.76 (d, *J* = 7.2 Hz, 2H), 7.68 (t, *J* = 5.2 Hz, 1H), 7.61 (d, *J* = 7.5 Hz, 2H), 7.40 (t, *J* = 7.5 Hz, 2H), 7.31 (t, *J* = 7.4 Hz, 2H), 6.93 (d, *J* = 5.7 Hz, 1H), 5.44 (d, *J* = 8.3 Hz, 1H), 4.45 (dd, *J* = 10.6, 7.4 Hz, 1H), 4.41 – 4.31 (m, 2H), 4.25 – 4.18 (m, 2H), 4.14 (dd, *J* = 8.3, 5.6 Hz, 1H), 4.11 – 3.99 (m, 2H), 3.75 (s, 3H), 1.94 – 1.79 (m, 1H), 1.60 – 1.45 (m, 1H), 1.30 (s, 9H), 1.28 – 1.12 (m, 1H), 1.04 (d, *J* = 6.4 Hz, 3H), 0.98 – 0.87 (m, 6H)
^{13}C NMR (101 MHz, Chloroform-*d*) δ 171.1, 170.0, 169. 7, 156.3, 144.1, 143.9, 141.4, 127.8, 127.2, 125.3, 125.2, 120.12, 120.10, 75.8, 67.2, 66.1, 59.7, 57.6, 52.4, 47.3, 41.5, 38.1, 28.3, 25.1, 17.2, 15.6, 11.7
HR-MS (ESI/TOF) calculated for $C_{32}H_{43}N_3O_7Na$ $[M+Na]^+$ 604.2999, found 604.3001

Compound 6b. Based on the literature[2] an Fmoc- group cleavage was performed. Starting material **5b** (2.21 g, 4.6 mmol, 1.0 equiv) in THF (30 mL) was treated with 2 M dimethylamine in THF (9.2 mL) while stirring under an argon atmosphere. After 10 min the solvent was evaporated, and the residue was treated with 2 M dimethylamine in THF as before for an additional 40 min. After solvent evaporation the residue was taken to the next step without purification. Crude mixture (1.19 g, 4.6 mmol based on a theoretical yield of 100%), Fmoc-cyclopentyl-Gly-OH (1.67 g, 4.6 mmol, 1.0 equiv), EDC*HCl (6.3 g, 6.8 mmol, 1.5 equiv), HOBt (0.68 g, 5.0 mmol, 1.1 equiv) and DIPEA (2.4 mL, 13.9 mmol, 3.0 equiv) in DCM (40 mL) was stirred for 2 h at room temperature, then washed with 2 M HCl (3x20 mL) and brine (20 mL). The organic phase was dried over Na_2SO_4, filtered and evaporated in vacuo. The residue was purified by flash chromatography on silica gel eluting with Hexane:$CHCl_3$:EtOAc (8:1:1) – 100 % of $CHCl_3$:EtOAc (1:1) to provide **6b** (2.44 g, 88%) as a white solid.
^{1}H NMR (300 MHz, Chloroform-*d*) δ 7.76 (d, *J* = 7.2 Hz, 2H), 7.63 (dd, *J* = 16.0, 6.3 Hz, 3H), 7.40 (t, *J* = 7.5 Hz, 2H), 7.31 (t, *J* = 7.4 Hz, 2H), 6.93 (d, *J* = 5.7 Hz, 1H), 5.38 (d, *J* = 8.4 Hz, 1H), 4.45 (dd, *J* = 10.5, 7.2 Hz, 1H), 4.35 (t, *J* = 8.5 Hz, 2H), 4.28 – 4.15 (m, 4H), 4.17 – 3.95 (m, 3H), 2.33 – 2.13 (m, 1H), 1.84 – 1.44 (m, 6H), 1.41 – 1.19 (m, 14H), 1.04 (d, *J* = 6.5 Hz, 3H).
^{13}C NMR (101 MHz, Chloroform-*d*) δ 171.6, 169.6, 169.5, 156.4, 144.1, 143.9, 141.4, 127.8, 127.2, 125.3, 125.2, 120.11, 120.08, 75.7, 67.2, 66.1, 61.6, 58.7, 57.6, 47.3, 43.2, 41.7, 29.6, 28.6, 28.3, 25.4, 25.1, 17.2, 14.3
HR-MS (ESI/TOF) calculated for $C_{34}H_{46}N_3O_7$ $[M+H]^+$ 608.3336, found 608.3321

Compound 6c was synthesised by the same method as **6b**. Starting material **5c** (300 mg, 0.71 mmol, 1.0 equiv) in THF (15 mL) was treated with 2M dimethylamine in THF (1.4 mL) while stirring under an inert atmosphere. Then the residue (143 mg, 0.71 mmol based on a theoretical yield of a 100%) was dissolved in DCM (30 mL), Fmoc-cyclopentyl-Gly-OH (258 mg, 0.71 mmol, 1.0 equiv), EDC*HCl (163 mg, 0.85 mmol, 1.2 equiv), HOBt (105 mg, 0.78 mmol, 1.1 equiv) and DIPEA (370 µL, 2.14 mmol, 3.0 equiv) were added. The residue was purified by flash chromatography on silica gel eluting with 1% MeOH/$CHCl_3$ - 5 % MeOH/$CHCl_3$. Yield: 225 mg, 58 % (white solid).
^{1}H NMR (400 MHz, Chloroform-*d*) δ 7.75 (d, *J* = 7.6 Hz, 2H), 7.58 (d, *J* = 7.5 Hz, 2H), 7.38 (t, *J* = 7.5 Hz, 2H), 7.29 (t, *J* = 7.5 Hz, 2H), 6.82 (t, *J* = 4.9 Hz, 1H), 6.74 (d, *J* = 7.9 Hz, 1H), 5.66 (d, *J* = 8.6 Hz, 1H), 4.62 (p, *J* = 7.3 Hz, 1H), 4.43 (dd, *J* = 10.6, 7.2 Hz, 1H), 4.34 (dd, *J* = 10.7, 7.1 Hz, 1H), 4.20 (t, *J* = 7.2 Hz, 1H), 4.10 (t, *J* = 8.3 Hz, 1H), 4.00 – 3.84 (m, 2H), 2.23 (p, *J* = 8.2 Hz, 1H), 1.79 – 1.67 (m, 2H), 1.66 – 1.48 (m, 4H), 1.44 (s, 9H), 1.39 (d, *J* = 7.2 Hz, 3H), 1.36 – 1.24 (m, 2H).
^{13}C NMR (101 MHz, Chloroform-*d*) δ 172.1, 171.8, 168.8, 156.6, 144.0, 143.9, 141.4, 127.9, 127.2, 125.3, 125.2, 120.11, 120.09, 82.5, 67.2, 58.9, 48.8, 47.3, 42.9, 42.2, 29.6, 28.8, 28.1, 25.5, 25.1, 18.5.
HR-MS (ESI/TOF) calculated for $C_{31}H_{39}N_3O_6Na$ $[M+Na]^+$ 572.2737, found 572.2740

Compound 6d was synthesised by the same method as **6b**. Starting material **5d** (350 mg, 0.77 mmol, 1.0 equiv) in THF (15 mL) was treated with 2 M dimethylamine in THF (1.6 mL) while stirring under an inert atmosphere. Then the residue (178 mg, 0.77 mmol based on a theoretical yield of a 100%) was dissolved in DCM (35 mL), Fmoc-cyclopentyl-Gly-OH (282 mg, 0.77 mmol, 1.0 equiv), EDC*HCl (178 mg, 0.93 mmol, 1.2 equiv), HOBt (115 mg, 0.85 mmol, 1.1 equiv) and DIPEA (400 µL, 2.31 mmol, 3.0 equiv) were added. The residue was purified by flash chromatography on silica gel eluting with 1% MeOH/$CHCl_3$ - 5% MeOH/$CHCl_3$. Yield: 238 mg, 53% (white solid).
^{1}H NMR (400 MHz, Chloroform-*d*) δ 7.74 (d, *J* = 7.5 Hz, 2H), 7.57 (dd, *J* = 7.5, 3.2 Hz, 2H), 7.38 (t, *J* = 7.5 Hz, 2H), 7.31 – 7.26 (m, 2H, overlaps with solvent), 6.85 – 6.69 (m, 2H), 5.73 (d, *J* = 8.5 Hz, 1H), 4.49 – 4.38 (m, 2H), 4.33 (dd, *J* = 10.7, 7.0 Hz, 1H), 4.19 (t, *J* = 7.2 Hz, 1H), 4.12 (t, *J* = 8.5 Hz, 1H), 4.01 (dd, *J* = 18.2, 5.6 Hz, 1H), 3.84 (dd, *J* = 18.2, 4.8 Hz, 1H), 2.26 (p, *J* = 8.4 Hz, 1H), 2.19 – 2.07 (m, 1H), 1.81 – 1.67 (m, 2H), 1.66 – 1.48 (m, 4H), 1.44 (s, 9H), 1.37 – 1.23 (m, 2H), 1.01 – 0.85 (m, 6H).
^{13}C NMR (101 MHz, Chloroform-*d*) δ 172.1, 171.1, 168.8, 156.6, 144.0, 143.9, 141.4, 127.8, 127.2, 125.3, 125.2, 120.08, 120.06, 82.4, 67.2, 59.2, 58.4, 47.3, 42.7, 42.2, 31.1, 29.6, 29.0, 28.2, 25.5, 25.2, 19.3, 18.2.

HR-MS (ESI/TOF) calculated for $C_{33}H_{43}N_3O_6Na$ [M+Na]$^+$ 600.3050, found 600.3061

Compound 7a. A mixture of **6a** (246 mg, 0.42 mmol, 1.0 equiv) in DMF (4 mL) was refluxed at 120 ^oC for 2 h (till full conversion) then reaction mixture was cooled to r. t. Under an argon atmosphere acetic anhydride (60 µL, 0.63 mmol, 1.5 equiv) and DIPEA (146 µL, 0.84 mmol, 2.0 equiv) were added to the solution of amine (152 mg, 0.42 mmol, based on a theoretical yield of 100 %). The reaction mixture was stirred for 5 h at room temperature, then diluted with EtOAc (20 mL) washed with H_2O (3x10 mL) and brine (10 mL). The organic phase was dried over Na_2SO_4, filtered and evaporated in vacuo. The residue was purified by flash chromatography on silica gel eluting with Hexane:EtOAc (1:1) – pure EtOAc to provide **7a** (114 mg, 67%) as a white solid.
^{1}H NMR (400 MHz, Chloroform-*d*) δ 7.66 (t, *J* = 5.2 Hz, 1H), 6.91 (d, *J* = 5.8 Hz, 1H), 6.25 (d, *J* = 8.3 Hz, 1H), 4.43 – 4.33 (m, 2H), 4.16 (qd, *J* = 6.4, 3.8 Hz, 1H), 4.15 – 3.97 (m, 2H), 3.75 (s, 3H), 2.02 (s, 3H), 1.87 – 1.76 (m, 1H), 1.57 – 1.42 (m, 1H), 1.28 (s, 9H), 1.25 – 1.08 (m, 1H), 1.04 (d, *J* = 6.4 Hz, 3H), 0.96 – 0.85 (m, 6H)
^{13}C NMR (101 MHz, Chloroform-*d*) δ 171.2, 170.1, 170.0, 169.6, 75.7, 66.2, 57.8, 57.6, 52.4, 41.5, 38.1, 28.3, 25.3, 23.4, 17.2, 15.5, 11.6
UPLC-MS (ESI) calculated for $C_{19}H_{36}N_3O_6$ [M+H]$^+$ 402.51, found 402.55

Compound 7b. Starting material **6b** (2.31 g, 3.8 mmol, 1.0 equiv) in THF (30 mL) was treated with 2 M dimethylamine in THF (7.6 mL) while stirring under an inert atmosphere. After 10 min, the solvent was evaporated, and the residue was treated with 2 M dimethylamine in THF as before for an additional 40 min. After solvent evaporation the residue was utilized in the next step without purification. The residue (1.465 g, 3.8 mmol based on a theoretical yield of 100%) was dissolved in DCM (40 mL), acetic anhydride (540 µL, 5.7 mmol, 1.5 equiv) and DIPEA (1.3 mL, 7.5 mmol, 2.0 equiv) were added. Reaction mixture was stirred for 2 h at room temperature, then washed with 2 M HCl (3x20 mL) and brine (20 mL). The organic phase was dried over Na_2SO_4, filtered and evaporated in vacuo. The crude mixture was purified by flash chromatography on silica gel eluting with $CHCl_3$:EtOAc (1:1) – 2 % MeOH in $CHCl_3$:EtOAc (1:1) to provide **7b** (1.16 g, 72%) as a white solid.
^{1}H NMR (400 MHz, Chloroform-*d*) δ 7.63 (t, *J* = 5.2 Hz, 1H), 6.95 (d, *J* = 6.0 Hz, 1H), 6.26 (d, *J* = 8.2 Hz, 1H), 4.43 – 4.32 (m, 2H), 4.21 (q, *J* = 7.2 Hz, 2H), 4.20 – 4.11 (m, 1H), 4.14 – 3.95 (m, 2H), 2.18 (h, *J* = 8.3, 7.9 Hz, 1H), 2.01 (s, 3H), 1.76 – 1.66 (m, 2H), 1.65 – 1.57 (m, 2H), 1.57 – 1.46 (m, 2H), 1.36 – 1.24 (d, *J* = 3.7 Hz, 14H), 1.04 (d, *J* = 6.4 Hz, 3H)
^{13}C NMR (101 MHz, Chloroform-*d*) δ 171.7, 170.1, 169.6, 169.5, 75.7, 66.2, 61.6, 57.6, 56.9, 43.1, 41.7, 29.6, 28.9, 28.3, 25.4, 25.0, 23.4, 17.2, 14.3
HR-MS (ESI/TOF) calculated for $C_{21}H_{37}N_3O_6Na$ [M+Na]$^+$ 450.2580, found 450.2586

Compound 7c was synthesised by the same method as **7b**. Starting material **6c** (200 mg, 0.36 mmol, 1.0 equiv) in THF (10 mL) was treated with 2 M dimethylamine in THF (730 µL) while stirring under an inert atmosphere. Then the residue (119 mg, 0.36 mmol based on a theoretical yield of a 100 %) was dissolved in DCM (20 mL), acetic anhydride (50 µL, 0.53 mmol, 1.5 equiv) and DIPEA (130 µL, 0.75 mmol, 2.0 equiv) were added. The residue was purified by flash chromatography on silica gel eluting with 1% MeOH/$CHCl_3$ - 5% MeOH/$CHCl_3$. Yield: 93 mg, 69% (white solid).
^{1}H NMR (400 MHz, Chloroform-*d*) δ 7.22 – 7.09 (m, 2H), 6.70 (d, *J* = 8.7 Hz, 1H), 4.70 (p, *J* = 7.0 Hz, 1H), 4.47 (t, *J* = 8.6 Hz, 1H), 4.04 – 3.82 (m, 2H), 2.19 (h, *J* = 8.9 Hz, 1H), 2.03 (s, 3H), 1.74 – 1.64 (m, 2H), 1.64 – 1.55 (m, 2H), 1.54 – 1.48 (m, 2H), 1.46 (s, 9H), 1.38 (d, *J* = 7.0 Hz, 3H), 1.36 – 1.24 (m, 2H).
^{13}C NMR (101 MHz, Chloroform-*d*) δ 172.4, 171.9, 170.4, 168.9, 82.4, 56.9, 48.7, 43.3, 42.3, 29.5, 29.0, 28.2, 25.4, 25.0, 23.3, 18.7.
HR-MS (ESI/TOF) calculated for $C_{18}H_{31}N_3O_5Na$ [M+Na]$^+$ 392.2161, found 392.2172

Compound 7d was synthesised by the same method as **7b**. Starting material **6d** (200 mg, 0.35 mmol, 1.0 equiv) in THF (10 mL) was treated with 2M dimethylamine in THF (700 µL) while stirring under inert atmosphere. Then the residue (123 mg, 0.35 mmol based on a theoretical yield of 100%) was dissolved in DCM (20 mL), acetic anhydride (50 µL, 0.53 mmol, 1.5 equiv) and

DIPEA (120 µL, 0.70 mmol, 2.0 equiv) were added. The residue was purified by flash chromatography on silica gel eluting with 1% MeOH/$CHCl_3$ - 5% MeOH/$CHCl_3$. Yield: 63 mg, 46% (white solid).
^{1}H NMR (400 MHz, Chloroform-*d*) δ 7.06 – 6.97 (m, 2H), 6.64 (d, *J* = 8.6 Hz, 1H), 4.46 – 4.38 (m, 2H), 4.05 (dd, *J* = 18.2, 5.8 Hz, 1H), 3.82 (dd, *J* = 18.2, 4.5 Hz, 1H), 2.25 – 2.14 (m, 1H), 2.19 – 2.05 (m, 1H), 2.01 (s, 3H), 1.77 – 1.63 (m, 2H), 1.64 – 1.56 (m, 2H), 1.56 – 1.48 (m, 2H), 1.46 (s, 9H), 1.37 – 1.26 (m, 2H), 0.95 (d, *J* = 6.7 Hz, 3H), 0.93 (d, *J* = 6.8 Hz, 3H).
^{13}C NMR (101 MHz, Chloroform-*d*) δ 172.2, 171.3, 170.4, 169.0, 82.4, 58.5, 57.3, 42.8, 42.2, 31.0, 29.6, 29.2, 28.2, 25.5, 25.1, 23.2, 19.3, 18.3.
HR-MS (ESI/TOF) calculated for $C_{20}H_{35}N_3O_5Na$ $[M+Na]^+$ 420.2474, found 420.2485

Compound 8a. Starting material **7a** (114 mg, 0.28 mmol, 1.0 equiv) was dissolved in THF:H_2O (20:1, 5 mL), then LiOH (68 mg, 2.84 mmol, 10 equiv) was added and the reaction was stirred for 20 h at r. t.. Water (5 mL) was added and the reaction mixture was acidified to pH ~2 by the addition of 1 M HCl and the product was extracted with EtOAc (4x5mL). The organic phase was washed with brine, dried over Na_2SO_4, filtered and evaporated in vacuo to provide product **8a** (107 mg, 97 %) as a white solid.
^{1}H NMR (400 MHz, Chloroform-*d*) δ 7.66 (t, *J* = 4.9 Hz, 1H), 7.54 (d, *J* = 6.5 Hz, 1H), 6.75 (d, *J* = 8.8 Hz, 1H), 4.56 (dd, *J* = 8.8, 7.0 Hz, 1H), 4.46 (dd, *J* = 6.6, 3.7 Hz, 1H), 4.19 – 4.04 (m, 3H), 2.05 (s, 3H), 1.84 – 1.72 (m, 1H), 1.57 – 1.46 (m, 1H), 1.26 (s, 9H), 1.21 – 1.08 (m, 1H), 1.01 (d, *J* = 6.4 Hz, 3H), 0.96 – 0.83 (m, 6H).
^{13}C NMR (101 MHz, Chloroform-*d*) δ 171.7, 171.6, 171.0, 169.8, 75.6, 66.4, 57.8, 42.0, 38.1, 28.3, 25.1, 23.3, 17.5, 15.5, 11.4.
HR-MS (ESI/TOF) calculated for $C_{18}H_{33}N_3O_6Na$ $[M+Na]^+$ 410.2267, found 410.2275.

Compound 8b was synthesised by the same method as **8a**. Starting material **7b** (1.70 g, 4.0 mmol, 1.0 equiv), LiOH (955 mg, 39.9 mmol, 10 equiv) in THF:H_2O (1:1, 42 mL) were stirred for 20 h at room temperature. Product **8b** was obtained (1.56 g, 98%) as a white solid.
^{1}H NMR (400 MHz, Chloroform-*d*) δ 10.85 (br s, 1H), 7.69 (t, *J* = 5.0 Hz, 1H), 7.63 (d, *J* = 6.5 Hz, 1H), 6.92 (d, *J* = 8.8 Hz, 1H), 4.59 (t, *J* = 8.5 Hz, 1H), 4.46 (dd, *J* = 6.5, 3.9 Hz, 1H), 4.21 – 4.04 (m, 2H), 2.15 (h, *J* = 8.3 Hz, 1H), 2.03 (s, 3H), 1.75 – 1.55 (m, 4H), 1.56 – 1.42 (m, 2H), 1.41 – 1.29 (m, 2H), 1.27 (s, 9H), 1.00 (d, *J* = 6.4 Hz, 3H).
^{13}C NMR (101 MHz, Chloroform-*d*) δ 172.2, 171.5, 171.1, 169.7, 75.7, 66.5, 57.7, 56.9, 43.4, 42.0, 29.4, 29.0, 28.2, 25.3, 24.9, 23.2, 17.3.
HR-MS (ESI/TOF) calculated for $C_{19}H_{33}N_3O_6Na$ $[M+Na]^+$ 422.2267, found 422.2268.

Compound 8c. Starting material **7c** (93 mg, 0.25 mmol) was dissolved in DCM (2 mL), then TFA (500 µL) was added and the reaction was stirred for 20 h at room temperature. Solvent was evaporated in vacuo and product **8c** was transferred to the next reaction without purification.

Compound 8d. Starting material **7d** (63 mg, 0.16 mmol) was dissolved in DCM (2 mL), then TFA (500 µL) was added and the reaction was stirred for 20 h at room temperature. Solvent was evaporated in vacuo and product **8d** was transferred to the next reaction without purification.
Compound 10a. A mixture of intermediate **9a** (40 mg, 0.15 mmol, 1.0 equiv), **8a** (60 mg, 0.15 mmol, 1.0 equiv), HATU (71 mg, 0.19 mmol, 1.2 equiv) and DIPEA (80 µL, 0.46 mmol, 3.0 equiv) in DCM (4 mL) was stirred for 2 h at room temperature. The reaction mixture was washed with H_2O (2x10 mL) and brine (10 mL). The organic phase was dried over Na_2SO_4, filtered and evaporated in vacuo. The residue was purified by flash chromatography on silica gel eluting with 0−5% MeOH in EtOAc to provide **10a** (50 mg, 55 %) as a solid compound.
^{1}H NMR (400 MHz, Chloroform-*d*) δ 7.78 (d, *J* = 6.4 Hz, 1H), 7.62 (t, *J* = 5.5 Hz, 1H), 7.54 (d, *J* = 4.1 Hz, 1H), 6.39 (d, *J* = 8.5 Hz, 1H), 4.68 (dd, *J* = 8.5, 6.1 Hz, 1H), 4.40 (dd, *J* = 6.3, 3.9 Hz, 1H), 4.26 (dd, *J* = 8.8, 2.1 Hz, 1H), 4.23 – 4.00 (m, 3H), 3.07 – 2.95 (m, 1H), 2.36 – 2.26 (m, 1H), 2.20 – 2.09 (m, 1H), 2.06 – 1.96 (m, 4H), 1.90 – 1.70 (m, 3H), 1.56 – 1.45 (m, 1H), 1.37 (s, 3H), 1.35 – 1.23 (m, 13H), 1.20 – 1.15 (m, 3H), 1.14 – 1.05 (m, 1H), 0.95 (d, *J* = 6.4 Hz, 3H), 0.91 – 0.80 (m, 9H).

^{13}C NMR (101 MHz, Chloroform-*d*) δ 171.4, 170.2, 170.0, 169.4, 85.2, 77.5, 75.6, 66.5, 58.0, 57.5, 51.8, 41.6, 39.8, 38.7, 38.3, 36.0, 33.8 (CHB, broad signal), 28.8, 28.3, 27.3, 26.5, 25.0, 24.2, 23.6, 17.6, 16.7, 15.5, 11.7.
HR-MS (ESI/TOF) calculated for $C_{30}H_{54}BN_4O_7$ $[M+H]^+$ 593.4086, found 593.4089.

Compound 10b. An acid **8b** (100 mg, 0.25 mmol, 1.0 equiv) was dissolved in 5 mL EtOAc, then *N*-methylmorpholine (85 µL, 0.75 mmol, 3.0 equiv) and a solution of propylphosphonic acid anhydride (300 µL, 2.0 equiv, 50 % by weight in EtOAc) was added sequentially. Reaction mixture was stirred for 30 min before **9a** (78 mg, 0.30 mmol, 1.2 equiv) was added. After the reaction was complete (UPLC-MS control) it was diluted with 5 mL of H_2O and AcOH (pH 3-4) were added. The layers were separated and the aqueous layer was extracted with EtOAc (2x5 mL). The combined organic layers were washed with saturated $NaHCO_3$ (10 mL), brine (10 mL), dried over $MgSO_4$, filtered and concentrated under reduced pressure. The crude mixture was purified by flash chromatography on silica gel eluting with 0-5% MeOH in EtOAc to provide **10b** (97 mg, 64%) as a solid compound.
^{1}H NMR (400 MHz, Chloroform-*d*) δ 7.86 (d, *J* = 6.4 Hz, 1H), 7.63 – 7.52 (m, 2H), 6.39 (d, *J* = 8.5 Hz, 1H), 4.71 (t, *J* = 8.0 Hz, 1H), 4.41 (dd, *J* = 6.5, 4.0 Hz, 1H), 4.26 (dd, *J* = 8.8, 2.1 Hz, 1H), 4.25 – 4.06 (m, 2H), 4.05 – 3.98 (m, 1H), 3.07 – 2.97 (m, 1H), 2.36 – 2.25 (m, 1H), 2.23 – 2.08 (m, 2H), 2.06 – 1.96 (m, 4H), 1.91 – 1.78 (m, 2H), 1.71 – 1.53 (m, 4H), 1.53 – 1.43 (m, 2H), 1.38 (s, 3H), 1.36 – 1.24 (m, 15H), 1.19 (d, *J* = 7.4 Hz, 3H), 0.95 (d, *J* = 6.4 Hz, 3H), 0.84 (s, 3H).
^{13}C NMR (101 MHz, Chloroform-*d*) δ 171.8, 170.1, 170.0, 169.4, 85.2, 77.5, 75.6, 66.6, 58.0, 56.3, 51.8, 44.1, 41.7, 39.8, 38.3, 36.0, 33.8 (CHB, broad signal), 29.2, 28.9, 28.8, 28.3, 27.3, 26.5, 25.2, 24.9, 24.2, 23.6, 17.5, 16.7.
HR-MS (ESI/TOF) calculated for $C_{31}H_{54}BN_4O_7$ [M+H]+ 605.4086, found 605.4103

The same procedure was used for **10c-j**
10c: synthesized from **8b** and **9b**. Yield: 82 mg (54%) as a solid compound.
1H NMR (400 MHz, Chloroform-*d*) δ 7.93 (d, *J* = 6.3 Hz, 1H), 7.67 (t, *J* = 5.2 Hz, 1H), 7.49 (d, *J* = 3.9 Hz, 1H), 6.36 (d, *J* = 8.5 Hz, 1H), 4.73 (t, *J* = 8.1 Hz, 1H), 4.39 (dd, *J* = 6.3, 3.9 Hz, 1H), 4.25 (dd, *J* = 8.8, 2.2 Hz, 1H), 4.24 – 3.99 (m, 3H), 3.14 – 3.04 (m, 1H), 2.38 – 2.26 (m, 1H), 2.20 – 2.09 (m, 2H), 2.06 – 1.97 (m, 4H), 1.92 – 1.80 (m, 2H), 1.74 – 1.53 (m, 4H), 1.53 – 1.43 (m, 2H), 1.39 (s, 3H), 1.36 – 1.23 (m, 15H), 1.20 (d, *J* = 7.4 Hz, 3H), 0.93 (d, *J* = 6.5 Hz, 3H), 0.84 (s, 3H).
13C NMR (101 MHz, Chloroform-*d*) δ 171.8, 170.1, 169.7, 169.4, 85.3, 77.6, 75.6, 66.5, 58.0, 56.3, 51.8, 44.2, 42.1, 39.8, 38.3, 35.9, 33.4 (CHB, broad signal), 29.2, 28.9, 28.7, 28.3, 27.3, 26.5, 25.2, 24.9, 24.2, 23.6, 17.6, 16.6.
HR-MS (ESI/TOF) calculated for $C_{31}H_{54}BN_4O_7$ [M+H]+ 605.4086, found 605.4084

10d: synthesized from **8b** and **9c.** A slight synthetic modification[3] was made for TMS protected building block **9c**: it was dissolved in 2 mL EtOAc, 1.0 equivalent of dry methanol was added, then it was stirred for 30 min and added to the solution of activated acid. Yield: 50 mg (31%) as a solid compound.
^{1}H NMR (400 MHz, Chloroform-*d*) δ 7.83 (d, *J* = 6.1 Hz, 1H), 7.68 (t, *J* = 5.0 Hz, 1H), 7.01 (s, 1H), 6.31 (d, *J* = 8.5 Hz, 1H), 4.72 (t, *J* = 8.2 Hz, 1H), 4.41 (dd, *J* = 6.3, 3.9 Hz, 1H), 4.30 (dd, *J* = 8.7, 2.0 Hz, 1H), 4.22 – 4.01 (m, 3H), 2.93 – 2.77 (m, 2H), 2.37 – 2.26 (m, 1H), 2.24 – 2.09 (m, 2H), 2.03 (s, 4H), 1.93 – 1.82 (m, 2H), 1.71 – 1.33 (m, 11H), 1.30 (s, 9H), 1.28 (s, 3H), 1.21 (d, *J* = 10.9 Hz, 1H), 0.94 (d, *J* = 6.4 Hz, 3H), 0.84 (s, 3H)
^{13}C NMR (101 MHz, Chloroform-*d*) δ 171.8, 170.0, 169.5, 169.3, 86.4, 78.2, 75.6, 66.4, 57.9, 56.3, 51.5, 44.2, 42.9, 39.6, 38.3, 35.5, 29.2, 29.0, 28.7, 28.3, 27.2, 26.5, 25.2, 25.0 (CHB, broad signal), 24.9, 24.2, 23.6, 17.4
UPLC-MS (ESI) calculated for $C_{30}H_{51}BN_4O_7$ $[M+H]^+$ 591.39, found 591.79

10e: synthesized from **8b** and **9d.** The same synthetic modification was made as for **10d**. Yield: 67 mg (36%) as a solid compound.
^{1}H NMR (400 MHz, Chloroform-*d*) δ 7.57 (t, *J* = 5.2 Hz, 1H), 7.54 – 7.46 (m, 1H), 7.39 (s, 1H), 7.23 (d, *J* = 8.6 Hz, 2H), 6.86 (d, *J* = 8.6 Hz, 2H), 6.21 (d, *J* = 8.2 Hz, 1H), 4.57 (t, *J* = 8.0 Hz, 1H), 4.47 – 4.38 (m, 2H), 4.38 – 4.32 (m, 1H), 4.24 (dd, *J* = 8.8, 2.1 Hz, 1H), 4.20 – 3.94 (m, 3H), 3.80 (s,

3H), 3.53 (t, *J* = 6.1 Hz, 2H), 3.15 – 3.04 (m, 1H), 2.37 – 2.23 (m, 1H), 2.22 – 2.08 (m, 2H), 2.07 – 1.96 (m, 4H), 1.95 – 1.72 (m, 4H), 1.71 – 1.45 (m, 6H), 1.41 – 1.19 (m, 18H), 0.96 (d, *J* = 6.4 Hz, 3H), 0.83 (s, 3H).
^{13}C NMR (101 MHz Chloroform-*d*) δ 171.7, 170.1, 170.0, 169.4, 159.3, 130.6, 129.4, 113.9, 85.2, 77.5, 75.6, 72.6, 68.4, 66.3, 58.0, 56.5, 55.4, 51.8, 43.8, 41.6, 39.8, 38.3, 36.9 (<u>C</u>HB, broad signal), 36.0, 30.9, 29.3, 28.9, 28.8, 28.3, 27.3, 26.5, 25.2, 24.9, 24.3, 23.5, 17.6
HR-MS (ESI/TOF) calculated for $C_{40}H_{64}BN_4O_9$ [M+H]$^+$ 755.4766, found 755.4771

10f: synthesized from **8b** and **9e**. The same modification was made as for **10d** Yield: 34 mg (22%) as a solid compound.
^{1}H NMR (400 MHz, Chloroform-*d*) δ 7.92 (d, *J* = 6.3 Hz, 1H), 7.79 (d, *J* = 4.0 Hz, 1H), 7.61 (t, *J* = 5.2 Hz, 1H), 7.22 (d, *J* = 8.6 Hz, 2H), 6.85 (d, *J* = 8.6 Hz, 2H), 6.35 (d, *J* = 8.5 Hz, 1H), 4.73 (t, *J* = 8.1 Hz, 1H), 4.45 – 4.35 (m, 3H), 4.32 – 4.21 (m, 2H), 4.09 – 3.98 (m, 2H), 3.79 (s, 3H), 3.43 (t, *J* = 5.7 Hz, 2H), 2.94 – 2.86 (m, 1H), 2.36 – 2.26 (m, 1H), 2.19 – 2.07 (m, 2H), 2.02 – 1.96 (m, 4H), 1.89 – 1.78 (m, 2H), 1.76 – 1.41 (m, 10H), 1.40 – 1.22 (m, 18H), 0.92 (d, *J* = 6.3 Hz, 3H), 0.84 (s, 3H)
^{13}C NMR (101 MHz, Chloroform-*d*) δ 171.8, 170.2, 170.0, 169.3, 159.2, 130.79, 129.3, 113.8, 85.0, 77.4, 75.6, 72.6, 70.1, 66.5, 58.0, 56.1, 55.4, 51.8, 44.2, 41.5, 39.9, 39.0 (<u>C</u>HB, broad signal), 38.3, 36.0, 29.2, 28.9, 28.8, 28.3, 28.2, 27.7, 27.4, 26.6, 25.2, 24.9, 24.3, 23.5, 17.4
HR-MS (ESI/TOF) calculated for $C_{41}H_{65}BN_4O_9$ [M+H]$^+$ 769.4923, found 769.4936

10g: synthesized from **8b** and **9f**. The same modification was made as for **10d**. Yield: 94 mg (52%) as a solid compound.
^{1}H NMR (400 MHz, Chloroform-*d*) δ 7.98 (d, *J* = 3.9 Hz, 1H), 7.91 (d, *J* = 6.2 Hz, 1H), 7.62 (t, *J* = 5.2 Hz, 1H), 6.44 (d, *J* = 8.5 Hz, 1H), 4.74 (t, *J* = 8.1 Hz, 1H), 4.39 (dd, *J* = 6.2, 4.1 Hz, 1H), 4.32 (dd, *J* = 17.4, 6.0 Hz, 1H), 4.25 (dd, *J* = 8.8, 2.1 Hz, 1H), 4.14 – 3.97 (m, 2H), 2.90 – 2.80 (m, 1H), 2.44 – 2.24 (m, 3H), 2.19 – 2.07 (m, 2H), 2.04 – 1.96 (m, 4H), 1.95 – 1.69 (m, 5H), 1.68 – 1.51 (m, 5H), 1.39 (d, *J* = 13.5 Hz, 15H), 1.28 (s, 9H), 1.26 (s, 3H), 0.93 (d, *J* = 6.3 Hz, 3H), 0.84 (s, 3H)
^{13}C NMR (101 MHz, Chloroform-*d*) δ 173.1, 171.8, 170.6, 170.1, 169.3, 84.9, 80.2, 77.4, 75.6, 66.5, 58.1, 56.1, 51.9, 44.3, 41.3, 39.9, 38.8 (<u>C</u>HB, broad signal), 38.3, 36.1, 33.6, 29.2, 28.89, 28.85, 28.3, 28.2, 27.4, 26.9, 26.6, 25.2, 24.9, 24.3, 23.5, 17.5
HR-MS (ESI/TOF) calculated for $C_{37}H_{64}BN_4O_9$ [M+H]$^+$ 719.4766, found 719.4765

10h: synthesized from **8b** and **9g**. Yield: 117 mg (70%) as a solid compound.
^{1}H NMR (300 MHz, Chloroform-*d*) δ 8.20 – 7.99 (m, 1H), 7.97 – 7.78 (m, 1H), 7.77 – 7.55 (m, 1H), 7.36 – 7.11 (m, 5H, overlaps with $CHCl_3$ residue signal), 6.37 – 6.22 (m, 1H), 4.82 – 4.65 (m, 1H), 4.54 – 4.32 (m, 2H), 4.31 – 3.96 (m, 4H), 2.30 – 1.92 (m, 4H), 1.92 – 1.82 (m, 3H), 1.80 – 1.43 (m, 8H), 1.41 – 1.09 (m, 18H), 0.98 – 0.87 (m, 3H), 0.80 (s, 3H)
^{13}C NMR (101 MHz, Chloroform-*d*) δ 171.9, 171.5, 170.3, 169.6, 140.3, 128.4, 126.6, 126.2, 84.9, 77.5, 75.7, 66.6, 58.0, 56.2, 52.0, 45.8 (<u>C</u>HB, broad signal), 44.2, 41.1, 39.8, 38.3, 36.0, 29.2, 28.9, 28.7, 28.2, 27.3, 26.4, 25.2, 24.9, 24.2, 23.4, 17.4
HR-MS (ESI/TOF) calculated for $C_{36}H_{56}BN_4O_7$ [M+H]$^+$ 667.4242, found 667.4243

10i: synthesized from **8c** and **9a.** Yield: 63 mg (48%) as a solid compound.
^{1}H NMR (400 MHz, Methanol-*d*$_4$) δ 4.26 – 4.08 (m, 4H), 3.93 (dd, *J* = 17.6, 1.0 Hz, 1H), 2.65 (q, *J* = 7.2 Hz, 1H), 2.39 – 2.29 (m, 1H), 2.28 – 2.16 (m, 1H), 2.17 – 2.08 (m, 1H), 1.98 (s, 3H), 1.95 (t, *J* = 5.5 Hz, 1H), 1.89 – 1.75 (m, 3H), 1.74 – 1.51 (m, 5H), 1.44 (d, *J* = 10.3 Hz, 1H), 1.37 (d, *J* = 7.2 Hz, 3H), 1.36 (s, 3H), 1.34 – 1.30 (m, 2H, overlaps), 1.28 (s, 3H), 1.17 (d, *J* = 7.3 Hz, 3H), 0.87 (s, 3H).
^{13}C NMR (101 MHz, Methanol-*d*$_4$) δ 175.9, 175.5, 174.8, 173.5, 84.3, 77.3, 58.92, 53.6, 51.1, 43.1, 41.4, 40.2, 39.8 (<u>C</u>HB, broad signal), 39.2, 37.7, 30.3, 30.2, 29.6, 27.8, 27.5, 26.2, 25.9, 24.5, 22.3, 17.0, 16.4.
HR-MS (ESI/TOF) calculated for $C_{26}H_{29}BN_4O_6$ [M+H]$^+$ 519.3354, found 519.3368

10j: synthesized from **8d** and **9a.** Yield: 43 mg (50%) as a solid compound.

^{1}H NMR (400 MHz, Methanol-d_4) δ 4.24 – 4.12 (m, 3H), 3.97 (d, *J* = 8.0 Hz, 1H), 3.95 (dd, *J* = 17.5, 1.0 Hz, 1H), 2.65 (q, *J* = 7.3 Hz, 1H), 2.40 – 2.28 (m, 1H), 2.29 – 2.15 (m, 1H), 2.17 – 2.09 (m, 1H), 2.09 – 2.02 (m, 1H), 1.97 (s, 3H), 1.94 (t, *J* = 5.6 Hz, 1H), 1.89 – 1.75 (m, 3H), 1.72 – 1.51 (m, 5H), 1.44 (d, *J* = 10.3 Hz, 1H), 1.35 (s, 3H), 1.40 – 1.27 (m, 2H), 1.28 (s, 3H), 1.17 (d, *J* = 7.3 Hz, 3H), 1.01 (d, *J* = 6.8 Hz, 3H), 0.96 (d, *J* = 6.8 Hz, 3H), 0.87 (s, 3H).
^{13}C NMR (101 MHz, Methanol-d_4) δ 175.8, 175.0, 174.3, 173.3, 84.3, 77.3, 61.3, 58.9, 53.6, 43.0, 41.4, 40.0 (overlaps with C̲HB, broad signal), 39.2, 37.7, 31.2, 30.4, 30.2, 29.6, 27.8, 27.5, 26.3, 26.0, 24.5, 22.3, 19.7, 19.2, 16.4.
UPLC-MS (ESI) calculated for $C_{28}H_{48}BN_4O_6$ $[M+H]^+$ 547.52, found 547.76

To a solution of **10a** (50 mg, 0.08 mmol) in DCM (5 mL), BBr_3 (180 µL, 1 M solution in DCM) was added dropwise at -78°C. The mixture was stirred while the temperature was slowly warmed up to ambient temperature. After 2 h, the reaction was quenched with water (10 mL) and the mixture was extracted with Et_2O (3×10 mL). The aqueous solution was concentrated in vacuo affording **3a** (EP_530) (29 mg, 85%) as a yellow solid.
^{1}H NMR (400 MHz, Methanol-d_4) δ 4.31 – 4.07 (m, 5H), 2.82 – 2.71 (m, 1H), 2.05 (s, 3H), 1.94 – 1.83 (m, 1H), 1.61 – 1.50 (m, 1H), 1.28 – 1.22 (m, 1H), 1.20 (d, *J* = 6.3 Hz, 3H), 1.12 (d, *J* = 7.2 Hz, 3H), 0.96 (d, *J* = 6.8 Hz, 3H), 0.92 (t, *J* = 7.4 Hz, 3H).
^{13}C NMR (101 MHz, Methanol-d_4) δ 176.9, 174.3, 174.2, 173.1, 68.1, 60.5, 60.0, 39.7, 37.6, 26.1, 22.2, 19.91, 15.9, 15.7, 11.4.
HR-MS (ESI/TOF) calculated for $C_{16}H_{30}BN_4O_6$ $[M+H]^+$ 385.2258, found 385.2263

A solution of **10b** (73 mg, 0.12 mmol) in MeCN/*n*-hexane (1:1, 8 mL) was treated with isobutylboronic acid (37 mg, 0.36 mmol, 3 equiv) and 1 M HCl (500 µL). After 18 h at room temperature the MeCN and *n*-hexane was separated and MeCN phase was washed with *n*-hexane (3x10 mL) and then the *n*-hexane layer was washed with MeCN (3x10 mL). The combined MeCN phases were evaporated in vacuo. The crude product was purified by reversed phase column chromatography to give **int-b** as a white solid (36 mg, 66%).
^{1}H NMR (400 MHz, Methanol-d_4) δ 4.34 (d, *J* = 3.9 Hz, 1H), 4.24 (d, *J* = 17.6 Hz, 1H), 4.18 (d, *J* = 9.1 Hz, 1H), 4.15 – 4.10 (m, 1H), 4.06 (dd, *J* = 17.7, 1.7 Hz, 1H), 2.69 (q, *J* = 7.2 Hz, 1H), 2.24 (h, *J* = 8.9 Hz, 1H), 2.00 (s, 3H), 1.88 – 1.77 (m, 1H), 1.76 – 1.50 (m, 5H), 1.44 – 1.27 (m, 2H), 1.25 (s, 9H), 1.14 (d, *J* = 6.4 Hz, 3H), 1.10 (d, *J* = 7.2 Hz, 3H)
^{13}C NMR (101 MHz, Methanol-d_4) δ 176.3, 174.4, 173.6, 172.1, 76.5, 68.3, 59.6, 59.3, 42.6, 41.8 (C̲HB, broad signal), 39.7, 30.4, 30.3, 28.5, 26.2, 25.9, 22.3, 19.2, 16.0
HR-MS (ESI/TOF) calculated for $C_{21}H_{40}BN_4O_7$ $[M+H-H_2O]^+$ 453.2884, found 453.2880
A solution of **int-b** (17 mg, 0.038 mmol) in dry DCM (2 mL) was treated with TFA (500 µL). The reaction was stirred to completion (UPLC-MS control). Toluene (5 mL) was added to the reaction mixture and then the solvents were removed. Crude mixture was treated with Et_2O (3x5 mL, the precipitate was separated by centrifugation after each addition) to give the product **3b** (EP_784) as a white solid (11 mg, 74%).
^{1}H NMR (400 MHz, Methanol-d_4) δ 4.26 (d, *J* = 4.5 Hz, 1H), 4.24 – 4.02 (m, 4H), 2.67 (q, *J* = 7.1 Hz, 1H), 2.26 (h, *J* = 8.7 Hz, 1H), 2.00 (s, 3H), 1.88 – 1.77 (m, 1H), 1.76 – 1.51 (m, 5H), 1.44 – 1.28 (m, 2H), 1.20 (d, *J* = 6.4 Hz, 3H), 1.10 (d, *J* = 7.2 Hz, 3H)
^{13}C NMR (101 MHz, Methanol-d_4) δ 176.3, 174.9, 173.7, 173.1, 68.2, 60.5, 59.3, 42.7, 41.9 (C̲HB, broad signal), 39.8, 30.3, 26.3, 26.0, 22.3, 19.9, 15.9
HR-MS (ESI/TOF) calculated for $C_{17}H_{30}BN_4O_6$ $[M+H-H_2O]^+$ 397.2258, found 397.2265

The same general method was used for synthesis of **3c-j**

Yield: **int-c** (EP_785) (39 mg, 64%) was isolated as a solid compound.
^{1}H NMR (400 MHz, Methanol-d_4) δ 4.36 – 4.28 (m, 1H), 4.25 – 4.06 (m, 4H), 2.76 – 2.64 (m, 1H), 2.26 (h, *J* = 8.9 Hz, 1H), 2.01 (s, 3H), 1.89 – 1.78 (m, 1H), 1.77 – 1.52 (m, 5H), 1.46 – 1.28 (m, 2H), 1.23 (s, 9H), 1.14 (d, *J* = 6.3 Hz, 3H), 1.12 – 1.07 (m, 3H)
^{13}C NMR (101 MHz, Methanol-d_4) δ 176.1, 174.6, 173.7, 172.5, 76.1, 68.2, 60.0, 59.4, 42.5, 41.8 (C̲HB, broad signal), 39.8, 30.4, 28.6, 26.2, 25.9, 22.3, 19.6, 16.0
HR-MS (ESI/TOF) calculated for $C_{21}H_{40}BN_4O_7$ $[M+H-H_2O]^+$ 453.2884, found 453.2889

Yield: **3c** (25 mg, 73%) as a solid compound.
^{1}H NMR (400 MHz, Methanol-d_4) δ 4.27 – 4.03 (m, 5H), 2.69 (q, *J* = 7.4 Hz, 1H), 2.26 (h, *J* = 9.0 Hz, 1H), 2.00 (d, *J* = 0.8 Hz, 3H), 1.89 – 1.77 (m, 1H), 1.76 – 1.49 (m, 5H), 1.46 – 1.26 (m, 2H), 1.20 (d, *J* = 6.3 Hz, 3H), 1.09 (d, *J* = 7.1 Hz, 3H).
^{13}C NMR (101 MHz, Methanol-d_4) δ 176.3, 174.9, 173.7, 173.0, 68.2, 60.6, 59.4, 42.7, 41.8 ($\underline{C}$HB, broad signal), 39.8, 30.4, 30.3, 26.2, 25.9, 22.3, 19.9, 15.9
HR-MS (ESI/TOF) calculated for $C_{17}H_{30}BN_4O_6$ $[M+H-H_2O]^+$ 397.2258, found 397.2264

int-d was not fully characterized, since partial cleavage of protecting group was observed. The crude mixture was used in the next step.
UPLC-MS (ESI) calculated for $C_{20}H_{36}BN_4O_6$ $[M+H-H_2O]^+$ 439.27, found 439.61.
Yield: **3d** (EP_863) (23 mg, 75% in two steps) as a solid compound.
^{1}H NMR (400 MHz, Methanol-d_4) δ 4.30 – 3.96 (m, 5H), 2.38 (s, 2H), 2.26 (h, *J* = 8.8 Hz, 1H), 2.00 (s, 3H), 1.90 – 1.77 (m, 1H), 1.76 – 1.52 (m, 5H), 1.47 – 1.28 (m, 2H), 1.19 (d, *J* = 6.3 Hz, 3H).
^{13}C NMR (101 MHz, Methanol-d_4) δ 176.6, 174.9, 173.7, 173.1, 68.1, 60.5, 59.3, 42.8, 40.4, 31.5 ($\underline{C}$HB, broad signal), 30.4, 30.3, 26.3, 26.0, 22.3, 19.9
UPLC-MS (ESI) calculated for $C_{16}H_{28}BN_4O_6$ $[M-H_2O+H]^+$ 383.21, found 383.51.

int-e was not fully characterized, since partial cleavage of protecting group was observed. The crude mixture was used in the next step.
UPLC-MS (ESI) calculated for $C_{20}H_{36}BN_4O_6$ $[M+H-H_2O]^+$ 439.27, found 439.61.
Yield: **3e** (EP_837) (35 mg, 93% in two steps) as a solid compound.
^{1}H NMR (400 MHz, Methanol-d_4) δ 4.33 – 4.08 (m, 5H), 3.91 – 3.77 (m, 1H), 3.57 – 3.45 (m, 1H), 2.85 (d, *J* = 6.2 Hz, 1H), 2.26 (h, *J* = 8.9 Hz, 1H), 2.00 (s, 3H), 1.93 – 1.51 (m, 8H), 1.45 – 1.27 (m, 2H), 1.20 (d, *J* = 6.4 Hz, 3H)
^{13}C NMR (101 MHz, Methanol-d_4) δ 178.2, 174.9, 173.7, 173.1, 68.1, 64.6, 60.4, 59.3, 43.5 ($\underline{C}$HB, broad signal), 42.7, 39.6, 34.4, 30.4, 30.3, 26.3, 26.0, 22.3, 19.9
HR-MS (ESI/TOF) calculated for $C_{18}H_{31}BN_4O_7Na$ $[M+Na]^+$ 449.2183, found 449.2170

int-f was not fully characterized, since partial cleavage of protecting group was observed. The crude mixture was used in the next step.
UPLC-MS (ESI) calculated for $C_{31}H_{50}BN_4O_8$ $[M-H_2O+H]^+$ 617.37, found 617.72.
Yield: **3f** (EP_861) as a white solid (15 mg, 81% in two steps).
^{1}H NMR (400 MHz, Methanol-d_4) δ 4.28 (d, *J* = 4.3 Hz, 1H), 4.24 – 4.13 (m, 2H), 4.15 – 3.99 (m, 2H), 3.89 – 3.71 (m, 2H), 2.63 (t, *J* = 5.5 Hz, 1H), 2.26 (h, *J* = 8.6 Hz, 1H), 2.00 (s, 3H), 1.89 – 1.28 (m, 12H), 1.22 – 1.16 (m, 3H)
^{13}C NMR (101 MHz, Methanol-d_4) δ 176.0, 174.8, 173.7, 173.0, 68.2, 64.6, 60.4, 59.3, 42.7, 40.7 ($\underline{C}$HB, broad signal), 40.6, 30.4, 30.3, 28.8, 26.3, 26.0, 24.7, 22.3, 19.9
UPLC-MS (ESI) calculated for $C_{19}H_{32}BN_4O_6$ $[M+H-H_2O]^+$ 423.24, found 423.55.

int-g (65 mg, 69%) was isolated as white solid.
^{1}H NMR (400 MHz, Methanol-d_4) δ 4.35 – 4.24 (m, 2H), 4.23 – 4.09 (m, 3H), 2.88 (t, *J* = 4.2 Hz, 1H), 2.34 – 2.15 (m, 3H), 2.01 (s, 3H), 1.88 – 1.77 (m, 3H), 1.76 – 1.52 (m, 5H), 1.45 – 1.28 (m, 2H), 1.24 (s, 9H), 1.14 (d, *J* = 6.3 Hz, 3H)
^{13}C NMR (101 MHz, Methanol-d_4) δ 179.8, 176.6, 173.8, 76.4, 68.2, 59.7, 59.4, 42.7 ($\underline{C}$HB, broad signal), 42.5, 39.5, 30.4, 30.3, 29.2, 28.5, 26.2, 26.0, 25.5, 22.3, 19.5
UPLC-MS (ESI) calculated for $C_{23}H_{40}BN_4O_8$ $[M+H-H_2O]^+$ 511.29, found 511.72
Yield: **3g** (EP_852) was isolated as a white solid (47 mg, 96%).
^{1}H NMR (400 MHz, Methanol-d_4) δ 4.30 – 4.01 (m, 5H), 2.56 (t, *J* = 7.3 Hz, 1H), 2.47 (t, *J* = 7.6 Hz, 2H), 2.26 (h, *J* = 8.8 Hz, 1H), 2.00 (s, 3H), 1.90 – 1.53 (m, 8H), 1.44 – 1.26 (m, 2H), 1.20 (d, *J* = 6.3 Hz, 3H)
^{13}C NMR (101 MHz, Methanol-d_4) δ 176.8, 175.9, 174.9, 173.6, 173.2, 68.0, 61.1, 58.9, 45.6 ($\underline{C}$HB, broad signal), 42.9, 39.9, 32.9, 30.3, 30.2, 27.2, 26.3, 25.9, 22.3, 19.9
UPLC-MS (ESI) calculated for $C_{19}H_{32}BN_4O_8$ $[M+H]^+$ 455.23, found 455.61

int-h (40.6 mg, 45%) was isolated as white solid.

^{1}H NMR (400 MHz, Methanol-d_4) δ 7.32 – 7.20 (m, 2H), 7.19 – 7.08 (m, 3H), 4.48 – 4.16 (m, 4H), 4.15 – 4.07 (m, 1H), 3.81 – 3.74 (m, 1H), 2.29 – 2.12 (m, 1H), 1.99 (s, 3H), 1.86 – 1.75 (m, 1H), 1.74 – 1.49 (m, 5H), 1.42 – 1.28 (m, 2H), 1.27 – 1.17 (m, 9H), 1.18 – 1.06 (m, 3H)
^{13}C NMR (101 MHz, Methanol-d_4) δ 178.2, 174.5, 173.6, 172.3, 141.9, 129.1, 127.3, 126.8, 76.5, 68.3, 59.6, 59.2, 53.7 (CHB, broad signal), 42.6, 39.6, 30.4, 30.3, 28.4, 26.2, 25.9, 22.3, 19.1
HR-MS (ESI/TOF) calculated for $C_{26}H_{42}BN_4O_7$ $[M+H-H_2O]^+$ 515.3041, found 515.3051
When protecting group cleavage reaction with TFA was performed, product **3h'** was observed as TFA ester (47 mg, 96%):
^{1}H NMR (400 MHz, Methanol-d_4) δ 7.29 – 7.05 (m, 5H), 4.52 – 4.07 (m, 5H), 3.78 (s, 1H), 2.28 – 2.15 (m, 1H), 2.03 – 1.91 (m, 3H), 1.87 – 1.47 (m, 7H), 1.44 – 1.24 (m, 1H), 1.22 – 1.14 (m, 3H)
^{13}C NMR (101 MHz, Methanol-d_4) δ 178.2, 174.8, 173.8, 173.2, 158.5 (q, J = 41.2 Hz), 156.6 (q, J = 41.7 Hz) 142.1, 129.1, 127.0, 126.6, 116.3 (q, 286.3 Hz), 115.9 (q, J = 286.5 Hz). 68.2, 60.3, 59.3, 53.8 (CHB, broad signal), 42.6, 39.7, 30.3, 26.3, 26.0, 22.3, 19.9
^{19}F NMR (376 MHz, Methanol-d_4) δ -74.93, -75.15, -76.27, -76.55
HR-MS (ESI/TOF) calculated for $C_{26}H_{31}BN_4O_9F_6Na$ $[M+Na-H_2O]^+$ 691.1986, found 691.2001
The product **3h'** was transesterificated with (+)-pinanediol and cleaved again with isobutylboronic acid according to the same method used before to give **3h** (EP_842) (19 mg, 94%) as a white solid compound.
^{1}H NMR (400 MHz, Methanol-d_4) δ 7.30 – 7.21 (m, 2H), 7.17 – 7.09 (m, 3H), 4.43 – 4.11 (m, 5H), 3.81 – 3.75 (m, 1H), 2.31 – 2.16 (m, 1H), 1.97 (s, 3H), 1.85 – 1.52 (m, 6H), 1.47 – 1.28 (m, 2H), 1.22 – 1.15 (m, 3H)
^{13}C NMR (101 MHz, Methanol-d_4) δ 178.2, 174.8, 173.8, 173.2, 142.1, 129.1, 127.0, 126.6, 68.2, 60.3, 59.3, 53.8 (CHB, broad signal), 42.6, 39.7, 30.34, 30.32, 26.3, 26.0, 22.3, 19.9
HR-MS (ESI/TOF) calculated for $C_{22}H_{32}BN_4O_6$ $[M+H-H_2O]^+$ 459.2415, found 459.2418

3i (EP_1199) (30 mg, 75%) was isolated as white solid. For reaction mixture methanol was used instead of MeCN.
^{1}H NMR (400 MHz, Methanol-d_4) δ 4.26 – 4.16 (m, 2H), 4.12 (d, J = 9.1 Hz, 1H), 3.98 (dd, J = 17.8, 1.0 Hz, 1H), 2.66 (q, J = 7.2 Hz, 1H), 2.20 (h, J = 8.9 Hz, 1H), 1.98 (s, 3H), 1.87 – 1.77 (m, 1H), 1.75 – 1.63 (m, 3H), 1.63 – 1.51 (m, 2H), 1.38 (d, J = 7.1 Hz, 3H), 1.43 – 1.24 (m, 2H), 1.12 (d, J = 7.2 Hz, 3H).
^{13}C NMR (101 MHz, Methanol-d_4) δ 176.3, 175.6, 174.8, 173.5, 58.9, 51.1, 43.2, 41.8 (broad), 39.8, 30.3, 30.2, 26.2, 25.8, 22.3, 17.0, 16.0.
HR-MS (ESI/TOF) calculated for $C_{16}H_{29}BN_4O_6Na$ $[M+Na]^+$ 407.2078, found 407.2089

3j (EP_1200) (25 mg, 77%) was isolated as white solid. For reaction mixture methanol was used instead of MeCN.
^{1}H NMR (400 MHz, Methanol-d_4) δ 4.26 – 4.15 (m, 2H), 4.03 – 3.93 (m, 2H), 2.66 (q, J = 7.0 Hz, 1H), 2.29 – 2.13 (m, 1H), 2.11 – 1.98 (m, 1H), 1.97 (s, 3H), 1.86 – 1.76 (m, 1H), 1.73 – 1.49 (m, 5H), 1.42 – 1.25 (m, 2H), 1.12 (d, J = 7.2 Hz, 3H), 1.01 (d, J = 6.7 Hz, 3H), 0.97 (d, J = 6.8 Hz, 3H).
^{13}C NMR (101 MHz, Methanol-d_4) δ 176.3, 175.0, 174.5, 173.3, 61.3, 58.9, 43.1, 41.9 (broad), 39.6, 31.2, 30.4, 30.2, 26.3, 25.9, 22.3, 19.6, 19.2, 15.9.
HR-MS (ESI/TOF) calcd for $C_{16}H_{29}BN_4O_6Na$ $[M+Na]^+$ 435.2391, found 435.2411

(+)-Pinanediol Methylboronate **12a**.
To the solution of methylboronic acid pinacol ester **11a** (2 mL, 1.76 g, 12.4 mmol, 1.0 equiv) in THF (40 mL) was added (+)-pinanediol (3.17 g, 18.6 mmol, 1.5 equiv). The mixture was stirred for 15 hours at room temperature. Then the solution was evaporated and crude mixture was purified by flash chromatography on silica gel eluting with Hexane:EtOAc (20:1) – Hexane:EtOAc (8:1) to provide **12a** (2.32 g, 96%) as a colorless oil.
^{1}H NMR (300 MHz, Chloroform-d) δ 4.25 (dd, J = 8.7, 1.9 Hz, 1H), 2.40 – 2.25 (m, 1H), 2.26 – 2.16 (m, 1H), 2.03 (dd, J = 6.1, 5.0 Hz, 1H), 1.95 – 1.80 (m, 2H), 1.38 (s, 3H), 1.28 (s, 3H), 1.12 (d, J = 10.9 Hz, 1H), 0.84 (s, 3H), 0.28 (s, 3H).
GC/MS m/z = 194.1
Analytical data are in accordance with those reported in the literature[4].

(+)-Pinanediol (1*S*)-(1-chloroethyl)boronate **13a**.

Matteson homologation was used to synthesise α-chlorinated boronates.[3] A stirred solution of anhydrous dichloromethane (3.8 mL, 59.3 mmol, 5.0 equiv) in anhydrous tetrahydrofuran (30 mL) was cooled in liquid nitrogen/ethanol bath to −100°C and treated with *n*-buthyl lithium 2.5 M (7.2 mL, 18.0 mmol, 1.5 equiv) over a period of 30 min (under argon). After 20 min to the resulting mixture a solution of pinanediol alkylboronate **12a** (2.32 g, 12.0 mmol, 1.0 equiv) in anhydrous tetrahydrofuran (15 mL) was added dropwise and the reaction mixture was stirred for 30 min at −100°C. Then $ZnCl_2$ 1 M (21.5 mL, 21.5 mmol, 1.8 equiv) was added slowly. The cooling bath was removed and the reaction was allowed to warm to room temperature. After 2 h diethyl ether was added to the reaction mixture and the suspension obtained was washed with a saturated ammonium chloride solution. The solvent was evaporated and the oily residue was dissolved in diethyl ether, washed with brine and organic phase was dried over Na_2SO_4, filtered and evaporated in vacuo. The residue was purified by flash chromatography on silica gel eluting with Hexane:EtOAc (20:1) – Hexane:EtOAc (8:1) to provide **13a** (2.39 g, 82%) as a colorless oil.

^{1}H NMR (400 MHz, Chloroform-*d*) δ 4.36 (dd, *J* = 8.8, 1.8 Hz, 1H), 3.57 (q, *J* = 7.6 Hz, 1H), 2.40 – 2.31 (m, 1H), 2.29 – 2.21 (m, 1H), 2.11 – 2.07 (m, 1H), 1.96 – 1.86 (m, 2H), 1.57 (d, *J* = 7.5 Hz, 3H), 1.42 (s, 3H), 1.29 (s, 3H), 1.17 (d, *J* = 11.1 Hz, 1H), 0.84 (s, 3H).

^{13}C NMR (101 MHz, Chloroform-*d*) δ 86.9, 78.7, 51.3, 39.5, 38.4, 35.4, 28.6, 27.2, 26.4, 24.1, 20.7.

GC/MS m/z = 242.1

Analytical data are in accordance with those reported in the literature[5].

(+)-Pinanediol (1*R*)-(1-aminoethyl)boronate hydrochloride **9a**.

To the solution of α-chloroboronic acid ester **13a** (2.39 g, 9.8 mmol, 1 equiv) in anhydrous tetrahydrofuran (30 mL) lithium *bis*(trimethylsilyl)amide 1 M (9.8 mL, 9.8 mmol, 1 equiv) was slowly added at −78° C. The mixture was allowed to warm up and stirred for 3 h at room temperature. The solvent was removed in vacuo and hexane (50 mL) was added to the residue. The inorganic precipitates were filtered off through a pad of Celite, and then washed with additional amount of hexane, and filtrate was submitted directly to the next step.

GC/MS m/z = 352.2 (M−Me), 294.2 (M−$SiMe_3$)

To the solution of *bis*-silyl intermediate in hexane (~60 mL) HCl/dioxane 4 M (8.2 mL, 32.8 mmol, 3.3 equiv) was slowly added at 0°C. The reaction was allowed to warm to room temperature and stirred overnight. Solid was filtered, washed with hexane and dried in the air to give a product **9a** as a white powder (1.01 g, 40%)

^{1}H NMR (400 MHz, Methanol-d_4) δ 4.48 (dd, *J* = 8.9, 1.9 Hz, 1H), 2.96 (q, *J* = 7.8 Hz, 1H), 2.48 – 2.36 (m, 1H), 2.35 – 2.24 (m, 1H), 2.07 (t, *J* = 5.5 Hz, 1H), 1.98 – 1.84 (m, 2H), 1.45 (s, 3H), 1.35 (d, *J* = 7.7 Hz, 3H), 1.32 (s, 3H), 1.16 (d, *J* = 11.0 Hz, 1H), 0.88 (s, 3H).

^{13}C NMR (101 MHz, Methanol-d_4) δ 88.9, 80.2, 52.5, 40.7, 39.3, 36.0, 33.8 (CHB, broad signal), 28.8, 27.4, 27.2, 24.2, 14.9.

(−)-Pinanediol Methylboronate **12b**.

The same method was used as for **12a**: methylboronic acid pinacol ester **11a** (2 mL, 1.76 g, 12.4 mmol, 1.0 equiv), (−)-pinanediol (3.17 g, 18.6 mmol, 1.5 equiv), THF (40 mL). The mixture was purified by flash chromatography on silica gel eluting with Hexane:EtOAc (20:1) – Hexane:EtOAc (8:1) to provide **12b** (1.89 g, 79%) as a colorless oil.

^{1}H NMR (300 MHz, Chloroform-*d*) δ 4.25 (dd, *J* = 8.7, 1.9 Hz, 1H), 2.39 – 2.27 (m, 1H), 2.27 – 2.16 (m, 1H), 2.04 (dd, *J* = 6.1, 5.0 Hz, 1H), 1.95 – 1.80 (m, 2H), 1.39 (s, 3H), 1.29 (s, 3H), 1.13 (d, *J* = 10.8 Hz, 1H), 0.84 (s, 3H), 0.28 (s, 3H).

GC/MS m/z = 194.1

Analytical data are in accordance with those reported in the literature[3].

(−)-Pinanediol (1*R*)-(1-chloroethyl)boronate **13b**.

The same method was used as for **13a**: anhydrous dichloromethane (3 mL, 46.8 mmol, 5.0 equiv) in anhydrous tetrahydrofuran (30 mL), *n*-buthyl lithium 2.2 M (6.25 mL, 13.8 mmol, 1.5 equiv), pinanediol alkylboronate **13a** (1.78 g, 9.2 mmol, 1.0 equiv) in anhydrous tetrahydrofuran (15 mL), $ZnCl_2$ 1 M (16.5 mL, 16.5 mmol, 1.8 equiv). The residue was purified by flash chromatography on

silica gel eluting with Hexane:EtOAc (20:1) – Hexane:EtOAc (8:1) to provide **13b** (1.89 g, 85%) as a colorless oil.
^{1}H NMR (400 MHz, Chloroform-*d*) δ 4.36 (dd, *J* = 8.8, 1.9 Hz, 1H), 3.57 (q, *J* = 7.6 Hz, 1H), 2.41 – 2.31 (m, 1H), 2.29 – 2.20 (m, 1H), 2.11 – 2.06 (m, 1H), 1.97 – 1.85 (m, 2H), 1.57 (d, *J* = 7.6 Hz, 3H), 1.42 (s, 3H), 1.29 (s, 3H), 1.17 (d, *J* = 11.1 Hz, 1H), 0.84 (s, 3H).
^{13}C NMR (101 MHz, Chloroform-*d*) δ 86.9, 78.7, 51.3, 39.5, 38.4, 35.4, 28.6, 27.2, 26.4, 24.1, 20.7.
GC/MS m/z = 242.1
Analytical data are in accordance with those reported in the literature[3,6].

<u>(−)-Pinanediol (1*S*)-(1-aminoethyl)boronate hydrochloride **9b**.</u>
The same method was used as for **9a**: α-chloroboronic ester **13b** (1.88 g, 7.74 mmol, 1.0 equiv) in anhydrous tetrahydrofuran (30 mL), lithium *bis*(trimethylsilyl)amide 1 M (7.75 mL, 7.75 mmol, 1.0 equiv).
GC/MS m/z = 352.2 (M−Me), 294.2 (M−SiMe$_3$)
Then HCl/dioxane 4 M (6.4 mL, 25.6 mmol, 3.3 equiv). **9b** was isolated as a white powder (631 mg, 31%).
^{1}H NMR (400 MHz, Methanol-*d*$_4$) δ 4.48 (dd, *J* = 8.9, 1.9 Hz, 1H), 2.96 (q, *J* = 7.7 Hz, 1H), 2.51 – 2.36 (m, 1H), 2.35 – 2.24 (m, 1H), 2.07 (dd, *J* = 6.1, 4.9 Hz, 1H), 2.00 – 1.85 (m, 2H), 1.45 (s, 3H), 1.36 (d, *J* = 7.7 Hz, 3H), 1.32 (s, 3H), 1.16 (d, *J* = 11.0 Hz, 1H), 0.88 (s, 3H).
^{13}C NMR (101 MHz, Methanol-*d*$_4$) δ 88.9, 80.2, 52.5, 40.7, 39.3, 36.0, 33.8 (<u>C</u>HB, broad signal), 28.8, 27.4, 27.2, 24.3, 14.9.
Analytical data are in accordance with those reported in the literature[3].

<u>Synthesis of 2-(iodomethyl)-4,4,5,5-tetramethyl-1,3,2- dioxaborolane **14**</u>
Prepared according to the literature[7].
A stirred solution of 2-isopropoxy-4,4,5,5-tetramethyl-1,3,2-dioxaborolane **11b** (6 mL, 29.4 mmol, 1.0 equiv) and chloroiodomethane (2.4 mL, 32.9 mmol, 1.1 equiv) in anhydrous tetrahydrofuran (30 mL) was cooled to –78°C. *n*-Buthyl lithium 2.5 M (13 mL, 32.5 mmol, 1.1 equiv) was added dropwise and after stirring for 30 min chlorotrimethylsilane (4.5 ml, 35.5 mmol, 1.2 equiv) was added (dropwise) at the same temperature. After 10 min the flask was removed from the cooling bath and the contents were allowed to stir for 15 h at room temperature. According to GC-MS analysis the reaction mixture contained iodo-/chloro-substituted products in a 1:5 ratio, so the crude mixture was forwarded to the next reaction without purification.
GC/MS m/z = 176.1 (**14**-Cl), 268.0 (**14**-I)
Halogen exchange reaction for the synthesis of **14** was based on a slightly modified literature example[8].
The crude mixture was dissolved in acetone (40 mL), NaI (8.8 g, 58.7 mmol, 2 equiv) was added and mixture was refluxed for 2 h, cooled to room temperature and evaporated. The residue was dissolved in Et_2O (30 mL) and H_2O (30 mL). The layers were separated, the aueous phase was extracted with Et_2O, and the combined organic phases were washed with $Na_2S_2O_3$, then dried over Na_2SO_4, filtered, evaporated and used in the next step without additional purification.
GC/MS m/z = 268.0 (**14**-I)

<u>(+)-Pinanediol 2-(iodomethyl)boronate **15**.</u>
To the crude mixture of **14** in anhydrous tetrahydrofuran (30 mL) was added (+)-pinanediol (7.51 g, 44.1 mmol, 1.5 equiv). The mixture was stirred for 20 h at room temperature. Then the solution was evaporated and the crude mixture was purified by flash chromatography on silica gel eluting with Hexane:EtOAc (20:1) to provide **15** (5.71 g, 61 % over three steps) as a colorless oil.
^{1}H NMR (300 MHz, Chloroform-*d*) δ 4.36 (dd, *J* = 8.8, 2.0 Hz, 1H), 2.41 – 2.29 (m, 1H), 2.28 – 2.18 (m, 3H), 2.08 (dd, *J* = 6.1, 4.9 Hz, 1H), 1.97 – 1.82 (m, 2H), 1.40 (s, 3H), 1.29 (s, 3H), 1.25 (d, *J* = 4.4 Hz, 1H), 0.84 (s, 3H)
^{13}C NMR (101 MHz, Chloroform-*d*) δ 86.7, 78.7, 51.5, 39.4, 38.6, 35.4, 28.4, 27.2, 26.4, 24.1, -23.5 (<u>C</u>HB, broad signal).
GC/MS m/z = 320.1

<u>(+)-Pinanediol 2-(*bis*trimethylsilylaminomethyl)boronate **9c**.</u>

The procedure for the preparation of **9c** was the same as for **9a**: 2-iodomethylboronic ester **15** (268 mg, 0.84 mmol, 1.0 equiv) in anhydrous tetrahydrofuran (7 mL), lithium *bis*(trimethylsilyl)amide 1 M (840 µL, 0.84 mmol, 1.0 equiv). After the reaction was warmed up to room temperature, it was used in the next step without purification and cleavage of TMS groups.
GC/MS m/z = 353.3 (M), 338.2 (M−Me), 280.2 (M−$SiMe_3$).

<u>(+)-Pinanediol ((*p*-methoxybenzyl)oxy)methylboronate **16**</u>
This was prepared according to a modified literature method[9]
A solution of *n*-buthyl lithium 1.9 M (9.9 mL, 18.8 mmol 1.1 equiv) was added slowly to a solution of *p*-methoxybenzyl alcohol (2.3 mL, 2.56 g, 18.5 mmol, 1.05 equiv) and a few crystals of oven dried 1,10-phenanthroline indicator in anhydrous tetrahydrofuran (25 mL) under argon at 0 °C. The resulting solution of lithium *p*-methoxybenzyl oxide was stirred at 0 °C during the addition of **15** in 10 mL dry tetrahydrofuran via a syringe. Dimethyl sulfoxide (1.34 mL, 18.8 mmol, 1.1 equiv) was added, and the mixture was stirred overnight at room temperature, then it was refluxed for 4 h, cooled to room temperature, and evaporated. Saturated NH_4Cl (40 mL) and light petroleum ether (40 mL) was added, and the phases were separated. The aqueous phase was extracted one more time with light petroleum ether (20 mL). The combined organic phase was washed with brine (20 mL), dried over Na_2SO_4, filtered, and evaporated in vacuo. The crude mixture was purified by flash chromatography on silica gel eluting with Hexane:EtOAc (20:1) – (8:1) to provide **16** (2.87 g, 51%) as a colorless oil.
^{1}H NMR (300 MHz, Chloroform-*d*) δ 7.33 – 7.22 (m, 2H, overlaps with solvent), 6.92 – 6.81 (m, 2H), 4.46 (d, *J* = 2.3 Hz, 2H), 4.32 (dd, *J* = 8.7, 1.8 Hz, 1H), 3.80 (s, 3H), 3.30 (d, *J* = 0.9 Hz, 2H), 2.39 – 2.25 (m, 1H), 2.24 – 2.16 (m, 1H), 2.10 – 2.03 (m, 1H), 1.96 – 1.85 (m, 2H), 1.40 (s, 3H), 1.28 (s, 3H), 1.15 (d, *J* = 10.8 Hz, 1H), 0.83 (s, 3H).
^{13}C NMR (101 MHz, Chloroform-*d*) δ 159.3, 130.4, 130.0, 113.8, 86.5, 78.3, 75.5, 56.5 (<u>C</u>HB, broad signal), 55.4, 51.3, 39.6, 38.2, 35.3, 28.7, 27.2, 26.6, 24.1.
GC/MS m/z = 330.2
Analytical data are in accordance with those reported in the literature[8].

<u>(+)-Pinanediol ((*p*-methoxybenzyl)oxy)ethylboronate **17**</u>
The method used was based on homologation reaction reported in the literature[10]
A solution of boronic ester **16** (2.86 g, 8.6 mmol, 1.0 equiv) and chloroiodomethane (1.9 mL, 26.0 mmol, 3.0 equiv) was dissolved in anhydrous Et_2O (44 mL, 0.2 M) under an atmosphere of argon. The reaction mixture was cooled to –100 °C (EtOH/liquid nitrogen bath) and *n*-buthyl lithium 1.9 M (13.2 mL, 25.0 mmol, 2.90 equiv) was added dropwise to the reaction mixture at −100 °C. The reaction mixture was stirred for 10 min at the same temperature, removed from the cooling bath and stirred for 1 h at room temperature. Water (20 mL) and light petroleum ether (40 mL) was added, and the phases were separated. The aqueous phase was extracted one more time with light petroleum ether (20 mL). The combined organic phase was washed with brine (20 mL), dried over Na_2SO_4, filtered, and evaporated in vacuo. The crude mixture was purified by flash chromatography on silica gel eluting with Hexane:EtOAc (20:1) – (8:1) to provide **17** (2.25 g, 76%) as a colorless oil.
^{1}H NMR (300 MHz, Chloroform-*d*) δ 7.32 – 7.21 (m, 2H, overlaps with $CHCl_3$ residue signal), 6.92 – 6.81 (m, 2H), 4.44 (s, 2H), 4.25 (dd, *J* = 8.7, 1.9 Hz, 1H), 3.80 (s, 3H), 3.61 (t, *J* = 7.9 Hz, 2H), 2.38 – 2.26 (m, 1H), 2.24 – 2.13 (m, 1H), 2.03 (t, *J* = 5.5 Hz, 1H), 1.94 – 1.78 (m, 2H), 1.38 (s, 3H), 1.28 (s, 3H), 1.25 (t, *J* = 7.9 Hz, 2H), 1.13 (d, *J* = 10.9 Hz, 1H), 0.84 (s, 3H).
GC/MS m/z = 344.2
Analytical data are in accordance with those reported in the literature[11].

<u>(+)-Pinanediol (1*S*)-(1-chloro-3-((*p*-methoxybenzyl)oxy)propyl)boronate **18**.</u>
The same method was used as for **13a**: dichloromethane (230 µL, 3.59 mmol, 5.0 equiv) in anhydrous tetrahydrofuran (10 mL), *n*-butyl lithium 2.5 M (430 µL, 1.08 mmol, 1.5 equiv), pinanediol alkylboronate **17** (249 mg, 0.723 mmol, 1.0 equiv) in anhydrous tetrahydrofuran (5 mL), $ZnCl_2$ 1M (1.3 mL, 1.3 mmol, 1.8 equiv). The residue was purified by flash chromatography on silica gel eluting with Hexane:EtOAc (20:1) – Hexane:EtOAc (4:1) to provide **18** (262 mg, 92%) as a colorless oil.

^{1}H NMR (400 MHz, Chloroform-*d*) δ 7.30 – 7.23 (m, 2H, overlaps with $CHCl_3$ residue signal), 6.91 – 6.84 (m, 2H), 4.45 (d, *J* = 2.8 Hz, 2H), 4.31 (dd, *J* = 8.7, 1.8 Hz, 1H), 3.80 (s, 3H), 3.67 (dd, *J* = 8.0, 6.0 Hz, 1H), 3.63 – 3.57 (m, 2H), 2.39 – 2.27 (m, 1H), 2.25 – 2.14 (m, 2H), 2.12 – 2.02 (m, 2H), 1.94 – 1.85 (m, 2H), 1.33 (s, 3H), 1.28 (s, 3H), 1.18 (d, *J* = 11.1 Hz, 1H), 0.82 (s, 3H).
^{13}C NMR (101 MHz, Chloroform-*d*) δ 159.3, 130.6, 129.5, 113.9, 86.8, 78.6, 72.9, 66.8, 55.4, 51.4, 40.4 (<u>C</u>HB, broad signal), 39.5, 38.3, 35.4, 34.5, 28.4, 27.2, 26.4, 24.1.
GC/MS m/z = 392.2
Analytical data are in accordance with those reported in the literature[10].

<u>(+)-Pinanediol (1*R*)-(1-*bis*trimethylsilylamino)-3-((*p*-methoxybenzyl)oxy)propyl) boronate **9d**</u>
The same method was used as for **9a**: α-chloroboronic ester **18** (218 mg, 0.444 mmol, 1.0 equiv) in anhydrous tetrahydrofuran (5 mL), lithium *bis*(trimethylsilyl)amide 1 M (490 µL, 0.600 mmol, 1.0 equiv). The reaction mixture was used in the next step without purification and cleavage of TMS groups.
GC/MS m/z = 444.3 (M−$SiMe_3$)

<u>(+)-Pinanediol-((*p*-methoxybenzyl)oxy)propylboronate **19**</u>
The same method was used as for **17**: boronic ester **17** (700 mg, 2.03 mmol, 1.0 equiv) and chloroiodomethane (450 µL, 6.16 mmol, 3.0 equiv), anhydrous Et_2O (10 mL, 0.2 M), *n*-buthyl lithium 2.5 M (2.4 mL, 6.0 mmol, 2.95 equiv) was added dropwise to the reaction mixture at −100 °C. The crude mixture was purified by flash chromatography on silica gel eluting with Hexane:EtOAc (20:1) – (8:1) to provide **19** (395 mg, 54 %) as a colorless oil.
^{1}H NMR (300 MHz, Chloroform-*d*) δ 7.29 – 7.22 (m, 2H, overlaps with $CHCl_3$ residue signal), 6.89 – 6.83 (m, 2H), 4.43 (s, 2H), 4.23 (dd, *J* = 8.7, 2.0 Hz, 1H), 3.80 (s, 3H), 3.43 (t, *J* = 6.7 Hz, 2H), 2.37 – 2.25 (m, 1H), 2.23 – 2.13 (m, 1H), 2.02 (dd, *J* = 6.1, 5.0 Hz, 1H), 1.93 – 1.85 (m, 1H), 1.85 – 1.68 (m, 3H), 1.35 (s, 3H), 1.28 (s, 3H), 1.09 (d, *J* = 10.8 Hz, 1H), 0.91 – 0.79 (m, 5H).
^{13}C NMR (101 MHz, Chloroform-*d*) δ 159.2, 131.0, 129.4, 113.8, 85.5, 77.7, 72.5, 72.0, 55.4, 51.4, 39.7, 38.3, 35.7, 28.8, 27.2, 26.6, 24.4, 24.1, 7.0 (<u>C</u>HB, broad signal).
GC/MS m/z = 358.3

<u>(+)-Pinanediol (1*S*)-(1-chloro-4-((*p*-methoxybenzyl)oxy)butyl)boronate **20**.</u>
The same method was used as for **13a**: dichloromethane (140 µL, 2.18 mmol, 5.0 equiv) in anhydrous tetrahydrofuran (7 mL), *n*-buthyl lithium 2.5 M (260 µL, 0.650 mmol, 1.5 equiv), pinanediol alkylboronate **19** (155 mg, 0.433 mmol, 1.0 equiv) in anhydrous tetrahydrofuran (5 mL), $ZnCl_2$ 1 M (780 µL, 0.780 mmol, 1.8 equiv). The residue was purified by flash chromatography on silica gel eluting with Hexane:EtOAc (20:1) – Hexane:EtOAc (4:1) to provide **20** (97 mg, 55%) as a colorless oil.
^{1}H NMR (400 MHz, Chloroform-*d*) δ 7.30 – 7.21 (m, 2H, overlaps with $CHCl_3$ residue signal), 6.92 – 6.83 (m, 2H), 4.43 (s, 2H), 4.36 (dd, *J* = 8.8, 1.9 Hz, 1H), 3.80 (s, 3H), 3.52 – 3.44 (m, 3H), 2.40 – 2.30 (m, 1H), 2.28 – 2.20 (m, 1H), 2.08 (dd, *J* = 6.0, 4.9 Hz, 1H), 2.04 – 1.95 (m, 1H), 1.95 – 1.80 (m, 4H), 1.79 – 1.68 (m, 1H), 1.41 (s, 3H), 1.29 (s, 3H), 1.18 (d, *J* = 11.0 Hz, 1H), 0.84 (s, 3H).
^{13}C NMR (101 MHz, Chloroform-*d*) δ 159.3, 130.8, 129.4, 113.9, 86.9, 78.7, 72.6, 69.5, 55.4, 51.3, 43.0 (<u>C</u>HB, broad signal), 39.5, 38.4, 35.4, 31.1, 28.6, 27.6, 27.2, 26.5, 24.1.
GC/MS m/z = 406.1

<u>(+)-Pinanediol (1*R*)-(1-*bis*trimethylsilylamino)-4-((*p*-methoxybenzyl)oxy)butyl)boronate **9e**</u>
The same method was used as for **9a**: α-chloroboronic ester **20** (184 mg, 0.271 mmol, 1.0 equiv) in anhydrous tetrahydrofuran (7 mL), lithium *bis*(trimethylsilyl)amide 1 M (270 µL, 0.271 mmol, 1.0 equiv). After the reaction was warmed to room temperature, it was used in the next step without purification and cleavage of TMS groups.
GC/MS m/z = 458.3 (M−$SiMe_3$)

<u>(+)-Pinanediol-2-(*tert*-butoxycarbonyl)ethyl boronate **21**.</u>
A literature procedure was used for the synthesis[12].
(+)-Pinanediol iodomethaneboronate **15** (1.272 g, 3.98 mmol, 1.0 equiv) and *tert*-butyl acetate (640 µL, 4.77 mmol, 1.2 equiv) were dissolved in anhydrous tetrahydrofuran (7 mL) and cooled to

–78°C. In a separate flask, fresh LDA was prepared by treating diisopropylamine (670 µL, 4.78 mmol, 1.2 equiv) with *n*-butyllithium 2.5 M (1.8 mL, 4.50 mmol, 1.1 equiv) in THF (4 mL) at −78°C, followed by gradual warming to room temperature over 1 h. The formed LDA solution was slowly added via syringe at −78°C under argon flow over a 30 min period. After leaving to warm up to room temperature overnight, the reaction mixture was partitioned between light petroleum ether (30 mL) and saturated NH_4Cl (30 mL). The aqueous layer was extracted with light petroleum ether (2x20 mL) and the combined organic phases were washed with brine (20 mL), dried over Na_2SO_4, filtered and evaporated. The residue was purified by flash chromatography on silica gel eluting with Hexane:EtOAc (8:1) – Hexane:EtOAc (4:1) to provide **21** (836 mg, 68 %) as a colorless oil.
^{1}H NMR (400 MHz, Chloroform-*d*) δ 4.25 (dd, *J* = 8.8, 2.0 Hz, 1H), 2.36 (t, *J* = 7.5 Hz, 2H), 2.33 – 2.27 (m, 1H), 2.24 – 2.13 (m, 1H), 2.02 (t, *J* = 5.6 Hz, 1H), 1.92 – 1.86 (m, 1H), 1.86 – 1.79 (m, 1H), 1.43 (s, 9H), 1.36 (s, 3H), 1.28 (s, 3H), 1.18 (d, *J* = 10.9 Hz, 1H), 1.02 (t, *J* = 7.5 Hz, 2H), 0.83 (s, 3H).
^{13}C NMR (101 MHz, Chloroform-*d*) δ 174.2, 85.7, 80.0, 77.9, 51.4, 39.6, 38.3, 35.6, 30.2, 28.3, 27.2, 26.5, 24.2, 5.9 (<u>C</u>HB, broad signal).
GC/MS m/z = 252.1 (M−*t*Bu)
Analytical data are in accordance with those reported in the literature for the other enantiomer[11].

<u>(+)-Pinanediol (1*S*)-(1-chloro)-3-(*tert*-butoxycarbonyl)propyl boronate **22**</u>.
The same method was used as for **13a**: dichloromethane (480 µL, 7.49 mmol, 5.0 equiv) in anhydrous tetrahydrofuran (10 mL), *n*-buthyl lithium 2.5 M (900 µL, 2.25 mmol, 1.5 equiv), pinanediol alkylboronate **21** (465 mg, 1.51 mmol, 1.0 equiv) in anhydrous tetrahydrofuran (5 mL), $ZnCl_2$ 1 M (2.7 mL, 2.7 mmol, 1.8 equiv). The residue was purified by flash chromatography on silica gel eluting with Hexane:EtOAc (8:1) – Hexane:EtOAc (4:1) to provide **22** (530 mg, 98 %) as a colorless oil (NMR showed few percents of **21**).
^{1}H NMR (400 MHz, Chloroform-*d*) δ 4.36 (dd, *J* = 8.8, 1.9 Hz, 1H), 3.52 (dd, *J* = 9.3, 5.1 Hz, 1H), 2.49 – 2.41 (m, 2H), 2.40 – 2.31 (m, 1H), 2.28 – 2.13 (m, 2H), 2.11 – 2.00 (m, 2H), 1.95 – 1.86 (m, 2H), 1.44 (s, 9H), 1.42 (s, 3H), 1.29 (s, 3H), 1.17 (d, *J* = 11.1 Hz, 1H), 0.84 (s, 3H).
GC/MS m/z = 356.9
Analytical data are in accordance with those reported in the literature for the other enantiomer[11].

<u>(+)-Pinanediol (1*R*)-(1-*bis*trimethylsilylamino)-3-(*tert*-butoxycarbonyl)propyl boronate **9f**</u>.
The same method was used as for **9a**. α-Chloroboronic ester **22** (233 mg, 0.653 mmol, 1 equiv) in anhydrous tetrahydrofuran (5 mL), lithium *bis*(trimethylsilyl)amide 1M (655 µL, 0.655 mmol, 1 equiv). The reaction mixture was used in the next step without purification and cleavage of TMS groups.
GC/MS m/z = 408.4 (M−$SiMe_3$)

<u>Pinacol dichloromethaneboronate **23**</u>
This compound was synthesized according to a previously reported method[13]
n-Butyl lithium 2.2 M (10 mL, 22 mmol) was added dropwise to the solution of absolute methylene dichloride (1.6 mL, 25.0 mmol, 1.1 equiv) dissolved in 40 mL of absolute THF under nitrogen atmosphere at −100°C. After 40 min at −100°C, 2-isopropoxy boronic acid pinacol ester (5.0 mL, 24.5 mmol, 1.1 equiv) was added dropwise. The reaction was allowed to perform for another 40 min at −100°C. Thereafter, the reaction was quenched with 5 N HCl (5 mL). The solution was allowed to reach room temperature. The organic layer was separated and the aqueous layer was extracted with ether (2×10 mL). The combined organic layers were dried over anhydrous Na_2SO_4, filtered and evaporated to obtain the crude product as a white solid. It was used directly in the next reaction.
GC/MS m/z = 210.0

<u>(+)-Pinanediol dichloromethaneboronate **24**</u>
A solution of the crude product (4.64 g, 22.0 mmol, based on the theoretical amount of 100 %) and (+)-pinanediol (3.75 g, 22.0 mmol) was stirred in anhydrous tetrahydrofuran (40 mL) for 18 h at room temperature. The residue was purified by flash chromatography on silica gel eluting with Hexane:EtOAc (20:1) to provide **24** (4.44 g, 77 %).

^1H NMR (300 MHz, Chloroform-*d*) δ 5.40 (s, 1H), 4.47 (dd, *J* = 8.8, 1.9 Hz, 1H), 2.45 – 2.32 (m, 1H), 2.32 – 2.24 (m, 1H), 2.13 (dd, *J* = 6.1, 4.8 Hz, 1H), 2.00 – 1.89 (m, 2H), 1.46 (s, 3H), 1.31 (s, 3H), 1.22 (d, *J* = 11.1 Hz, 1H), 0.85 (s, 3H).
GC/MS m/z = 262.1
Analytical data are in accordance with those reported in the literature[12].

(+)-Pinanediol (1-aminobenzyl)boronate hydrochloride **9g**
A previously reported method was used for the first step of the synthesis[3].
Phenylmagnesium bromide 0.7 M (3.1 mL, 2.15 mmol, 1.2 equiv) was added dropwise at −78°C to a solution of (+)-pinanediol dichloromethyl boronate **24** (470 mg, 1.79 mmol, 1.0 equiv) in anhydrous diethyl ether (10 mL). After 15 min $ZnCl_2$ 1 M (1.8 mL, 1.8 mmol, 1.0 equiv) was slowly added at this temperature. The reaction mixture was allowed to warm up to room temperature over 2 hours. The inorganic precipitate was filtered off and the solvents were removed in vacuo. The residue was dissolved in pentane, washed with water and dried over Na_2SO_4. Removal of the solvent gives a product which was forwarded to the next step without purification.
GC/MS m/z = 304.1
In the next step the same method was used as for **9a**: α-chloroboronic ester **24** (544 mg, 1.78 mmol, 1.0 equiv) in anhydrous tetrahydrofuran (20 mL), lithium *bis*(trimethylsilyl)amide 1 M (1.8 mL, 1.8 mmol, 1.0 equiv).
GC/MS m/z = 356.2 (M− $SiMe_3$)
Then HCl/dioxane 4 M (1.5 mL, 6 mmol, 3.3 equiv) and **9g** was isolated as a white powder (210 mg, 37% over three steps).
^1H NMR (300 MHz, Methanol-d_4) δ 7.48 – 7.30 (m, 5H), 4.56 – 4.45 (m, 1H), 4.07 (s, 1H), 2.51 – 2.32 (m, 1H), 2.30 – 1.98 (m, 2H), 1.96 – 1.73 (m, 2H), 1.48 – 1.38 (m, 3H), 1.33 – 1.25 (m, 3H), 1.13 – 0.91 (m, 1H), 0.87 (s, 3H).
Analytical data are in accordance with those reported in the literature for one of the enantiomers[3].

3. Methodology for assessing inhibition of mammalian serine proteases by compound 3j.

Assays for inhibition of trypsin activity (TPCK treated, from bovine pancreas; Sigma-Aldrich T1426) were based on cleavage of the fluorogenic peptide substrate Boc-QAR-AMC (Sigma-Aldrich B4153). which contains the fluorescent 7-amino-4-methyl coumarin (AMC) group. Fluorescence of this is quenched by the amide bond formed between its amino group and the arginine carboxyl group. Cleavage of this bond results in release of AMC and increased fluorescence. A stock solution of trypsin (1 mg/ml; 42 μM) was prepared in 1 mM HCl, 2 mM $CaCl_2$. Compound **3j**, stored as a 1 mM stock solution in 100% anhydrous DMSO was diluted to a final concentration of 10 μM in assay buffer (25 mM HEPES pH 7.4, 15 mM $CaCl_2$). For the inhibition assays, 5 ng/ml (0.21 μM) trypsin in 100 μl assay buffer was dispensed into the wells of white 96-well microwell plates (Nunc) and pre-incubated with 10 μM compound **3j** for 5 min before addition of substrate (10 μM final). Phenylmethylsulfonyl fluoride (PMSF; diluted from a 100 mM stock in 100% isopropanol) was used as a positive control for enzyme inhibition. Fluorescence increase was monitored over time at 22°C using a SpectraMax M5e plate reader fitted with SoftMax Pro 6.3 software, with readings taken at 3 min intervals for up to 45 min using excitation and emission values of 380 nm and 460 nm respectively. All measurements were performed in duplicate.

Assays for inhibition of α-chymotrypsin activity (TLCK treated, from bovine pancreas; Sigma-Aldrich C3142) were based on cleavage of the fluorogenic peptide substrate N-Suc-LLVY-AMC (Sigma-Aldrich S6510). A stock solution of chymotrypsin (1mg/ml, 40 μM) was made up in 1 mM HCl, 2 mM $CaCl_2$. For inhibition assays, 100 μl chymotrypsin at 100 ng/ml (4 nM) in assay buffer was added to wells of white 96-well microwell plates (Nunc), and pre-incubated with compound **3j** at final concentrations of 10 μM, 100 μM and 200 μM EP_1200 for 5 min before adding substrate (10 μM final). Fluorescence increase was monitored continuously with time as above. PMSF was used as a positive inhibitor control as above. All measurements were performed in duplicate.

Assays for assessing inhibition of elastase activity (from porcine pancreas; Sigma-Aldrich E7885) were based on cleavage of the fluorogenic peptide substrate MeOSuc-AAPV-AMC (Sigma-Aldrich 324740). Elastase was dissolved in 1 mM HCl, 2 mM $CaCl_2$ to produce a stock solution of 1 mg/ml (38.6 μM). For inhibition assays, 100 μl of 1 μg/ml (38.6 nM) elastase in 25 mM Tris-HCl pH 8.2, 15 mM $CaCl_2$ was dispensed into wells of white 96-well microwell plates (Nunc), and pre-incubated for 5 min with compound **3j** at concentrations ranging from 0-20 μM before addition of substrate (10 μM final). Fluorescence increase was monitored continuously with time as above. PMSF was used as a positive inhibitor control as above. All measurements were performed in duplicate.

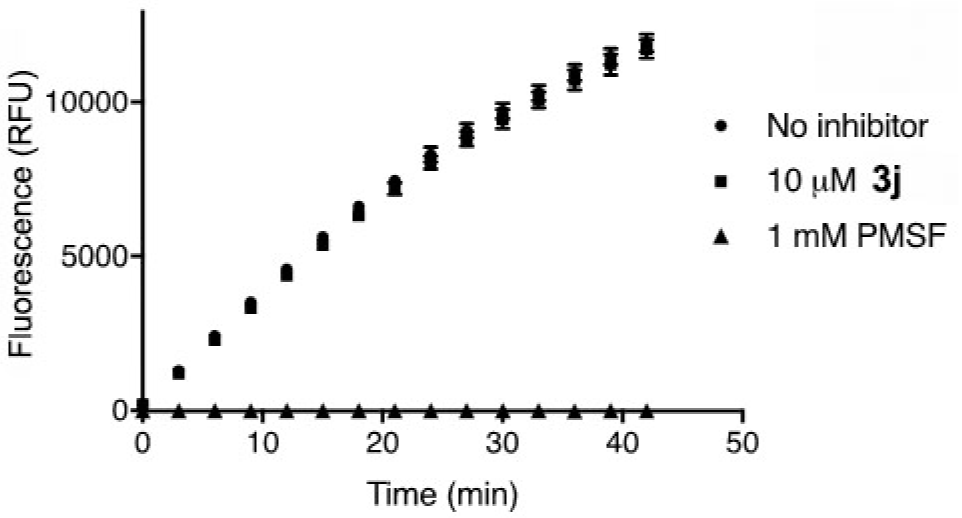

Fig. S1. Compound 3j shows low potency against mammalian trypsin/chymotrypsin family serine proteases.

Progress curves depicting cleavage of fluorogenic substrate Boc-QAR-AMC by bovine trypsin in the presence of various concentrations of compound **3j,** or PMSF (control inhibitor). Compound **3j** had no detectable effect on trypsin activity at this concentration, indicating an IC_{50} value >>10 μM. Values shown are averages of two independent experiments. Error bars, S.D.

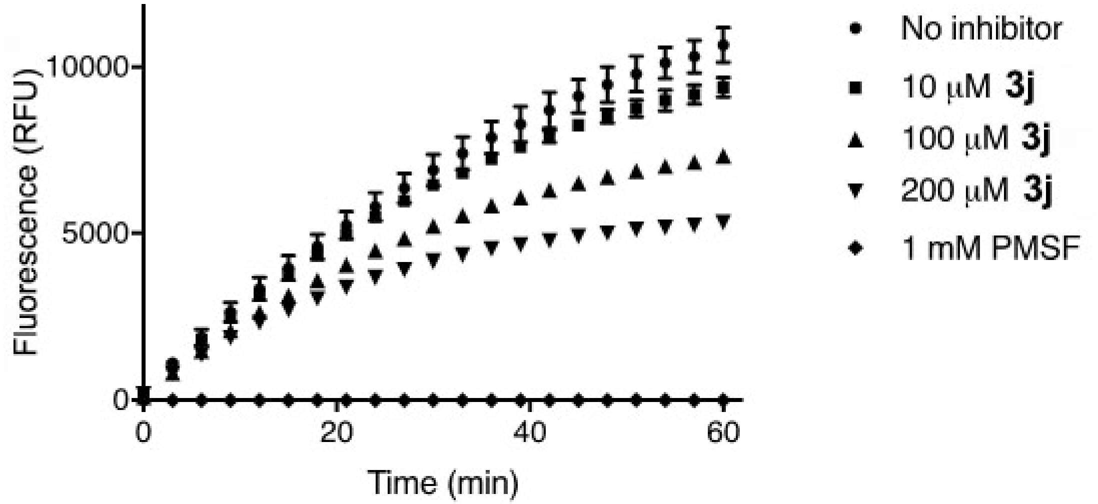

Fig. S2. Compound 3j shows low potency against mammalian trypsin/chymotrypsin family serine proteases.

Progress curves depicting cleavage of fluorogenic substrate N-Suc-LLVY-AMC by bovine α-chymotrypsin in the presence of various concentrations of compound **3j** or PMSF (control inhibitor). The progress curves indicate an IC_{50} value >200 μM under the conditions used. Values shown are averages of two independent experiments. Error bars, S.D.

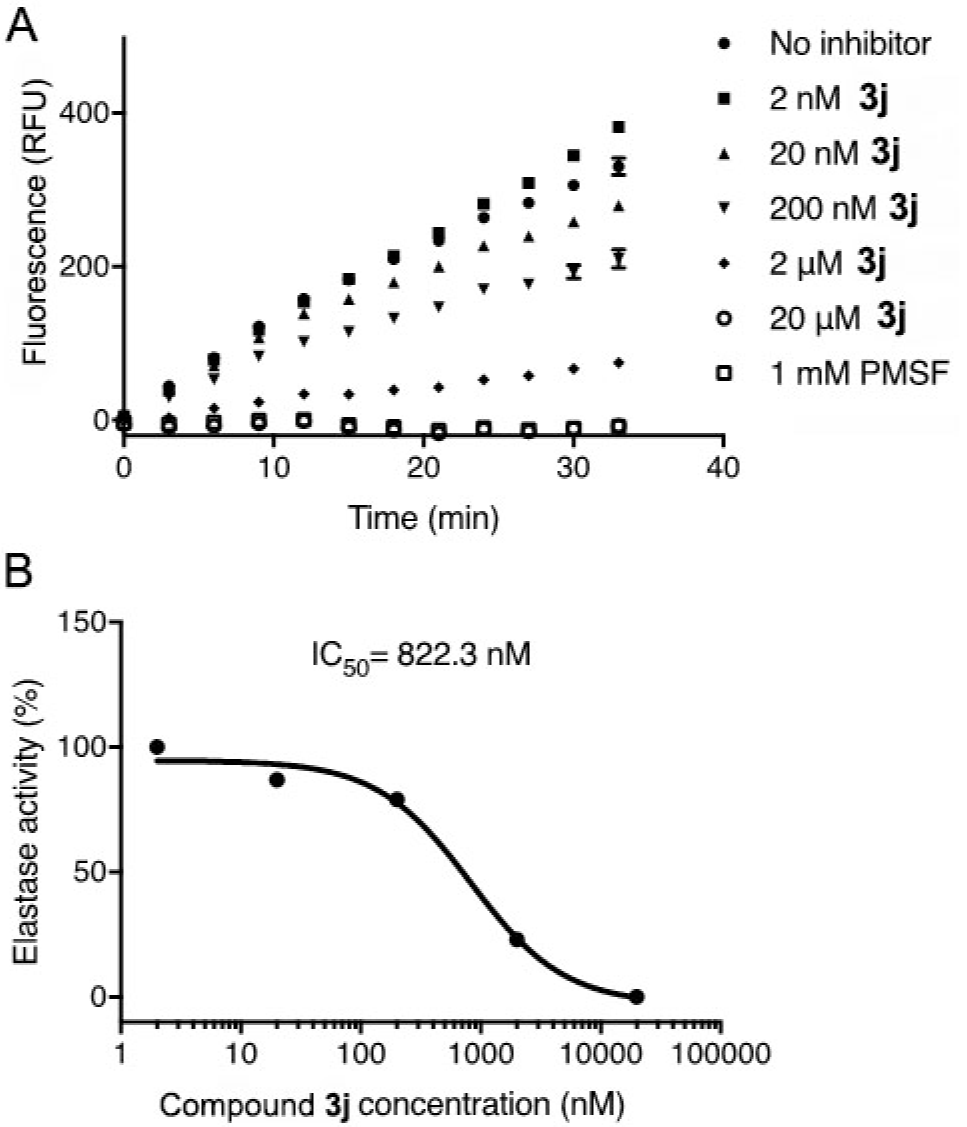

Fig. S3. Compound 3j shows low potency against mammalian trypsin/chymotrypsin family serine proteases.

(A) Progress curves depicting cleavage of fluorogenic substrate MeOSuc-AAPV-AMC by porcine elastase in the presence of various concentrations of compound **3j** or PMSF (control inhibitor). Values shown are averages of two independent experiments. Error bars, S.D. *(B)* Dose-response curve for inhibition of elastase by compound **3j**. Values for degree of inhibition were obtained using the linear part of the progress curves (<10% substrate depletion) shown in *(A)*. The estimated IC_{50} value under the conditions used is greater than 100-fold higher than that for PfSUB1 (~5.7 nM; see Table 1 of main manuscript) .

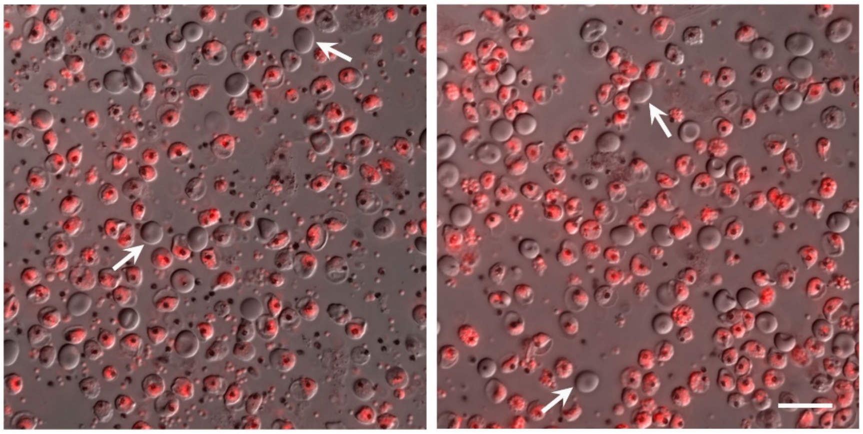

Fig. S4. Treatment of *P. falciparum* schizonts with egress-inhibitory concentrations of compound 3j does not prevent uptake of the vital mitochondrial dye MitoTracker Red CMXRos.

Combined DIC/fluorescence images of *P. falciparum* schizonts following incubation at 37°C for 1 h in medium containing either control vehicle only (DMSO, 1% v/v) or compound **3j** (10 μM), followed by treatment for 15 min at 37°C with MitoTracker Red CMXRos (20 nM). Note that the few uninfected red blood cells in the schizont preparations (examples indicated with white arrows) do not stain with the dye, consistent with the dye localizing to parasite mitochondria. Scale bar, 20 μm.

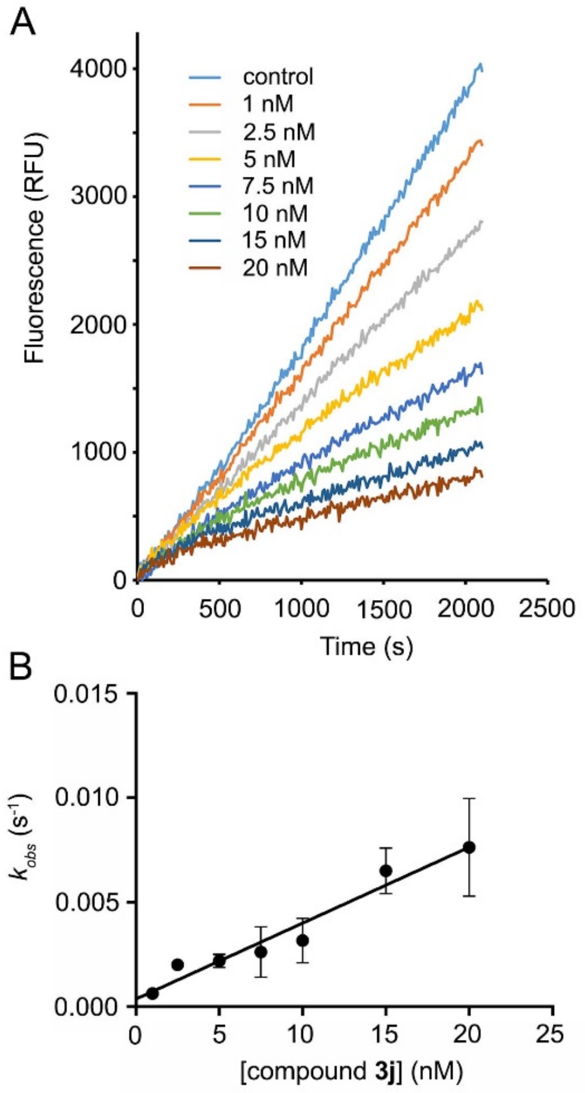

Fig. S5. Inhibition of rPfSUB1 by compound 3j is time-dependent and slowly reversible.

(*A*) Typical progress curve showing the effects of varying concentrations of compound **3j** on the rate of cleavage of fluorogenic substrate SERA4st1F-6R12. Rate of fluorescence increase in the absence of inhibitor (control) was linear over the time course of the assay. (*B*) Plot of the pseudo first order constant k_{obs} calculated from curve-fitting of the progress curves to an equation describing time-dependent inhibition, plotted against concentration of inhibitor **3j**. The fitted straight line was calculated by least squares linear regression. The intercept with the Y axis yields an apparent first order rate constant k_{off} of 3.7 x 10^{-4} s^{-1}, whilst the slope yields an apparent second order rate constant k_{on} of 3.6 x10^5 $M^{-1}.s^{-1}$. Error bars, S.D. (calculated from curve-fitting progress curves from 3 independent experiments).

Table S1. SAR of ketoamide inhibitors with variations at the P4 position.

The previously developed peptidic ketoamide series **1**[14] was extended with a set of new compounds **2a-j** incorporating a range of unnatural amino acids instead of isoleucine at the P4 position.

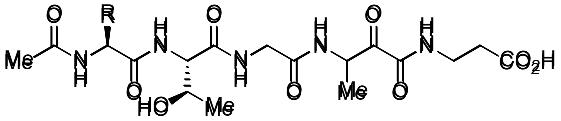

Inhibitor	R	IC_{50} or degree of rPfSUB1 inhibition at 10 µM (%)[a]
**1**	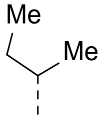	IC_{50} 900 ± 200 nM[14]
**2a** (EP_272)	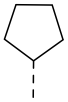	IC_{50} 370 ±3.35 nM<br>Inhibition at 10 µM 97%
**2b** (EP_307)	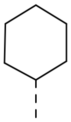	Inhibition at 10 µM 75%
**2c** (EP_346)	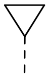	Inhibition at 10 µM 45%
**2d** (EP_337)	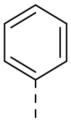	Inhibition at 10 µM 56%
**2e** (EP_245)	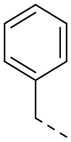	Inhibition at 10 µM 38%
**2f** (EP_243)	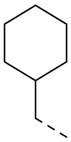	Inhibition at 10 µM 23%
**2g** (EP_347)	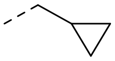	Inhibition at 10 µM 78%
**2h** (EP_323)	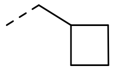	Inhibition at 10 µM 73%
**2i** (EP_332)	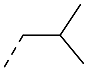	Inhibition at 10 µM 78%
**2j** (EP_244)	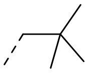	Inhibition at 10 µM 71%

[a] Potency evaluated *in vitro* against recombinant PfSUB1 (rPfSUB1) as described in the main manuscript text.

Movie legends

Movie S1 (separate file). Rapid egress of *P. falciparum* merozoites following release of a C2-mediated egress block. *P. falciparum* schizonts expressing an mNeonGreen fusion of the parasitophorous vacuole membrane (PVM) protein EXP2 were treated for ~2 h with the reversible PKG inhibitor C2 (1 μM). The schizonts were then washed twice, suspended in fresh warm medium without C2 and immediately observed by dual DIC/fluorescence time-lapse video microscopy, capturing images at 5 s intervals. As expected, the schizonts underwent normal 'explosive' egress, initiating at ~6 min 30 s.

Movie S2 (separate file). Treatment with compound 3j prevents egress of *P. falciparum* merozoites even following compound washout. *P. falciparum* schizonts expressing an mNeonGreen fusion of the PVM protein EXP2 were treated for ~2 h with compound **3j** (10 μM). The schizonts were then washed twice, suspended in fresh warm medium with no drug, then immediately observed by dual DIC/fluorescence time-lapse video microscopy, capturing images at 5 s intervals. In contrast to washout of the reversible PKG inhibitor C2 (Movie S1), no egress or PVM rupture takes place during the 30 min monitoring period, indicating that egress inhibition by **3j** is effectively irreversible over this time period.

SI References

1. S. Höck, R. Martib, R. Riedla, M. Simeunovic, Thermal Cleavage of the Fmoc Protection Group. *CHIMIA International Journal for Chemistry*. 64, 200-202(3), (2010)
2. S. D. Dattoli, R. De Marco, M. Baiula, S. Spampinato, A. Greco, A. Tolomelli, L. Gentilucci. Synthesis and assay of retro-$\alpha_4\beta_1$ integrin-targeting motifs. *European Journal of Medicinal Chemistry*. 73, 225- 232, (2014)
3. M. L. Winkler, E. A. Rodkey, M. A. Taracila, S. M. Drawz, C. R. Bethel, K. M. Papp-Wallace, K. M. Smith, Y. Xu, J. R. Dwulit-Smith, C. Romagnoli, E. Caselli, F. Prati, F. van den Akker, R. A. Bonomo. Design and Exploration of Novel Boronic Acid Inhibitors Reveals Important Interactions with a Clavulanic Acid-Resistant SHV β-lactamase *J. Med. Chem.* 56, 1084-1097, (2013)
4. O. V. Gozhina, J. S. Svendsen, T. Lejon. Synthesis and antimicrobial activity of α-aminoboronic-containing peptidomimetics. *Journal of Peptide Science*. 20, 20–24, (2014)
5. F. Debaene, J. A. Da Silva, Z. Pianowski, F. J. Duran, N. Winssinger. Expanding the scope of PNA-encoded libraries: divergent synthesis of libraries targeting cysteine, serine and metalloproteases as well as tyrosine phosphatases. *Tetrahedron*. 63, 6577–6586, (2007).
6. L. Dzhekieva, M. Rocaboy, F. Kerff, P. Charlier, E. Sauvage, R. F. Pratt. Crystal Structure of a Complex between the Actinomadura R39 DD-Peptidase and a Peptidoglycan-mimetic Boronate Inhibitor: Interpretation of a Transition State Analogue in Terms of Catalytic Mechanism. *Biochemistry*. 49, 6411–6419, (2010).
7. J. L. Farmer, H. N. Hunter, M. G. Organ. Regioselective Cross-Coupling of Allylboronic Acid Pinacol Ester Derivatives with Aryl Halides via Pd-PEPPSI-IPent. *J. Am. Chem. Soc.*134, 17470-17473, (2012).
8. T. Nakamura, K. Suzuki, M. Yamashita. Aluminabenzene-Rh and -Ir Complexes: Synthesis, Structure, and Application toward Catalytic C-H Borylation. *J. Am. Chem. Soc.*139, 17763-17766, (2017)
9. D. S. Matteson, R. Soundararajan, O. Chenpu Ho, W. Gatzweiler. (Alkoxyalkyl)boronic Ester Intermediates for Asymmetric Synthesis. *Organometallics.* 15*,* 152-163, (1996)
10. S. Balieu, G. E. Hallett, M. Burns, T. Bootwicha, J. Studley, V. K. Aggarwal. owards Ideality: The Synthesis of (+)-Kalkitoxin and (+)-Hydroxyphthioceranic Acid by Assembly-Line Synthesis. *J. Am. Chem. Soc.* 137, 4398-4403, (2015)
11. F. Dazhong, J. R. Lewis, L. Linos, *et al.* Novel cyclic boronate inhibitors of HCV replication. WO2009046098
12. P. Davoli, R. Fava, S. Morandi, A.Spaggiari, F. Prati. Enantioselective total synthesis of (−)-microcarpalide. *Tetrahedron*. 61, 4427–4436, (2005)
13. Y. Zhu, X. Zhao, X. Zhu, G. Wu, Y. Li, Y. Ma, Y. Yuan, J. Yang, Y. Hu, Li Ai, Q. Gao. Design, Synthesis, Biological Evaluation, and Structure-Activity Relationship (SAR) Discussion of Dipeptidyl Boronate Proteasome Inhibitors, Part I: Comprehensive Understanding of the SAR of α-Amino Acid Boronates. *J.Med.Chem.* 52, 4192–4199, (2009)
14. a) S. S. Kher, M. Penzo, S. Fulle, P. W. Finn, M. J. Blackman, A. Jirgensons. Substrate derived peptidic alpha-ketoamides as inhibitors of the malarial protease PfSUB1. *Bioorganic & Medicinal Chemistry Letters.* 24, 4486– 4489, (2014). b) C. Withers-Martineza, C. Suarez, S. Fulle, S. Kher, M. Penzo, J.-P. Ebejer, K. Koussis, F. Hackett, A. Jirgensons, P. Finn, M. J. Blackman. Plasmodium subtilisin-like protease 1 (SUB1): Insights into the active-site structure, specificity and function of a pan-malaria drug target. *International Journal for Parasitology*, 42, 597-612, (2012)
